# The Usage of Exon-Exon Splice Junctions for the Detection of Alternative Splicing using the REIDS model

**DOI:** 10.1038/s41598-018-26695-9

**Published:** 2018-05-29

**Authors:** Marijke Van Moerbeke, Adetayo Kasim, Ziv Shkedy

**Affiliations:** 1 Hasselt University, Interuniversity institute for biostatistics and statistical bioinformatics, Hasselt, 3500 Belgium; 2 Durham University, Wolfson Research Institute for Health and Wellbeing, Durham, United Kingdom; 3 Durham University, Department of Anthropology, Durham, United Kingdom

## Abstract

Alternative gene splicing is a common phenomenon in which a single gene gives rise to multiple transcript isoforms. The process is strictly guided and involves a multitude of proteins and regulatory complexes. Unfortunately, aberrant splicing events have been linked to genetic disorders. Therefore, understanding mechanisms of alternative splicing regulation and differences in splicing events between diseased and healthy tissues is crucial in advancing personalized medicine and drug developments. We propose a linear mixed model, Random Effects for the Identification of Differential Splicing (REIDS), for the identification of alternative splicing events using Human Transcriptome Arrays (HTA). For each exon, a splicing score is calculated based on two scores, an exon score and an array score. The junction information is used to rank the identified exons from strongly confident to less confident candidates for alternative splicing. The design of junctions was also discussed to highlight the complexity of exon-exon and exon-junction interactions. Based on a list of Rt-PCR validated probe sets, REIDS outperforms AltAnalyze and iGems in the % recall rate.

## Introduction

Alternative splicing (AS) was considered to be an uncommon phenomenon until microarray and high-throughput sequencing technology enabled whole genome expression profiling^1^. More than 90% of human genes exhibit multiple transcript isoforms due to exon enrichment or depletion in mRNA transcription^2–4^. Since transcript isoforms of a single gene are known to vary between tissues and even between developmental stages, alternative splicing has been proposed as a primary driver of evolution and phenotypic complexity in mammals^5–7^. Straying splice variants, however, have been linked to cancers such as mammary tumorigenesis and ovarian cancer^8^. Although the underlying relationship between aberrant splicing events and cancer is often not established, the potential exists to develop new diagnostic and therapeutic interventions when more insight is gained^9^. Therefore, a better understanding of the mechanisms of alternative splicing regulation and differences in splicing events between diseased and healthy tissues is considered crucial in cancer and other medical research^10^. By measuring a relative amount of distinct splice forms, it is possible to test whether a new splice form really constitutes an important fraction of a gene’s transcript. Several alternative splicing detection methods have been proposed for RNA sequencing (RNASeq)^11^ and microarray platforms^12,13^. Recent studies emphasize the complementary nature of RNASeq and microarrays; combined, both technologies have strengths which might overcome their reported weaknesses. The primary advantage of RNASeq is its potential to explore the entire diversity of a transcriptome while a microarray can be used to quantify lower abundance transcripts^14^. Since the RNASeq is unable to properly quantify low abundance transcripts and its competitive detection, the diversity of the resulting library is limited^15,16^. The limited diversity can be resolved by relying on microarray platforms such as the Affymetrix Exon ST arrays^12^ and the Human Transcriptome Arrays (HTA)^13^. Methods for alternative splicing detection using RNASeq include MATS, DEXSeq and Cufflinks^17–19^. However, these have shown to be insufficient^20^. Alternative splicing has been studied with microarray platforms using a variety of methods^21^. The Microarray Detection of Alternative Splicing (MiDAS) method employs gene-level normalized exon intensities in an ANOVA model based on a Splicing Index (SI)^12,22^. The SI method normalizes the exon level expression intensities by their corresponding gene level intensities, and compares the normalized intensities between sample groups. More recent methods for alternative splicing detection include the Robust Alternative Splicing Analysis for Human Transcriptome Arrays method (RASA)^23^, Integrated Gene and Exon Model of Splicing (iGEMS)^14^, AltAnalyze^24^ and the Random Effects model for the Identification of Differential Splicing (REIDS)^25^.

In this paper, we focus on HTA microarrays^26^. This array platform does not only assess each exon with more probes but also contains exon-exon junction probes that cover the region between two exons. In the current paper we extend the REIDS model^25^ to include junction information. The presence of junction probes can be beneficial for detecting alternatively spliced exons and for unravelling the composition of transcript isoforms. The analytical framework presented in this paper consists of two steps. In the first step the REIDS model was used for alternative splicing detection. In the second step the reliability of the junctions is evaluated to support (or not) alternatively spliced exons. The REIDS analytical framework model was compared with RASA, iGEMS, the Affymetrix TAC tool^12^ and the AltAnalyze software^24^.

## Methods and Materials

### Data

#### The HJAY Platform

The Affymetrix Human Transcriptome Array 1.0 (HJAY, HTA 1.0)^27^ is an expansion of the Human Exon array containing 10 probes per probe set. The HJAY array also contains exon-exon junctions which are supported by four to eight probes per junction. Junction probes were designed to reveal alternative splicing events and transcript isoforms. There are three types of junctions: a 5′ end junction, a 3′ end junction (located at the 5′ end and the 3′ end of the probe sets respectively) and an exclusion junction. The data we analysed in this paper is described in detail in^23^. Briefly, the data consists of two tissues, liver and muscle, each with three replicates. In total 33,516 genes were measured using 298,281 exons and 249,475 junctions, each represented by eight probes on average. The data is publicly available on the website http://igenomed.stanford.edu/~junhee/RASA/.

#### The HTA-2.0 Platform

The HTA-2.0 microarray is a more recent and updated version of the HJAY platform. The number of interrogated probe sets is increased to 1,048,904 and the number of junctions to 339,000. On avergae, exons are measured with 10 probes and junctions with four probes. The analyzed data set contains cell lines with A549 lung adenocarcinoma that were treated with either scrambled RNA or transfected with a siRNA that targets SRSF1. Each condition is represented by three independent subjects with three replicates per subjects, leading to nine samples per condition. The data is publicly available in the GEO database under the accession number GSE76902^28^.

### Detection of Alternative Spliced Exons: A Mixed Effects Model Approach

This section presents the extension of REIDS analytical framework to incorporate exon-exon junctions. In the first step the REIDS model was applied to find alternative spliced (AS) candidates as originally proposed^25^. In the second step a scheme was developed using junction formation to classify the identified exons from strongly confident to less confident candidates for alternative splicing.

#### The REIDS Model

The REIDS model^25^ formulates alternative splicing detection as a variance decomposition problem based on the assumption that between array variability of an alternatively spliced exon would be higher than the within array variability among the exons of the same transcript cluster (a gene). A non-alternatively spliced exon would have a between array variability that is at most the within array variability across all exons of the same transcript cluster. The two variance components, representing the between and within array variability, can be estimated using a gene specific mixed model given by

1 $$log\mathrm{2(}P{M}_{ijk})={p}_{j}+{c}_{i}+{b}_{ik}+{\varepsilon }_{ijk}.$$

The observed perfect match (PM) probe intensities are modelled in terms of the *j*th probe effect (*p*_*j*_, *j* = 1, …, *J*) and the overall effect of the *i*th array (*c*_*i*_, *i* = 1, …, *I*). Both *p*_*j*_ and *c*_*i*_ are considered to be fixed effects. The specific deviation of the *k*th exon from the overall gene effect is captured by the random effects, *b*_*ik*_, *k* = 1, …, *K*. The random effects *b*_*ik*_ ~ *N*(**0**, **D**_**k**_), are assumed to be independent of the background noise, ε_*ijk*_ ~ *N*(0, *σ*^2^), which captures the within array variability with *σ*^2^. Differential expression between the arrays is expected to be negated by incorporating the gene effect parameter *c*_*i*_. As a consequence, the exon specific parameters capture the deviation of a particular exon from its corresponding gene. A relatively small deviation implies that the exon is present in the sample while a large deviation indicates that the exon is likely to be absent.

#### Detection of Alternatively Spliced Exons

The advantage of a mixed model formulation for alternative splicing detection is the existence of a standard score to quantify the trade-off between signal and noise, the so-called intra-cluster correlation (ICC)^29^. From here onward we use the term ***exon score*** which is given, for the *k*th exon, by:

2 $${\rho }_{k}={\tau }_{k}^{2}/({\sigma }^{2}+{\tau }_{k}^{2}),$$

where *σ*^2^ is the same for all exons belonging to the same transcript cluster. A value of *ρ*_*k*_ > 0.5 indicates that the *k*th exon is likely to be alternatively spliced. Note that the threshold for the exon score could be adjusted to the relative amount of signal in the data. Given that the *k*th exon has been identified to have substantial between array variability, the estimated random effects *b*_*ik*_ can be used as ***array scores*** to identify arrays in which the alternatively spliced exon is expressed. Positive array scores denote an enrichment while negative values denote a depletion of the exon.

The REIDS method is applied to each gene to obtain exon and array scores after which the exons are prioritized according to their exon scores. Probe sets with exon scores greater than a pre-specified threshold are retained for further investigation. The differences in array scores between biological conditions or tissue types are tested using the t-test for independent groups or the paired t-test for paired data. A probe set is considered to be alternatively spliced if the difference between the biological conditions or tissue types is statistically significant after the Benjamini-Hochberg multiplicity correction^30^.

### Using Junction Information to Support Detection of Alternative Splicing Exons

In this section we explain how exon-exon junctions can either support or not support an exon to be allternatively spliced.

#### Motivation and Design

**Figure 1:**
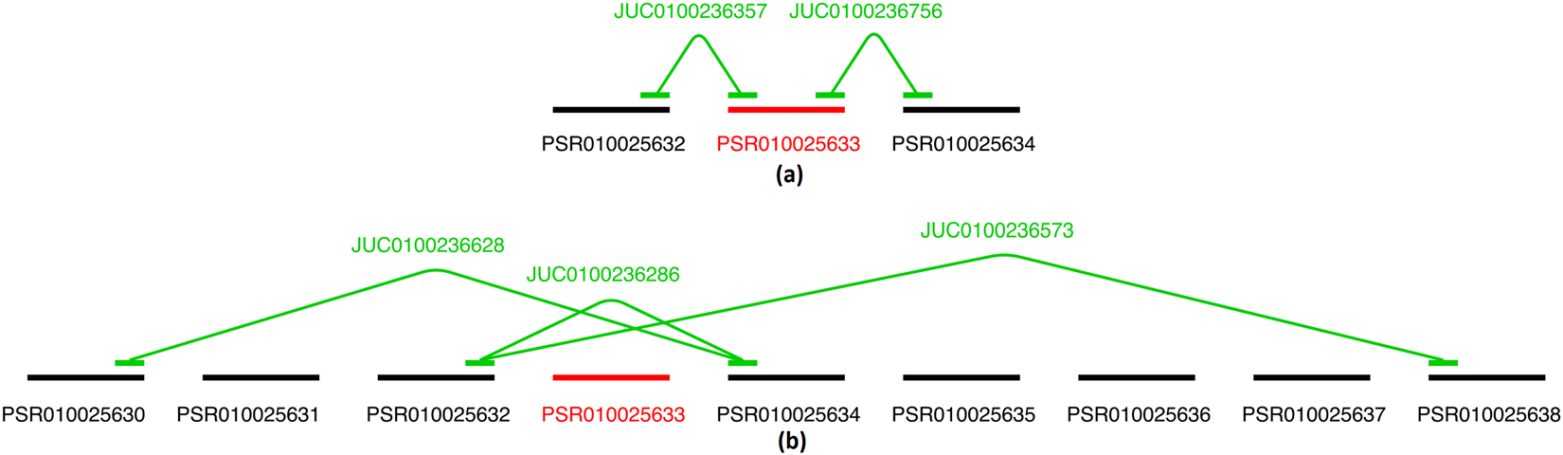
The junction design of probe set PSR010025633 of transcript cluster TC0102569 (CSDE1). Panel (a): The design of the 5′ and 3′ linking junctions of probe set PSR010025633. Panel (b): The design of the exclusion junctions of probe set PSR010025633.

We illustrate the exon-exon junctions by inspecting the design of probe set PSR010025633 of the transcript cluster TC0102569, annotated to the CSDE1 gene, on the HJAY microarray. This probe set has five annotated junctions: a 5′ end junction, a 3′ end junction and three exclusion junctions. Figure 1 illustrates the design of the probe set and its junctions.

**Figure 2:**
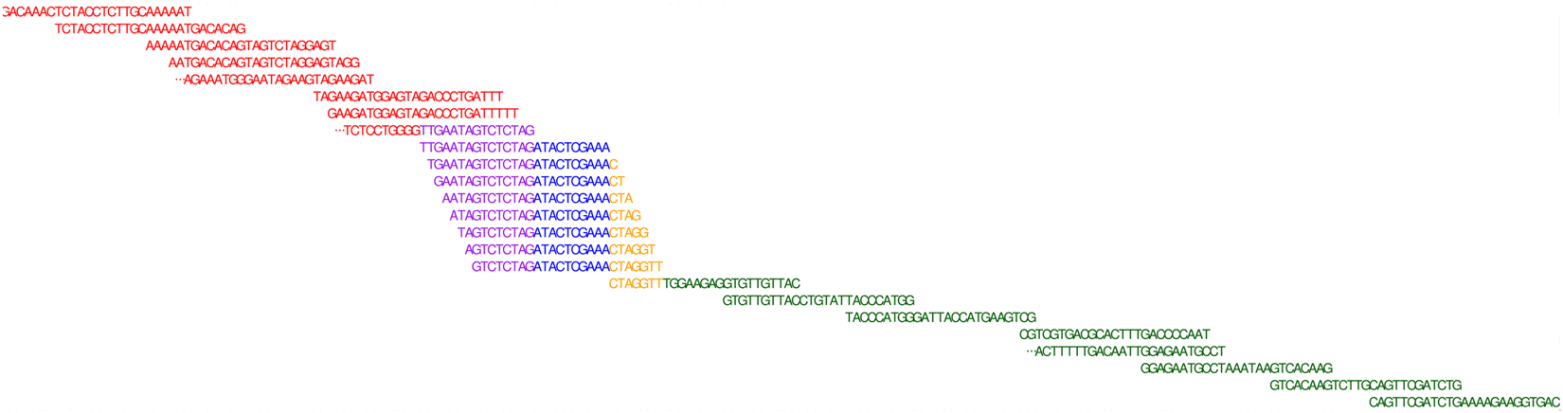
The probe sequences of PSR010025632 (red), JUC0100236357 (blue) and PSR010025633 (green) of gene TC0102569 (CSDE1) which are shown panel (a) in Fig. 1. The overlap between the junction and the 3′ end of the probe set on the left is shown in purple and yellow reflects the overlap between the junction the 5′ end of the probe set on the right.

The upper panel of Fig. 1 shows that junction JUC0100236357 is the 3′ end junction of PSR010025632 and the 5′ end junction of PSR010025633. This implies that the sequence of junction JUC0100236357 is constructed from the end (3′ end) of PSR010025632 and the start (5′ end) of PSR010025633. This is shown in Fig. 2. Notice how the sequence of JUC0100236357 overlaps with the sequence of its annotated probe sets. Similarly, JUC0100236756 is the 3′ end junction of PSR010025633 and the 5′ end junction of PSR010025634. This is shown in Supplementary Fig. 1.

We expect the presence of a junction only when both of its anchor points are present. This implies that JUC0100236357 will be quantified when both PSR010025632 and PSR010025633 are present in a transcript isoform without interruption. Similarly for junction JUC0100236756 and probe sets PSR010025633 and PSR010025634. Consequently, the presence or absence of the 5′ end and/or 3′ end junctions can indicate the presence or absence of a link between probe sets. As shown in Fig. 2, Junction JUC0100236357 has a matched sequence with the designed probes for both PSR010025632 and PSR010025633. This does not hold for all junctions. It is possible for a junction to have no 5′ end and/or 3′ end annotation or that the sequences of the junction do not match with the sequences of the probe sets. The latter implies that the junction might not quantify the same transcript as its adjacent probe sets.

The lower panel of Fig. 1 shows three exclusion junctions annotated to probe set PSR010025633: JUC0100236628, JUC0100236286 and JUC0100236573. These junctions are present when the exon is absent. Junction JUC0100236628 is annotated as the 3′ end junction of PSR010025630 and the 5′ end junction of PSR010025634. This entails that it will only be observed if probe sets PSR010025630 and PSR010025634 are included in the transcript isoform alongside each other and PSR010025631, PSR010025632 and PSR010025633 are excluded. Equivalently for junction JUC0100236286 which only skips over probe set PSR010025633 and junction JUC0100236573 which skips over all exons in between PSR010025632 and PSR010025638. In contrast with the 5′ end and 3′ end junctions, the presence of an exclusion junction informs us of the absence of the exon. Sequences of the exclusion junctions JUC0100236628 and JUC0100236573 are illustrated in Supplementary Figs 2 and 3 respectively.

#### Junction Assessment Procedure

First, we consider the 5′ end and 3′ end linking junctions. The linking junctions indicate whether the annotated anchor points are neighbouring in a transcript isoform. A linking junction reflects such a bond if the junction represents both exon probe sets simultaneously, i. e. if the junction probe set reflects the median profile of the exon probe sets. In order to validate this, a model is fitted on the junction probe set and it’s annotated exon probe sets. Since the pattern of splicing is more important than the expression values, the values are transformed to their corresponding ranks (*R*_*xk*_). Next, the ranks of the individual probes of the exon probe sets (i.e. not of the junction) are averaged and ranked anew to obtain a median profile representing both anchor points of the junction. Finally, two models were fitted on the averaged ranks of the probe sets and the ranks of the junction.

3 $$\begin{array}{rcl}M1:{R}_{xk} & = & probe\_se{t}_{k}+grou{p}_{x}+{\varepsilon }_{xk}\\ M\mathrm{2:}\,{R}_{xk} & = & probe\_se{t}_{k}+grou{p}_{x}+probe\_se{t}_{k}\times grou{p}_{x}+{\varepsilon }_{xk}\end{array}$$

with *k* = 1, …, *K* and *x* = 1, 2. Both models include a probe set effect and a group effect. The probe set effect is a factor with two levels either representing the profile of the anchor points or the junction. For the null model, M1, the junction has the same profile as the probe sets. Hence, no interaction effect is specified. The alternative model, M2, assumes a conflict between the junction and the probe sets, which is captured by an interaction effect. A significant interaction term implies that the junction is not an end product of the two probe sets.

**Figure 3:**
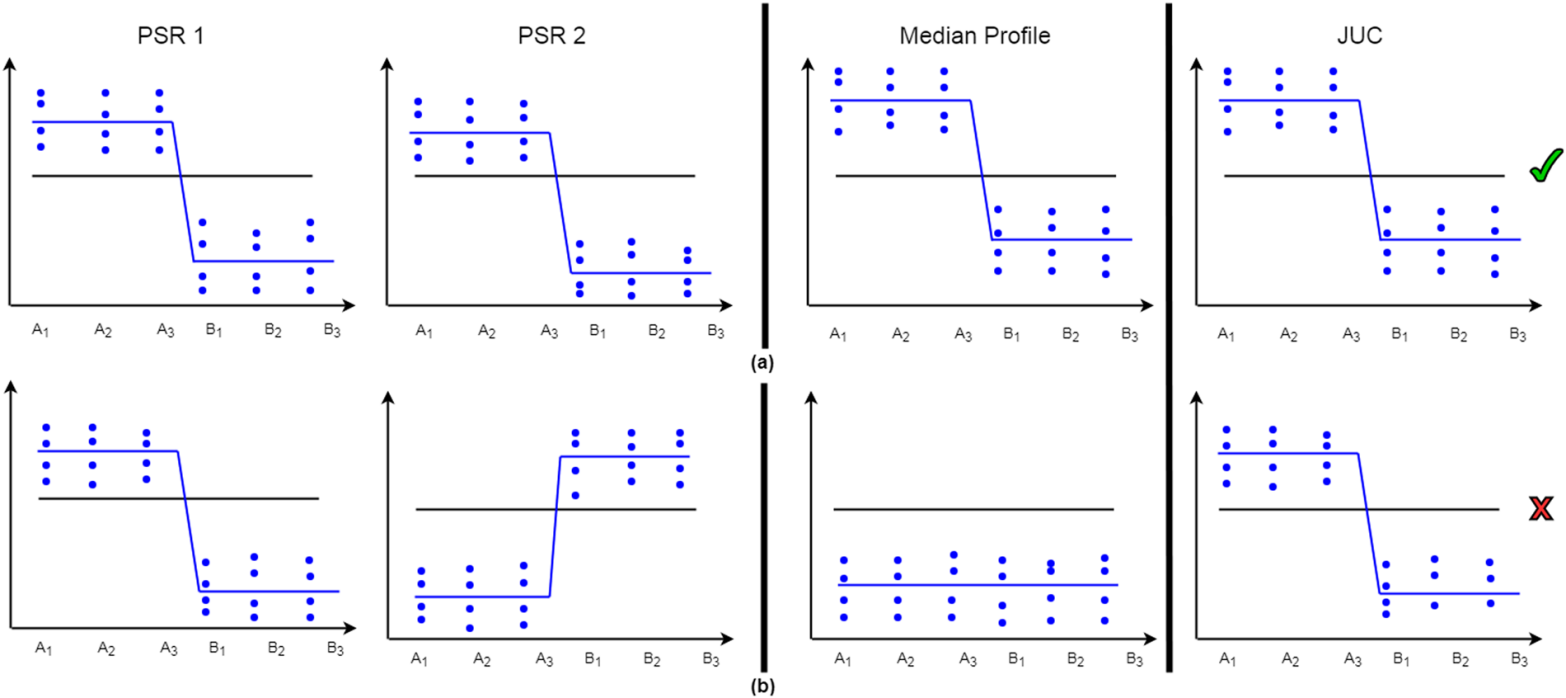
Alternatively spiced probe sets and annotated junction probe sets between condition A and condition B. The blue points represent the probes designed to measure a probe set. The blue lines represent the summarized value of a probe set and the black line shows the summarized value for all probe sets in transcript cluster (overall gene level). Panel (a): Evidence of linkage between PSR 1 and PSR 2 by the junction JUC, i.e. the junction reflects the median profile. Panel (b): Lack of evidence of linkage between PSR1 and PSR2, i.e. the junction (JUC) shows a different pattern.

Figure 3 illustrates two figurative scenarios of alternatively spliced exons between condition A (samples A_1_, A_2_ and A_3_) and a condition B (samples B_1_, B_2_ and B_3_). Panel (a) reflects a situation in which the median profile of the exon probe sets (PSR1 and PSR2) is reflected by the linking junction probe set (JUC). The values of the exon probe sets PSR1 and PSR2 are high in condition A and low in condition B. The median profile of PSR1 and PSR2 shows the expected the behaviour of the linking junction: high in condition A and low in condition B. The expression values of the junction reflect the same pattern as the prob sets, resulting in an insignificant interaction term. Therefore, the junction supports the link between PSR1 and PSR2. In the second scenario, presented in panel (b), the probe sets and the junction show a conflicting pattern. PSR1 is highly expressed in condition A and depleted in condition B while PSR2 shows a reversed pattern. The resulting median profile is straight line. However, the observed junction values show a deviating pattern. Consequently, the interaction term will be significant. A real data example and the conversion to ranks are illustrated in Supplementary Figs 4 and 5.

An exclusion junction is considered to be absent if it is not detected above background (DABG)^31^ level across all samples. This means that at least one of the skipped probe sets is considered present in an isoform and the junction does not support an alternative splicing event. Otherwise, the exclusion junction is present and support Otherwise, the junction is present and supports of the linking bond as well as of the AS candidacy of the excluded probe sets. The linking probe sets are considered to be neighbouring in at least one isoform, skipping all probe sets in between. For example in Fig. 1, if JUC0100236573 is deemed present by DABG analysis, probe sets PSR010025632 and PSR010025638 are neighbouring in at least one transcript isoform. However, if the junction is deemed absent by DABG analysis, then there is at least one exon probe set in between PSR010025632 and PSR010025638. DABG scores were calculated using the Affymetrix tool TAC. More information on exclusion junctions is given in Section 2.2 of the Supplementary Material.

The are four resulting categories of junction assessment of probe sets identified by REIDS: probe sets with linkages supported by all junctions, probe sets with linkages supported by at least one junction, not supported probe sets and probe sets without a 5′ end or 3′ end junction.

#### Alternative Splicing Types

Behind an AS identification, there is a specific AS type. This is determined by the location of the probe set in the transcript isoforms and the pattern of the neighbouring probe sets. The basic AS types are: a cassette exon, mutually exclusive exons, an alternative 5′ site, an alternative 3′ site, an alternative last, an alternative first and an intron retention. If information regarding the isoform composition of a transcript cluster is available, it can be deduced to which class an AS probe set belongs. In combination with the junction assessment procedure, the number of isoforms can be reduced if a reported linkage between probe sets is unsupported. A probe set is appointed the “complex event” class if the event does not belong to the arbitrary classes. Illustrations of AS events with examples of real life data are shown in Supplementary Section 2.3.

### Availability of data and materials

The HJAY data can be retrieved from the repository of the RASA paper^23^ and the HTA-2.0 date set from GEO database under the accession number GSE76902^28^. The code to process the data and perform the analysis is bundled into an R package, REIDS, available on CRAN.

## Results

The HJAY and HTA-2.0 data were preprocessed with the R package aroma.affymetrix^32^. An annotated.cdf file was created by merging the information from the provided.pgf and.clf files. In order to prepare the data for the REIDS model, the raw.CEL files were background corrected using the rma background correction, normalized with quantile-normalization and log2-transformed but without summarization. We illustrate the REIDS model with the HJAY data and benchmark the method with TAC and AltAnalyze based on the HTA-2.0 data.

### Illustration of the REIDS model on the HJAY platform

#### Step 1: Identification of Candidate AS Exons

**Figure 4:**
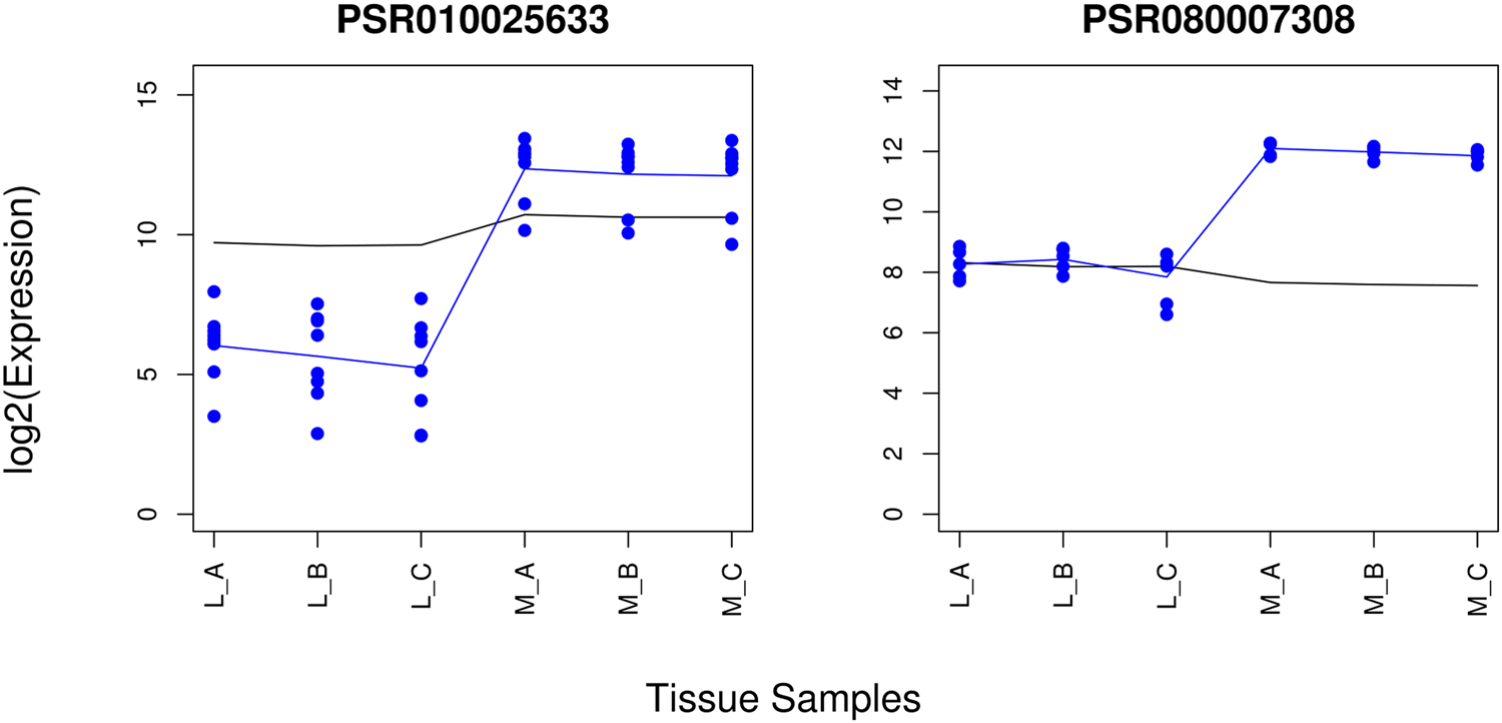
Alternative spliced candidates between the liver samples (L_A, L_B and L_C) and the muscle samples (M_A, M_B and M_C). The left panel shows probe set PSR010025633 of transcript TC0102569 and the right panel shows probe set PSR080007308 of transcript TC0800969. The black and blue lines indicate the mean profiles of the gene and exon level data respectively. The blue points show the probe level data.

A total of 33,516 genes were retained after pre-processing the data to probe level, 24,096 out of 33,516 genes had more than one probe set and were therefore selected for alternative splicing detection. Combined, these genes had 288,515 exon probe sets of which eventually 17,700 were identified by REIDS as candidates for alternative splicing. Figure 4 presents probe sets PSR010025633 of transcript TC0102569 and PSR080007308 of transcript TC0800969 which were found to be alternatively spliced between the liver (L) samples and the muscle samples (M) in the first step of the analysis.

**Figure 5:**
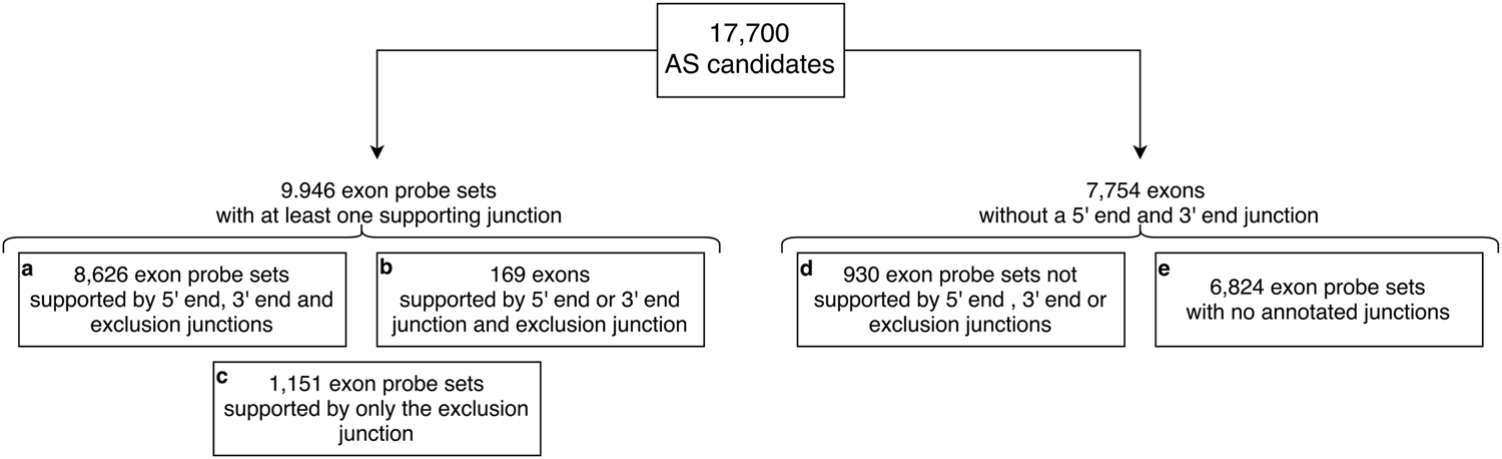
The distribution of the 17,700 exon probe sets that were identified as alternatively spliced by the REIDS model in the first step of the analytical framework. 8,626 probe sets were found to be AS and supported by all their annotated junctions. 9,946 probe sets were found to be AS with the support of at least one of their annotated junctions. There were 12,323 probe sets without junction support.

The identified probe sets were further analysed using their associated junction information. In the conservative mode we require the support of all annotated junctions. 8,626 out of the 17,700 exon probe sets were identified as alternative spliced candidates and were supported by all their annotated junctions. 9,946 out of the 17,700 exon probe sets were supported by at least one of their annotated junctions. Finally, 12,323 probe setshad no supporting junctions. Figure 5 provides an overview of the classification of the probe sets with and without supporting junctions.

### Step 2: Junction Based Classification

We show examples of exon probe sets which had support of all their annotated junctions to be alternatively spliced and those which had no junction support.

#### Alternative Splicing With Supporting Junctions

**Figure 6:**
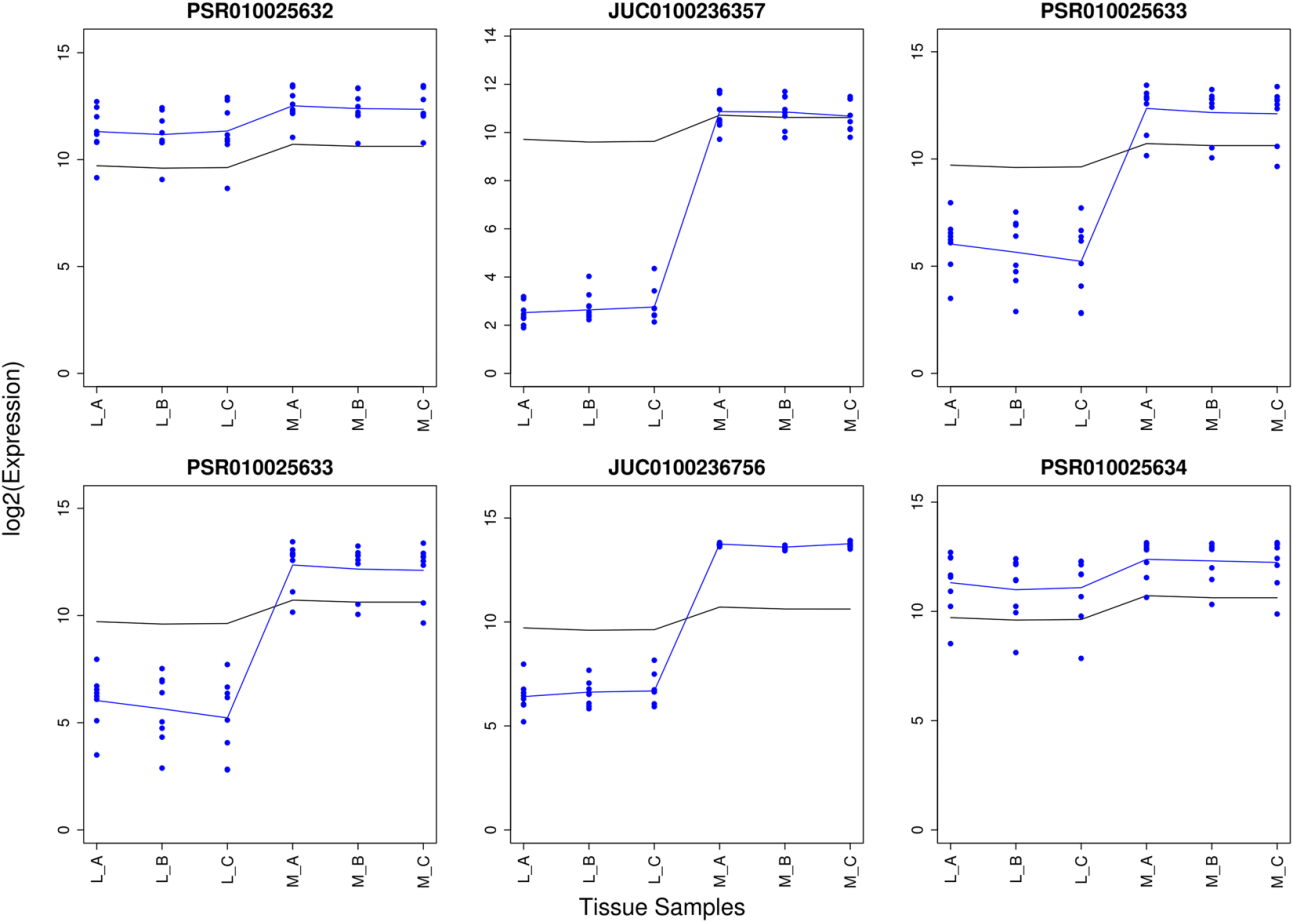
An example of a probe set supported by all its annotated junctions to be alternatively spliced between the liver (L) and muscle samples (M): PSR010025633 of transcript TC0102569. The junction architecture of PSR010025633 is shown in Fig. 1. The observed probe intensities of PSR010025632, JUC0100236357, PSR010025633, JUC0100236756 and PSR010025634 are presented relative to the summarized gene level values of TC0102569. The black and blue lines indicate the mean profiles of the gene and exon level data respectively. The blue points show the probe level data.

A top ranked probe set is PSR010025633 of transcript TC0102569 (Fig. 1) with an exon score of 0.99 and a p-value < 0.01 for the significance testing of its array scores between the liver and the muscle tissues. This exon belongs to category (a) in Fig. 5. Figure 6 shows the probe data and the summarized values of the probe set, its 5′ end and 3′ end junctions. Probe set PSR010025633 is depleted in the liver samples and enriched in the muscle samples. This pattern is also seen for the neighbouring probe sets PSR010025632 and PSR010025634 and the junction probe sets JUC0100236357 and JUC0100236756. Both linking junctions had a similar pattern as the probe set and support the exon to be alternatively spliced. The three exclusion junctions are presented in Supplementary Fig. 13 and revealed an opposite pattern. The presence of the exclusion junction confirms the absence of the probe set in at least some transcript isoforms. The alternative splicing detection of PSR010025633 was supported by all its annotated junctions.

Probe set PSR010025632 and PSR010025634 were both identified as constituitive probe sets. This implies that PSR010025633 is a cassette exon. Further, JUC0100236357 was not DABG in the liver samples. Therefore, a transcript which contains PSR010025632 and PSR010025633 simultaneously was not present in the liver samples. In the muscle samples, some transcripts include PSR010025633 while other transcripts exclude this probe set. Supplementary Fig. 14 shows the transcript composition of TC0102569 confirming the candidacy of probe set PSR010025633.

#### Alternative Splicing With At Least One Supporting Junction

**Figure 7:**
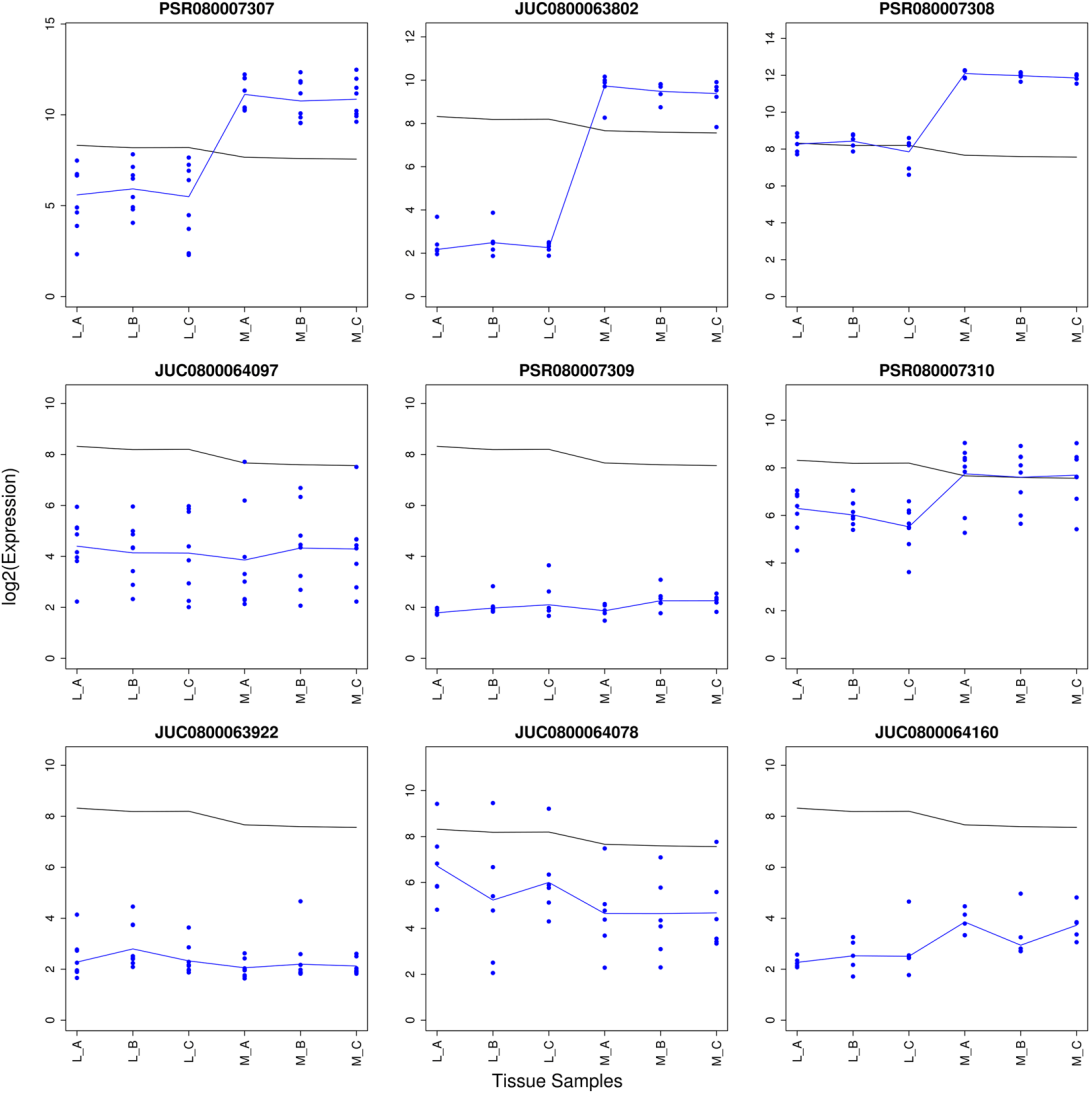
An example of a probse et not supported by all its annotated junctions to be alternatively spliced between the liver (L) and muscle samples (M): PSR080007308 of transcript TC0800969. The junction architecture of PSR080007308 is shown in Supplementary Fig. 15. The observed probe intensities of PSR080007307, JUC0800063802, PSR080007308, JUC0800064097, PSR080007309, PSR080007310, JUC0800063922, JUC0800064078 and JUC0800064160 are presented relative to the summarized gene level values of TC0800969. The black and blue lines indicate the mean profiles of the gene and exon level data respectively. The blue points show the probe level data.

Probe set PSR080007308 is part of the TC0800969 transcript annotated to the ASPH gene. It has a 5′ end, a 3′ end and three exclusion junctions. The junction architecture is shown in Supplementary Fig. 15. Figure 7 illustrates the probe set and its annotated junctions. In the upper row, 3′ end junction JUC0800063802 reflects the pattern of its anchor points PSR080007308 and PSR080007307. The junction supports a linkage between the two probe sets. However, in the middle row, the 5′ end junction JUC0800064097 shows probe is depleted for all samples with a DABG p-values < 0.05. A possible reason could be the pattern of its other anchor point. However, no 3′ end annotation was found for this junction. The junction was annotated as an exclusion junction for probe sets PSR080007309 and PSR080007310. The consistent presence of either of these probe sets can disrupt the junction sequence leading to its depletion. Probe set PSR080007309 was excluded for all samples but the values of PSR080007310 were indicative of an inclusion in at least a transcript isoform.

**Figure 8:**
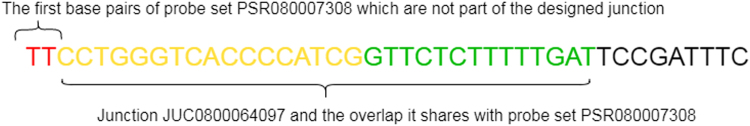
The cDNA sequence of probe set PSR080007308 of transcript cluster TC0800969 as found in the Ensembl database.

The observed pattern of the junction might also arise from the design of its probes. The designed sequences of the probes of the 5′ end junction JUC0800064097 and probe set PSR080007308 are shown in Supplementary Fig. 16. Both probe sets show overlapping sequences. In contrary, the whole sequence of probe set PSR080007308 has a different beginning in the ensemble genome browser as presented in Fig. 8.

The sequence of PSR080007308 starts with the base pairs “TT” which is not part of the sequence of the designed junction. The sequence of the junction in the whole genome actually matches with an intron adjacent to PSR080007308. Consequently, it will not be present in the cDNA sequence and thus cannot be measured with a microarray. It is difficult to assess whether the pattern of the junction is due to this apparent annotation to an intron or not.

The exclusion junctions JUC0800064160 and JUC0800064078 do not have two anchor probe sets, but they had low expression levels. The exclusion junction JUC0800063922 also had low expression levels for all samples. This is supported by the DABG values which confirms the presence of probe set PSR080007308. The transcript composition of PSR080007308 is shown in Supplementary Fig. 17 and shows a cassette exon event for PSR080007308. Since the probe set is supported by its 3′ end junction and one exclusion junction, it could be considered to be alternatively spliced. Section 3.3 of the Supplementary Material shows an additional examples in Supplementary Figs 18 and 19.

#### Alternative Splicing Without Supporting Junctions

In this section we present exon probe set PSR010016411 which was not supported by its annotated exclusion junction to be alternatively spliced This example belongs to category d in Fig. 5.

**Figure 9:**
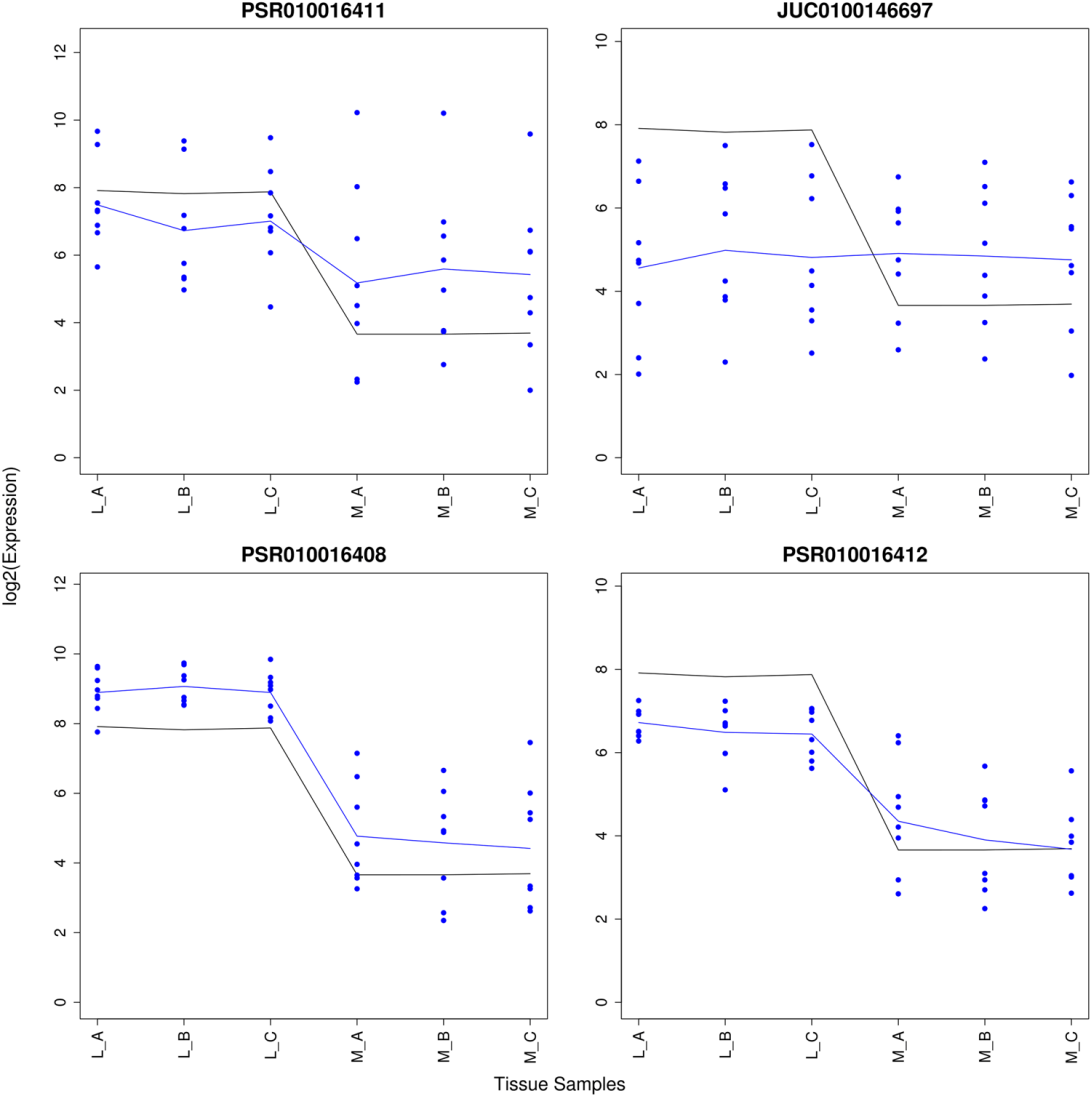
An example of a probe set that was not supported by its annotated junctions to be alternatively spliced between the liver (L) and muscle samples (M): PSR010016411 of transcript TC0101665 (KMO) The observed probe intensities of PSR010016411, JUC0100146697, PSR010016408 and PSR010016412 are presented relative to the summarized gene level values of TC0102569. The black and blue lines indicate the mean profiles of the gene and exon level data respectively. The blue dots show the probe level data.

Probe set PSR010016411 is part of transcript TC0101665 referring to the KMO gene. Figure 9 presents the probe set, the exclusion junction JUC0100146697 and the anchor probe sets of the junction: PSR010016408 and PSR010016412. The junction is not DABG indicating that PSR010016408 and PSR010016412 are not neighbouring probe sets in the isoform composition in the tissues. The sequence can be interrupted by the presence of either PSR010016408, PSR010016409 or PSR010016410. It is certain that at least one of these probe sets interrupts the sequence but without further junction annotation, it is unknown which probe set is continuously included in the isoforms.

The transcript composition is shown in Supplementary Fig. 20. The ensemble data base indicates that only the four isoforms containing PSR010016408 are protein coding. Therefore, PSR010016408 can be considered a constituitive probe set. Complementary examples of difficulties when designing junctions are shown in Section 3.4 of the Supplementary Material. Example of a probe set supported by all linking junction is provided in Section 3.5 of the Supplementary Material. A study of transcript cluster TC1601187 is presented in Section 3.6 of the Supplementary Material.

#### Validation of the REIDS model

This section assess the performance of the REIDS model on a list of 373 exons that were identified by the RASA method and validated as alternatively spliced between liver and muscle tissues by RT-PCR^23^. A total of 49,014 probe sets were identified as alternatively spliced by the RASA method. As shown in Fig. 5, REIDS identifies 17,700 candidacies for alternative splicing in the first step of the analytical framework. 8,626 out of the 17,700 probe sets supported by all their annotated junctions while 9,946 probe sets were supported by at least one of their annotated junctions. The RASA and REIDS model shared 8,273 probe sets in common after step one but this reduced to 4,973 and 5,736 probe sets depending on whether the probe sets were supported by all their annotated junction or by at least one of their annotated junctions. Table 1 shows the comparison of the REIDS analytical framework with AltAnalyze and iGEMS based on the list of probe sets identified by RASA and validated by either RNA sequencing or RT-PCR.

**Table 1:** The true positive rates (% recall) for REIDS, AltAnalyze and iGEMS for the 373 RT-PCR validated alternatively spliced exons.

	REIDSraw	REIDScon	REIDSlib	AltAnalyze	iGEMS
AS	287 (77%)	210 (56%)	244 (65%)	102 (27%)	230 (62%)
N-AS	86 (23)%	163 (44%)	129 (35%)	98 (26%)	143 (38%)

REIDSraw refers to the results obtained from the first step of the REIDS analytical framework. REIDScon refers to the analysis based on the support of all the annotated junction for each probe set, while REIDSlib refers to the analysis based on the support of at least one of the annotated junction for each probe set.

The REIDS model identified 287 (77%) probe sets in common with the list without using junction probe sets, 210 (56%) were supported by all their annotated junctions and 244 (65%) were supported by at least one of their annotated junctions. This shows that the REIDS analytical framework detected between 65–77% of the validated exon probe sets. The true positive rates for AltAnalyze and iGEMS were 27% and 62%, respectively. Supplementary Section 4 illustrates differences in identifications between REIDS and RASA on the HJAY microarray platform.

#### Comparison with Other Software

**Figure 10:**
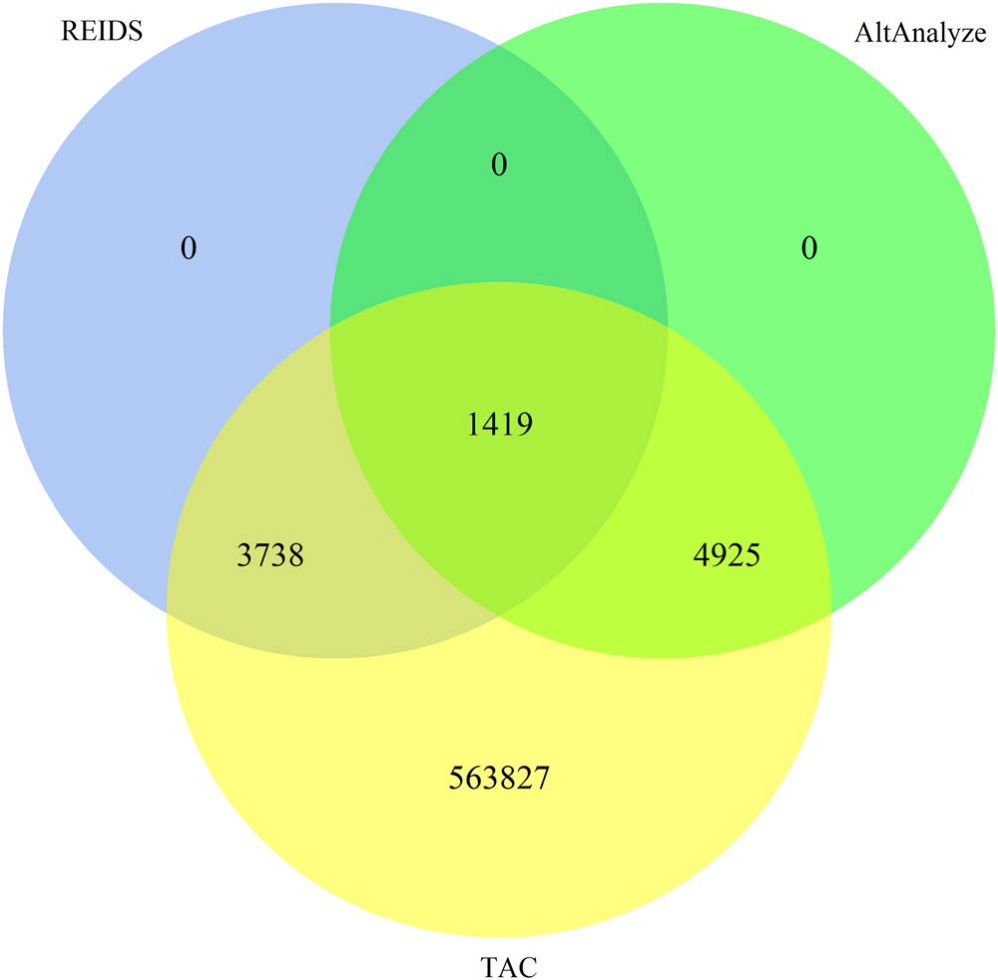
A Venn diagram showing the number of alternatively spliced probe sets identified by the REIDS method, AltAnalyze and TAC.

The REIDS analytical framework was further compared with the Affymetrix TAC tool and AltAnalyze software using a more recent HTA-2.0 platform. The data set contains A549 lung adenocarcinoma cell lines which were treated with either scrambled RNA (SCR) or transfected with a siRNA that targets SRSF1. The TAC tool identifies alternatively spliced exons based on a splicing index (SI)^12^ while AltAnalyze relies on the ASPIRE algorithm^24^. The Venn diagram in Fig. 10 compares the number of probe sets identified by the three methods.

All probe sets identified by REIDS were also identified by TAC and AltAnalyze. TAC identifies the largest number of probe sets but most of the probe sets had little variation in their intensities with less than 50% signal (exon scores <0.5) between arrays or biological samples. This means that the TAC method is prone to identifying probe sets which do not show variation across the conditions and it is therefore likely to be more prone to false positives. Table 2 shows the top 10 probe sets identified by the REIDS method. Section 3.8 in the Supplementary Material shows similar tables for the top 10 probe set obtained by AltAnalyze and TAC.

**Table 2:** Comparison of the ranks of the top 10 identified AS probe sets by the REIDS model in TAC and AltAnalyze.

Rank in REIDS	Gene	Probe Set	Exon Score	P-Val	Event Type	Rank in AltAnalyze	Rank in TAC
1	TC12001539	PSR12020244	0.93	<0.01	Complex Event	77	294
2	TC12001539	PSR12020245	0.93	<0.01	Alternative First	199	2,064
3	TC14000916	PSR14009987	0.92	<0.01	Alternative Last	29	241
4	TC12000010	PSR12000150	0.92	<0.01	Complex Event	5	17
5	TC01000469	PSR01007294	0.91	<0.01	Alternative Last	645	6,564
6	TC06001355	PSR06016183	0.90	<0.01	Alternative Last	—	1
7	TC06001387	PSR06016266	0.90	<0.01	Alternative Last	—	1,437
8	TC12001539	PSR12020242	0.89	<0.01	Alternative Last	745	2,782
9	TC02000874	PSR02012906	0.88	<0.01	Alternative First	—	4,949
10	TC05001924	PSR05026599	0.88	<0.01	Cassette Exon	88	110

**Figure 11:**
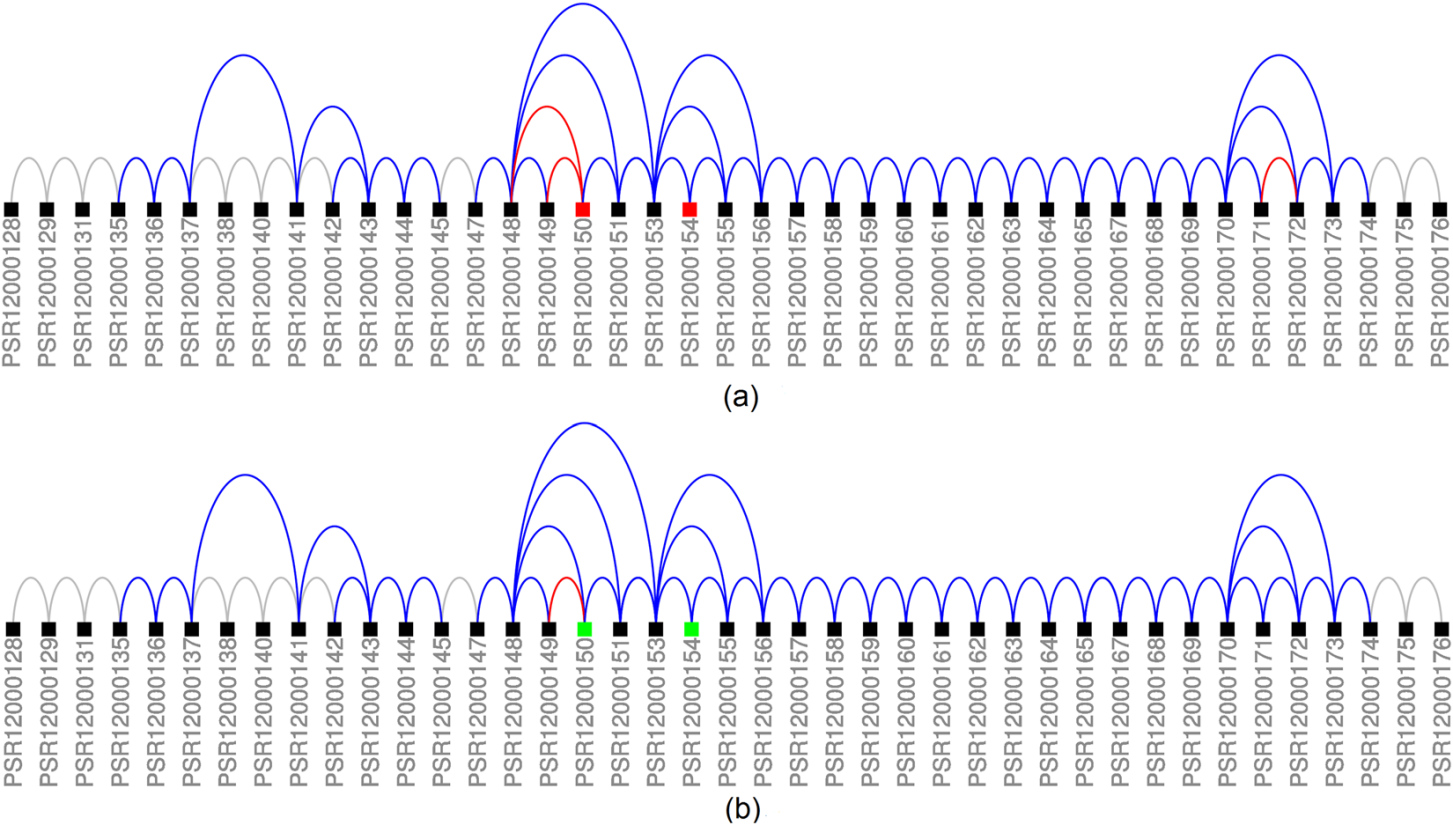
Illustration of transcript cluster TC12000010 (WNK1). Black probe sets were identified as constituitive while coloured probe sets were identified as alternatively spliced. Green colour indicates an enrichment of a probe set while red colour denotes a depletion. Junctions which are DABG are shown in blue while depleted junctions are again shown in red. Panel (a) shows the probe sets for the SCR samples and panel (b) for the siRNA samples.

An example of a probe set that was highly ranked by all the three methods is probe set PSR12000150 of transcript cluster TC12000010 (WNK1 gene). The probe set has two annotated 5′ end, one 3′ end and two exclusion junctions. Figure 11 illustrates the isoform transcriptions of TC12000010. Black probe sets were identified as constituitive while coloured probe sets were identified as alternatively spliced. Green colour indicates an enrichment of a probe set and red colour denotes depletion. Junction which were DABG are shown in blue while depleted junctions are again shown in red. PSR17017166 was identified as a cassette exon which was enriched in the SCR samples (upper panel) and depleted in the siRNA samples (lower panel). Supplementary Section 3.7 shows the junction architecture and expression levels for probe set PSR12000150.

**Figure 12:**
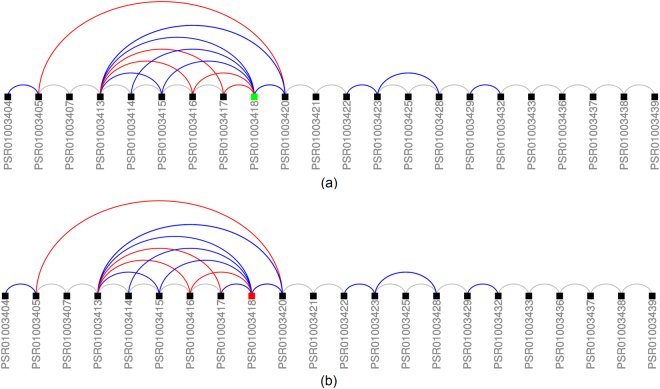
Illustration of transcript cluster TC01000205 (SZRD1). Black probe sets were identified as constituitive while coloured probe sets were identified as alternatively spliced. Green colour indicates an enrichment of a probe set while red colour denotes a depletion. Junctions which are DABG are shown in blue while depleted junctions are again shown in red. Panel (a) shows the probe sets for the SCR samples and panel (b) for the siRNA samples.

Probe set PSR01003418 was ranked higher by REIDS (32) compared to AltAnalyze (6,119) and TAC (15,074). The probe set was part of transcript cluster TC01000205 (SZRD1 gene) and was identified as an complex event. Figure 12 illustrates the composition of transcript cluster TC01000205. The isoform which has probe sets PSR01003417 and PSR01003417 as neighbouring probe sets seems more prominent in the siRNA samples.

**Figure 13:**
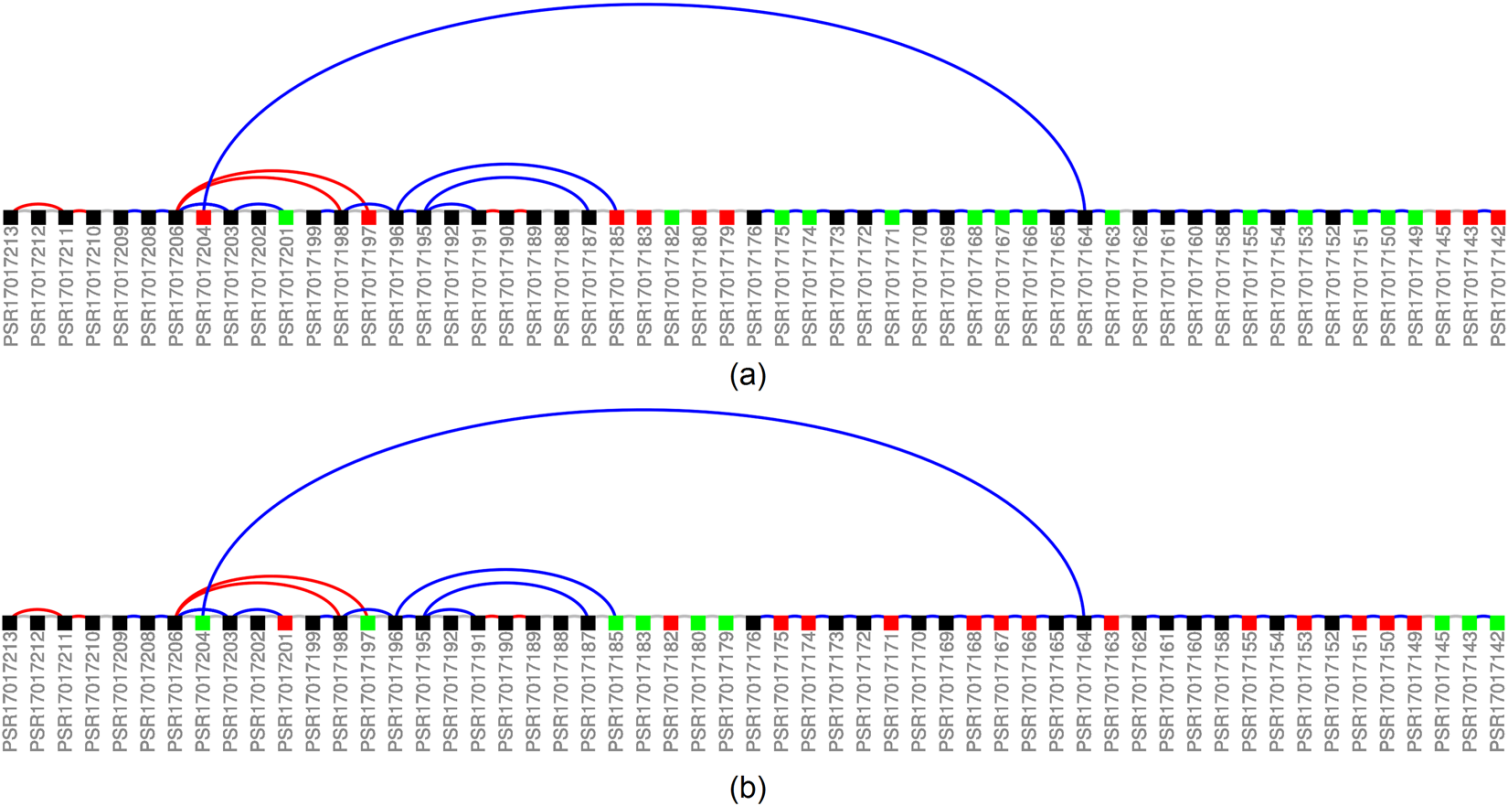
Illustration of transcript TC17001298 (SPAG5). Black probe sets were identified as constituitive while coloured probe sets were identified as alternatively spliced. Green colour indicates an enrichment of a probe set while red colour denotes a depletion. Junctions which are DABG are shown in blue while depleted junctions are again shown in red. Panel (a) shows the probe sets for the SCR samples and panel (b) for the siRNA samples.

Finally, we present probe set PSR17017175 of transcript cluster TC17001298 (SPAG5 gene) which was ranked higher by TAC (187) and AltAnalyze (178) than by the REIDS method (4545). Figure 13 shows the gene model. PSR17017175 was not one of the top ranked probe sets by the REIDS method since the probe set levels reflect the gene expression levels as shown in Supplementary Section 3.7.

## Discussion

The identification of alternative splicing events is important drug targeting. Numerous studies have shown that aberrant splice variants are involved in cancer development, neurodegenerative diseases, autosomal recessive diseases and more. Therefore, the identification of alternatively spliced probe set regions is an important step forward. The HTA microarray platform probe sets target exons as well as exon-exon junctions. An alternatively used probe set region supported by at least one of its linking junctions is less likely to be false positive compared to a probe set without junction support.

We proposed an extended REIDS analytical framework to incorporate information from junction probe sets. The REIDS analytical frame work outperforms AltAnalyze and iGEMS based on validated alternatively splcied exons. Although, only 65–77% of the validated alternatively spliced exons were detected by the REIDS method, this is partly because of the exon score filtering step. This filtering step assumed that transcript clusters with low variation between exons are not likely to be alternatively spliced. The REIDS method was further compared with TAC and AltAnalyze based on a more recent HTA-2.0 microarray platform. The REIDS method performs as good AltAnalyze, and is less prone to false positives than TAC.

The presence of exon-exon junctions provides valuable information on reducing the false positive rates in the detection of alternative spliced events using a microarray platform. However, caution is needed. In some cases behaviour of junction probe sets cannot be wholly captured by a test statistic or numeric metric. A careful inspection of the different junctions and their annotated transcript could provide useful information on the composition of the transcript isoforms. This means in addition to the numerical algorithms, a careful qualitative inspection of the different junctions and annotations is important in minimising false positive rates. In conclusion, the REIDS method incorporates exon-exon junctions to robustly identified alternatively spliced events. We have shown how junction information can be used to make a more adequate decision about alternatively spliced events. Like any other numerical method, the REIDS analytical framework has its drawback because of the pre-specified threshold required for exon score. A careful consideration of an appropriate threshold should be considered before applying the proposed REIDS analytical framework^33^.

## Electronic supplementary material

Supplementary Material

## References

[1] Mironov AA, Fickett JW, Gelfand MS (1999). Frequent alternative splicing of human genes. Genome Res..

[2] Wang ET (2008). Alternative isoform regulation in human tissue transcriptomes. Nat..

[3] Pan Q, Shai O, Lee LJ, Frey BJ, Blencowe BJ (2008). Deep surveying of alternative splicing complexity in the human transcriptome by high-throughput sequencing. Nat. Genet..

[4] Chen, L. *Handbook of Statistical Bioinformatics*, chap. Statistical and Computational Studies on Alternative Splicing, 31–53 (Springer, Berlin, 2011).

[5] Black DL (2003). Mechanisms of alternative pre-messenger rna splicing. Annu. Rev. Biochem..

[6] Epstein, C. J. Developmental genetics. *Exp*. **42** (1986). 10.1007/BF01941286 3021509

[7] Crayton ME, Powell BC, Vision TJ, Giddings MC (2006). Tracking the evolution of alternatively spliced exons within the dscam family. BMC Evol. Biol..

[8] Fan W, Khalid N, Hallahan AR, Olson J, Zhao L (2006). A statistical method for prediciting splice variants between two groups of samples using genechip expression array data. Theor. Biol. Med. Model..

[9] Gardina, P. *et al*. Alternative splicing and differential gene expression in colon cancer detected by a whole genome exon array. *BMC Genomics* **7**, 325, http://www.biomedcentral.com/1471-2164/7/325, 10.1186/1471-2164-7-325 (2006). 10.1186/1471-2164-7-325 PMC1769375 17192196

[10] Bisognin A (2014). An integrative framework identifies alternative splicing events in colorectal cancer development. Mol. Oncol..

[11] Wang B (2014). Similarity network fusion for aggregrating data types on a genomic scale. Nat..

[12] Affymetrix. Alternative transcript analysis methods for exon arrays. *Affymetrix Whitepaper*, http://www.affymetrix.com/support/technical/whitepapers.affx (2005).

[13] Affymetrix. Genechip human transcriptome array 2.0 data sheet, http://tools.thermofisher.com/content/sfs/brochures/hta_array_2_0_datasheet.pdfdatasheet.pdf (2013).

[14] Sood, S. *et al*. igems: an integrated model for identification of alternative exon usage event. *Nucleic Acids Res*. **44**, e109, http://nar.oxfordjournals.org/content/early/2016/04/19/nar.gkw263.abstract (2016). 10.1093/nar/gkw263 PMC4914109 27095197

[15] Lei R, Ye K, Gu Z, Sun Xa (2015). Diminishing returns in next-generation sequencing (ngs) transcriptome data. Gene.

[16] Mele M (2015). The human transcriptome across tissues and individuals. Sci..

[17] Shen S (2012). Mats: a bayesian framework for flexible detection of differential alternative splicing from rna-seq data. Nucleic Acids Res..

[18] Anders S, Reyes A, Huber Wa (2012). Detecting differential usage of exons from rna-seq data. Genome Res..

[19] Trapnell C (2010). Transcript assembly and quantification by rna-seq reveals unannotated transcripts and isoform switching during cell differentiation. Nat. Biotechnol..

[20] Liu R, Loraine AE, Dickerson JA (2014). Comparisons of computational methods for differential alternative splicing detection using rna-seq in plant systems. BMC Bioinforma..

[21] Lee, C. & Roy, M. Analysis of alternative splicing with microarrays: successes and challenges. *Genome Biol*. **5**, 10.1186/gb-2004-5-7-231 (2004). 10.1186/gb-2004-5-7-231 PMC463277 15239822

[22] Clark, T. *et al*. Discovery of tissue-specific exons using comprehensive human exon microarrays. *Genome Biol*. **8**, R64, http://genomebiology.com/2007/8/4/R64, 10.1186/gb-2007-8-4-r64 (2007). 10.1186/gb-2007-8-4-r64 PMC1896007 17456239

[23] Seok, J., Xu, W., Davis, R. W. & Wenzhong, X. Rasa: Robust alternative splicing analysis for human transcriptome arrays. *Sci. Reports* **5** (2015). 10.1038/srep11917 PMC4491729 26145443

[24] Emig D (2010). Altanalyze and domaingraph: analyzing and visualizing exon expression data. Nucleic Acids Res..

[25] Van Moerbeke M (2017). A random effectiveects model for the identifcation of differenterential splicing (reids) using exon and hta arrays. BMC Bioinforma..

[26] Xu W (2011). Human transcriptome array for high-throughput clinical studies. PNAS.

[27] Affymetrix. Genechip human transcriptome array 2.0 data sheet URL Available at, http://tools.thermofisher.com/content/sfs/brochures/EMI07313-2_DS_Clariom-D_solutions_HMR.pdf#/legacy=affymetrix.com (2013).

[28] Kasim JP (2016). EventPointer: an effective identification of alternative splicing events using junction arrays. BMC Genomics.

[29] Verbeke, G. Longitudinal Data Analysis. In Verbeke, G. & Molenberghs, G. (eds) *Linear mixed models in practice: A SAS-oriented approach*, no. 126 in Lecture Notes in Statistics, chap. **3**, 63–153 (Springer-Verlag, New York, 1997).

[30] Benjamini Y, Hochberg Y (1995). Controlling the false discovery rate: a practical and powerful approach to multiple testing. J. Royal Stat. Soc. B Met..

[31] Affymetrix. Identifying and validating alternative splicing events. *Affymetrix Tech. Notes*, https://tools.thermofisher.com/content/sfs/brochures/id_altsplicingevents_technote.pdf (2007).

[32] Bengtsson H, Irizarry R, Carvalho B, Speed T (2008). Estimation and assessment of raw copy numbers at the single locus level. Bioinforma..

[33] Kasim, A. *et al*. Informative or noninformative calls for gene expression: A latent variable approach. *Stat. Appl. Genet. Mol. Biol*. **9**(1), Article 4 (2010). 10.2202/1544-6115.1460 20196754

