# Supplementary material for: The Usage of Exon-Exon Splice Junctions for the Detection of Alternative Splicing using the REIDS model
Source: Sci Rep. 2018 May 29;8:8331. doi: 10.1038/s41598-018-26695-9 (PMC5974242; doi:10.1038/s41598-018-26695-9)
Supplement: Supplementary file 1 — Supplementary Material [file 41598_2018_26695_MOESM1_ESM.pdf]

The Usage of Exon-Exon Splice Junctions for the Detection of Alternative Splicing using the REIDS model

Marijke Van Moerbeke, Adetayo Kasim and Ziv Shkedy

1 Introduction

This appendix consists of additional clarifications and examples which are mentioned in the main manuscript. Section 2 provides an extra example of the sequence design of the junctions and Alternative splicing types. In Section 3 we present additional material for the Results Section of the main manuscript.

2 Methods and Materials

2.1 Motivation and Design

Figure S1 shows the overlapping sequence of junction JUC0100236756 with probe sets PSR010025633 and PSR010025634.

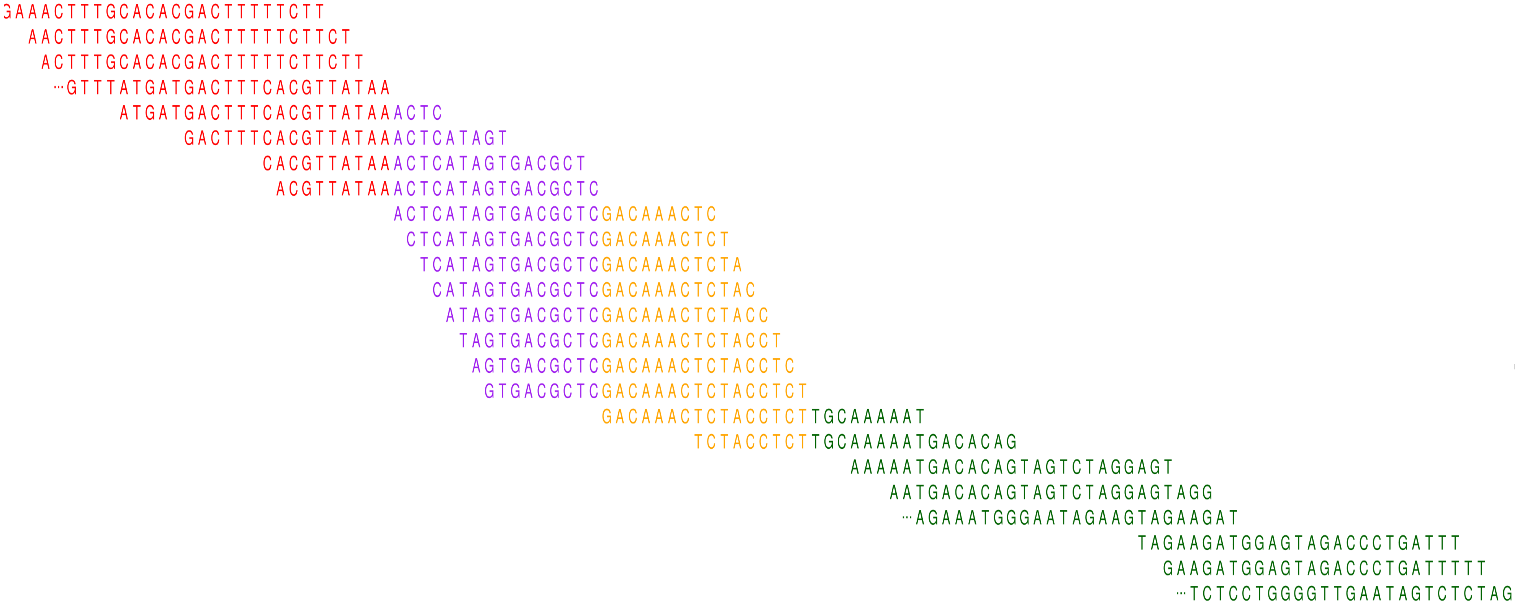

Figure S 1: The probe sequences of PSR010025633 (red), JUC0100236756 (blue) and PSR010025634 (green) of gene TC0102569 (CSDE1) which are shown panel (a) in Figure 1. The overlap between the junction and the 3′ end of the probe set on the left is shown in purple and yellow reflects the overlap between the junction the 5′ end of the probe set on the right.

Figure S2 and Figure S3 illustrate the overlapping sequences of junctions JUC0100236628 and JUC0100236573 which are annotated exclusion junction of probe set PSR010025633. Junction JUC0100236628 is a 3′ end junction of probe set PSR010025630 and a 5′ end junction of PSR010025634. Junction JUC0100236573 is a 3′ end junction of probe set PSR010025632 and a 5′ end junction of PSR010025638.

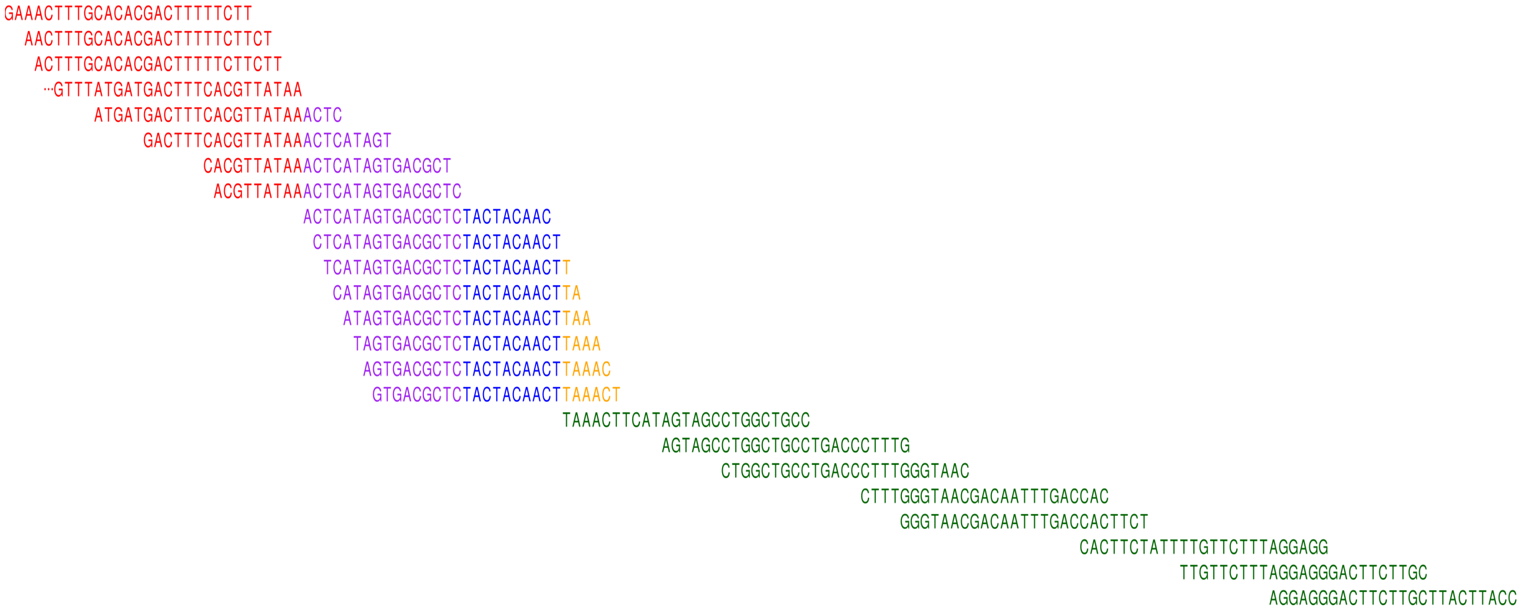

Figure S 2: The probe sequences of PSR010025630 (red), JUC0100236628 (blue) and PSR010025634 (green) of gene TC0102569 (CSDE1) which are shown panel (b) in Figure 1. The overlap between the junction and the 3′ end of the probe set on the left is shown in purple and yellow reflects the overlap between the junction the 5′ end of the probe set on the right.

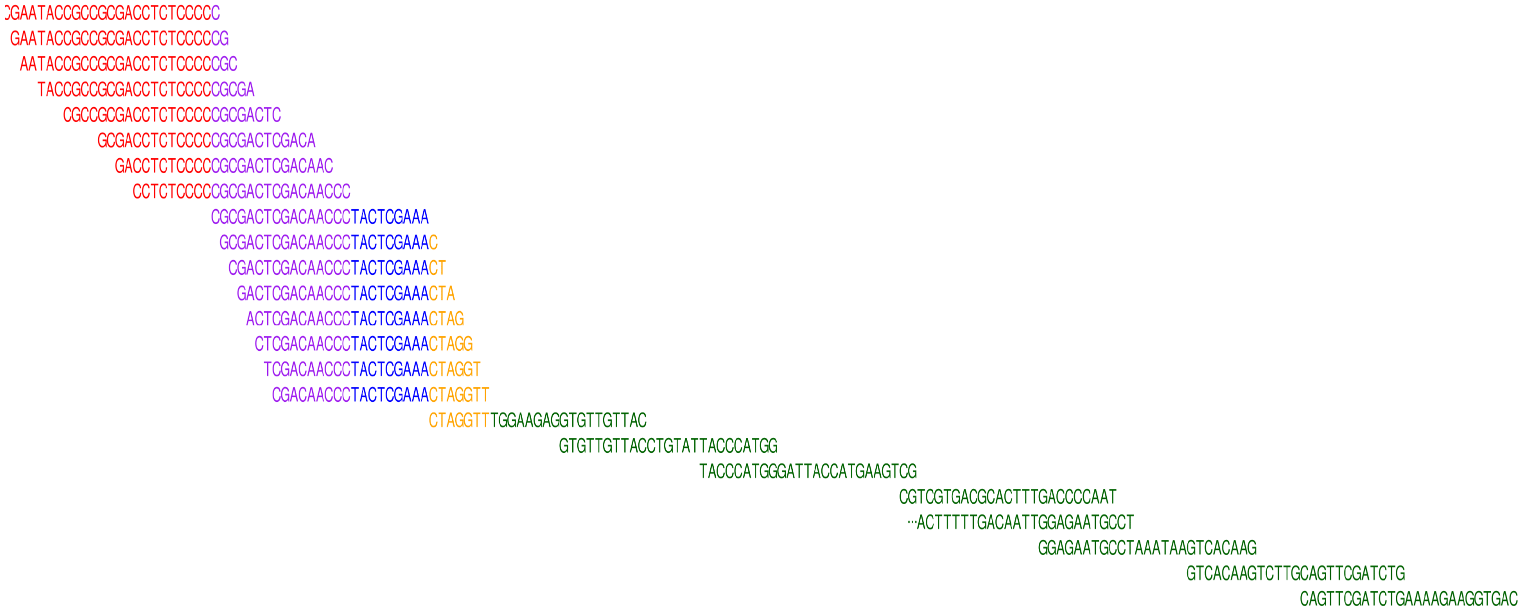

Figure S 3: The probe sequences of PSR010025632 (red), JUC0100236573 (blue) and PSR010025638 (green) of gene TC0102569 (CSDE1) which are shown panel (b) in Figure 1. The overlap between the junction and the 3′ end of the probe set on the left is shown in purple and yellow reflects the overlap between the junction the 5′ end of the probe set on the right.

2.2 Junction Assessment Procedure

Figure S4 and Figure S5 illustrate the conversion between the observed probe level values and ranking process as explained in the main manuscript in order to asses the support of 5′ and 3′ end junctions. The ranking of the probe sets retains the

pattern of alternative splicing. Figure S4 shows a junction which is the end result of the exon and thus the junction is confirming the alternative splicing. By consequence, the interaction term in the introduced models is not expected to be significant. Alternatively, Figure S5 shows an example where the pattern differs between the exons and its junction. The interaction term was deemed significant.

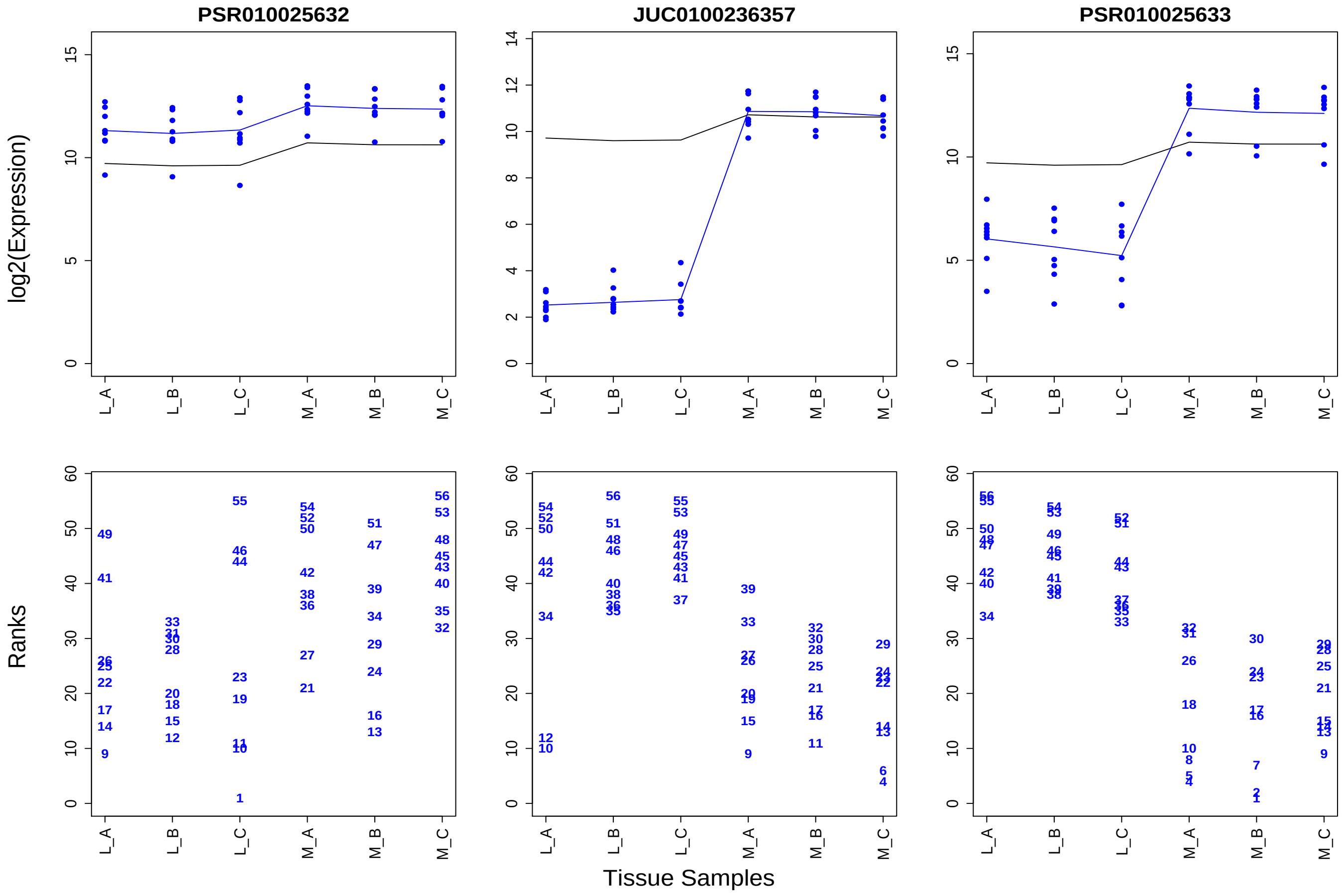

Figure S 4: Upper panel: the observed probe values of PSR010025632, JUC0100236357, PSR010025633 of transcript cluster TC0102569 (CSDE1). The black and blue lines indicate the mean profiles of the gene and exon level data respectively. The blue points show the probe level data. Lower panel: converted ranks of PSR010025632, JUC0100236357, PSR010025633.

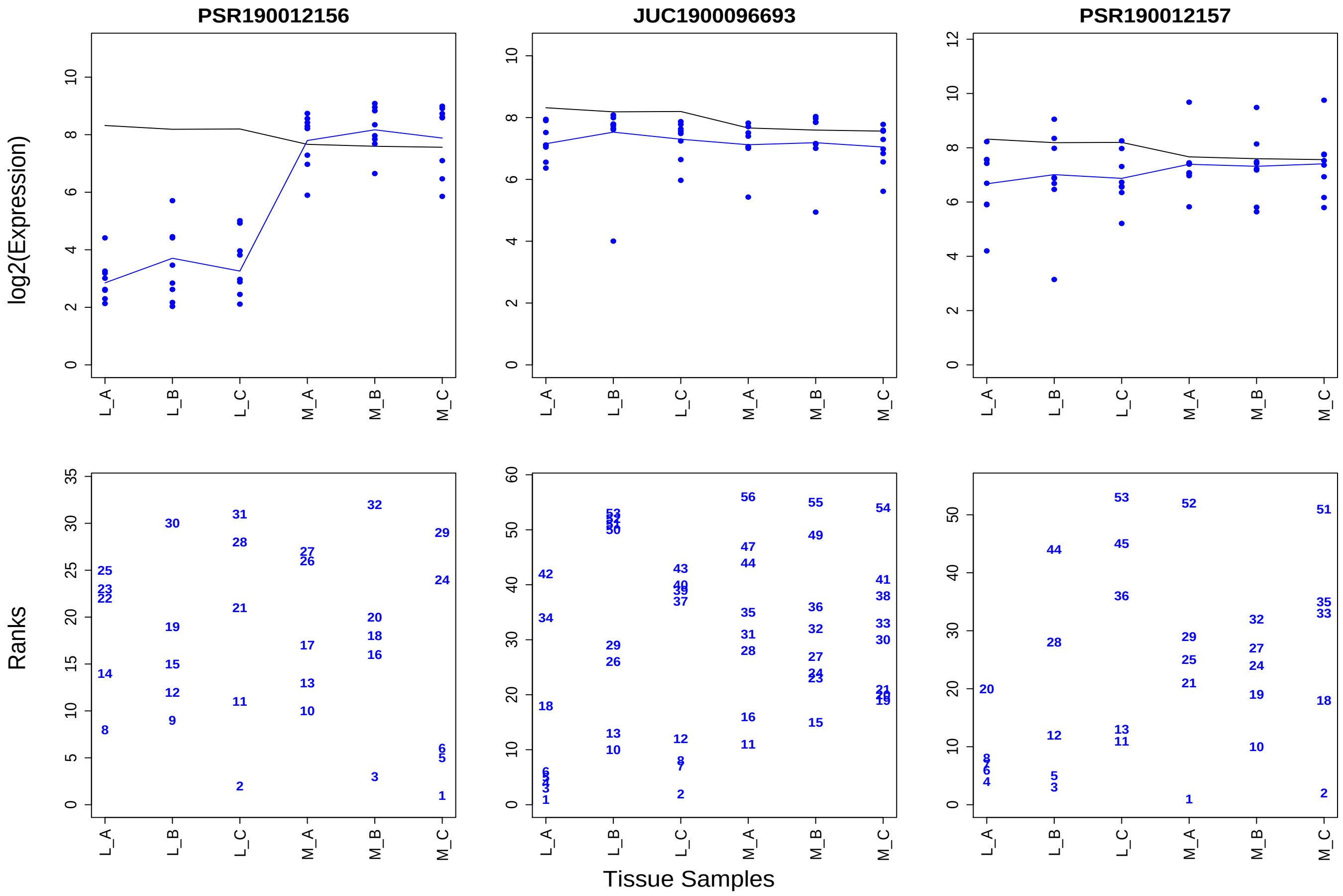

Figure S 5: Upper panel: the observed probe values of PSR190012156, JUC1900096693 and PSR190012157 of transcript cluster TC1901243 (DNAJB1) . The black and blue lines indicate the mean profiles of the gene and exon level data respectively. The blue points show the probe level data. Lower panel: converted ranks of PSR190012156, JUC1900096693 and PSR190012157.

After assessing the supporting information of the 5′ end and 3′ end junctions, information of the exclusion junctions can be incorporated as well. As stated in the main manuscript, we asses whether an exclusion junction is present by testing whether the probe values are DABG [1] for all samples. Figure S6 shows a probe set which is supported by both the 5′end and 3′end linking junctions as well as its annotated exclusion junction. It concerns probe set PSR010004149 of transcript TC0100415 (MACF1 gene) which has a 5′end junction JUC0100034464 and a 3′end junction JUC0100031528 duplicating the alternative splicing behaviour of the probe set. The exclusion junction JUC0100032584 is present and shows that PSR010004148 and PSR010004150 are neighbouring probe sets in at least a percentage of the transcript isoforms.

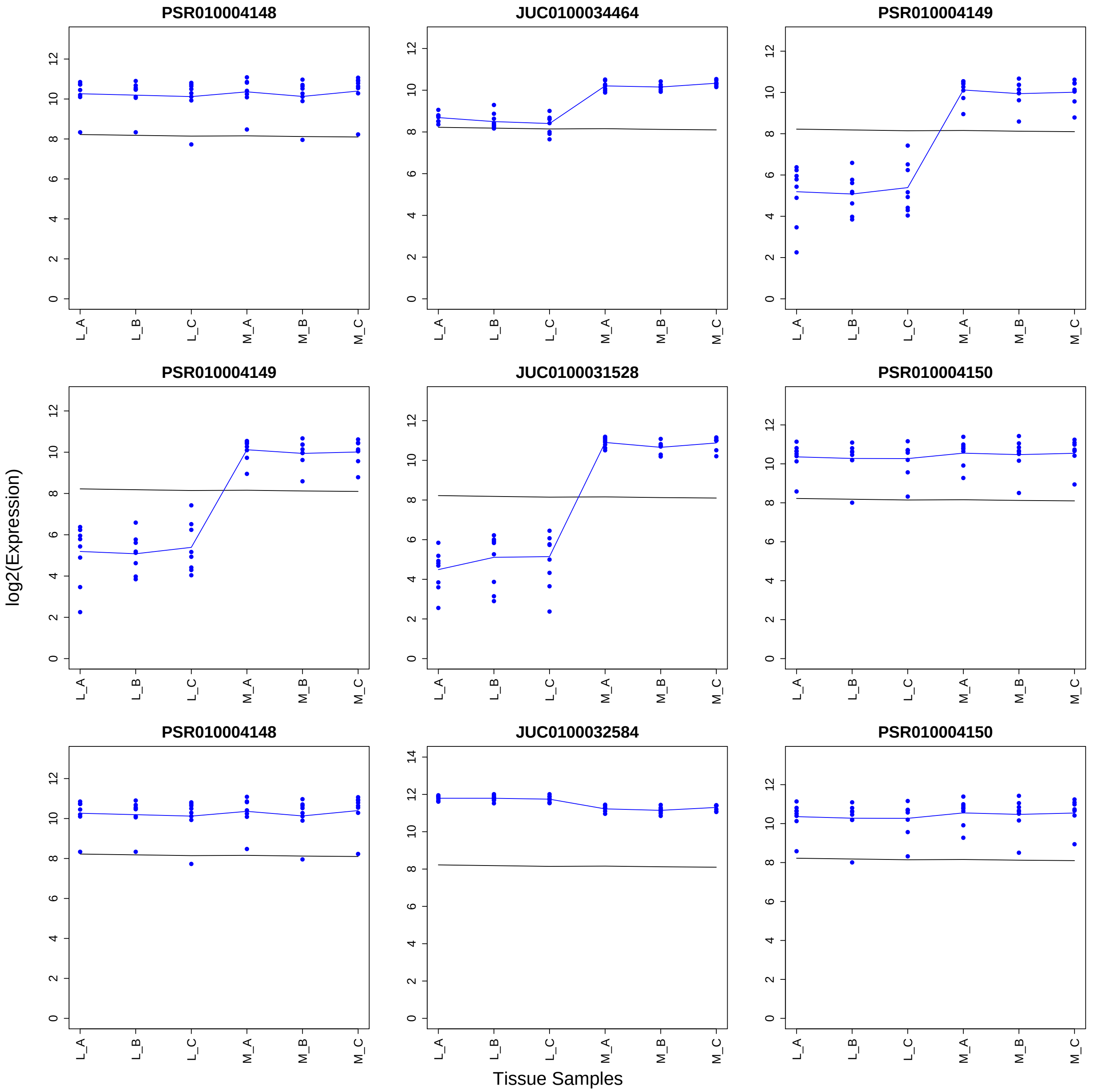

Figure S 6: The linking and exclusion junctions of probe set PSR010004149 of transcript cluster TC0100415 (MACF1). The observed probe values of PSR010004148, JUC0100034464, PSR010004149, JUC0100031528, PSR010004150 and junction JUC0100032584 relative the to the summarized gene level values of TC0100415. The black and blue lines indicate the mean profiles of the gene and exon level data respectively. The blue points show the probe level data.

Figure S7 shows probe set PSR210000091 of transcript TC2100019 (USP25 gene) which is supported by its 3′ end linking junction JUC2100000575 and its annotated exclusion junction JUC2100000476. The 5′ end junction JUC2100000433 does not replicate the behaviour of probe set PSR210000091 causing the exon to be excluded from the conservative mode of the REIDS model. However, the exclusion junction shows that PSR210000079 and PSR210000094 are neighbouring probe

sets in at least a percentage of the transcript isoforms.

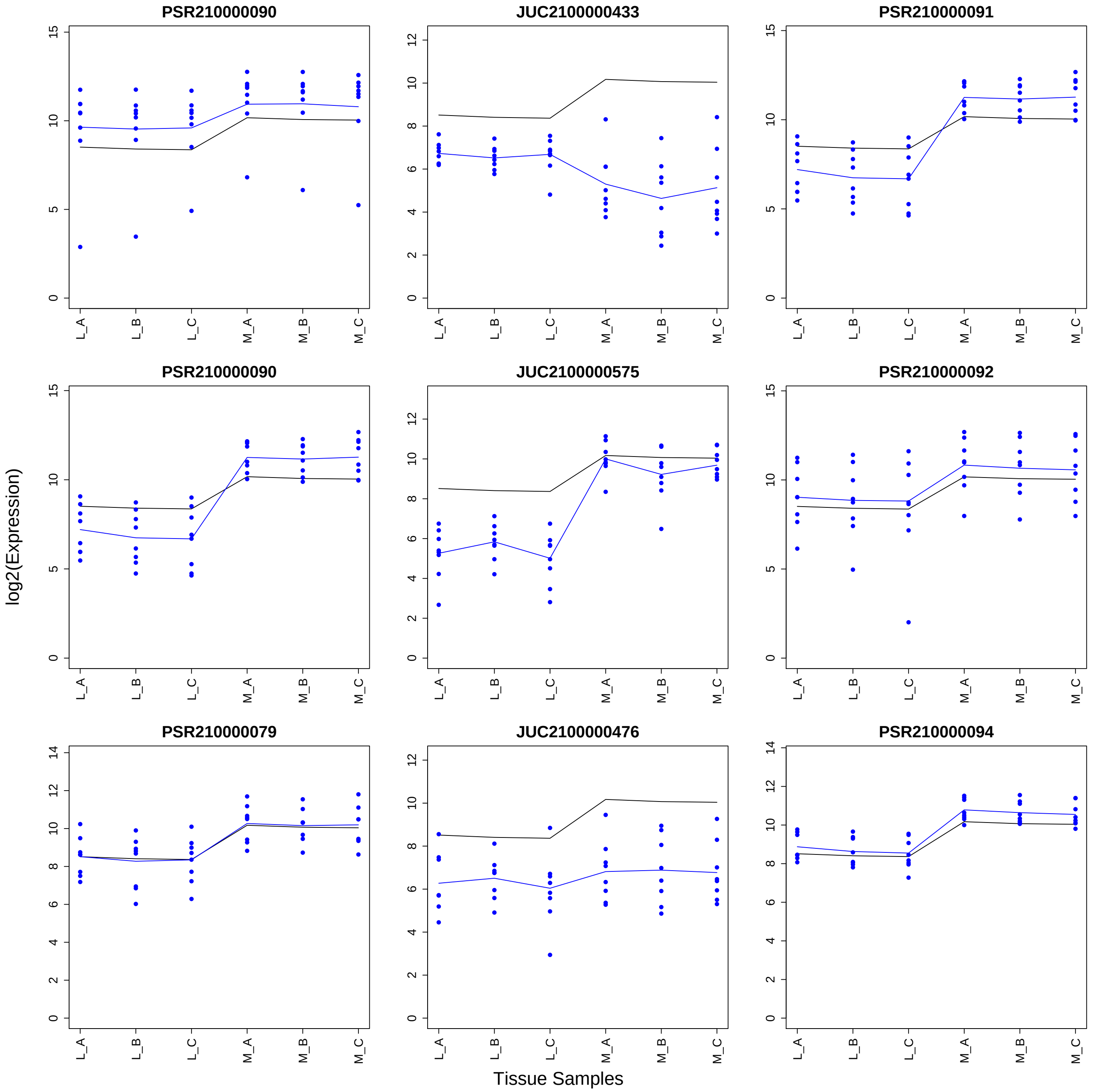

Figure S 7: The linking and exclusion junctions of probe set PSR210000091 of transcript cluster TC2100019 (USP25). The observed probe values of PSR210000090, JUC2100000433, PSR210000091, JUC2100000575, PSR210000092, PSR210000079, JUC2100000476 and PSR210000094 relative the to the summarized gene level values of TC2100019. The black and blue lines indicate the mean profiles of the gene and exon level data respectively.

2.3 Alternative Splicing Types

Behind an AS identification, there is a specific AS type. This is determined by the location of the probe set in the transcript isoforms and the behaviour of the neighbouring probe sets. The basic AS types are: a cassette exon, mutually

exclusive exons, an alternative 5′ site, an alternative 3′ site, an alternative last, an alternative first and an intron retention. Figure S8 illustrates the several alternatively splicing mechanisms [2].

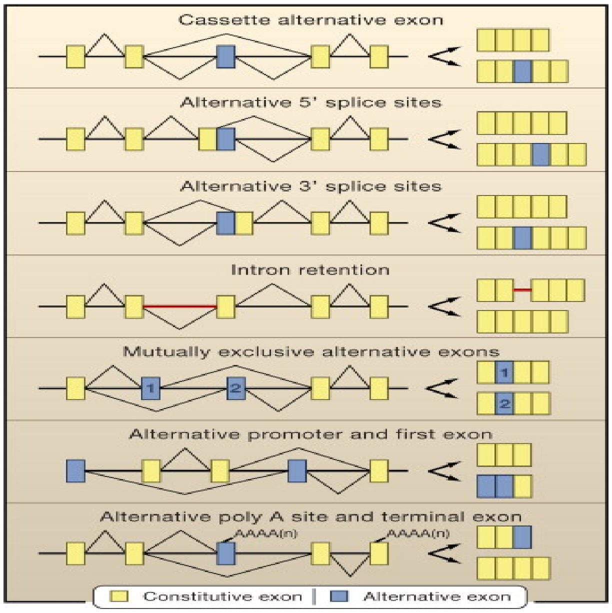

Figure S 8: Illustrations of the several known mechanisms behind an alternative splicing event.

If information regarding the isoform composition of a transcript cluster is available, it can be deduced to which class an AS probe set belongs. The output of the REIDS algorithm is a file which describes the derived event behind every positive AS identification. Table S1 shows examples of identification by the REIDS model for the HTA-2.0 data set.

Table S 1: Examples of different alternative splicing types identified by the REIDS model in combination with transcript isoform information.

TC ID	PSR ID	AS type
TC01001299	PSR01020107	Alternative Last
TC04001300	PSR04017716	Alternative First
TC04001332	PSR04018315	Intron Retention
TC01000863	PSR01013744	Cassette Exon

Figures S9 to S12 show the trannscript isoform collection of the examples presented in Table S1.

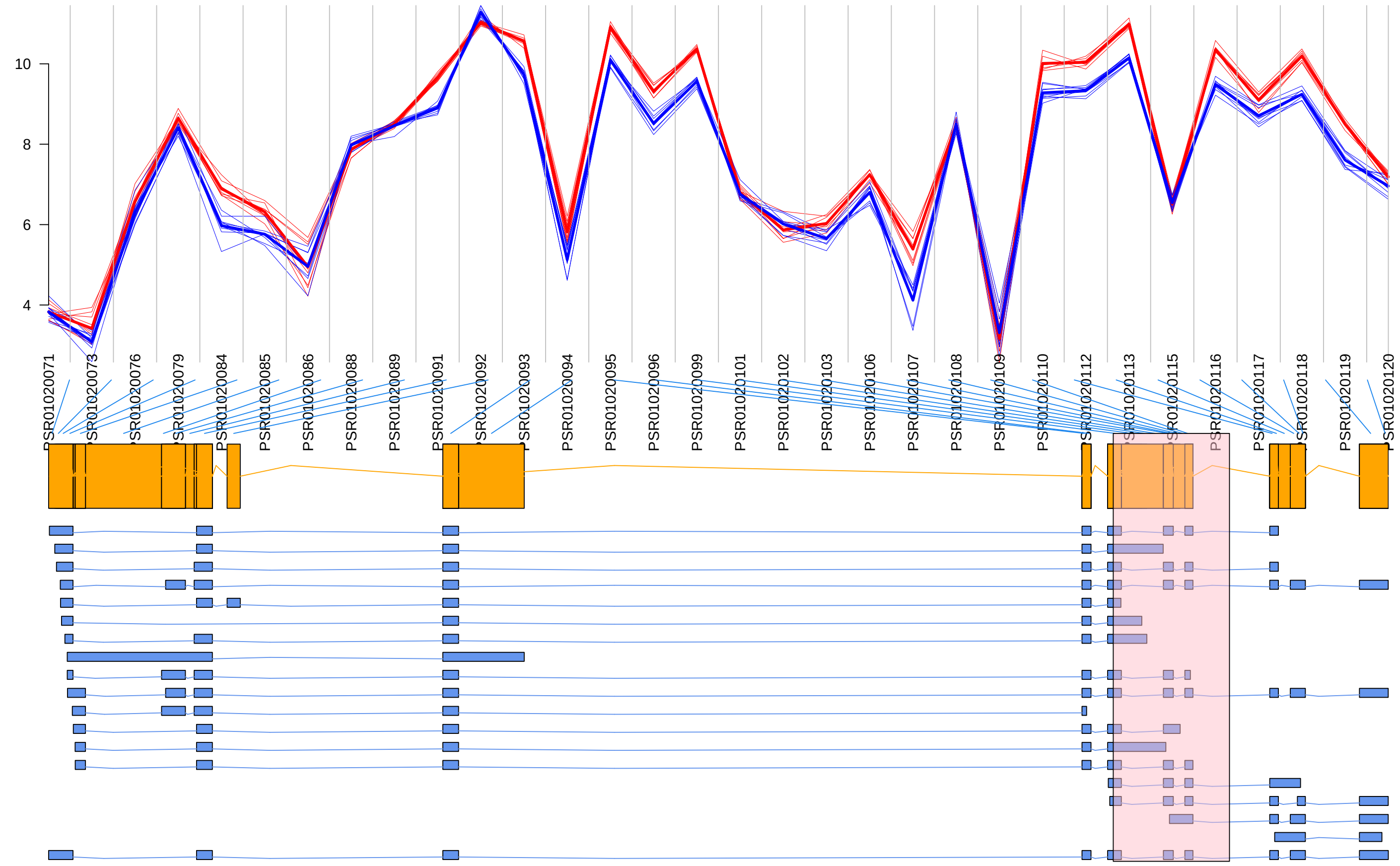

Figure S 9: The isoform composition of transcript cluster TC01001299 (FDPS). Probe set PSR01020107 is highlighted as an alternative last exon. The red lines represent the probe set expression levels of the SCR samples. The blue lines show the probe set expression levels for the siRNA samples.

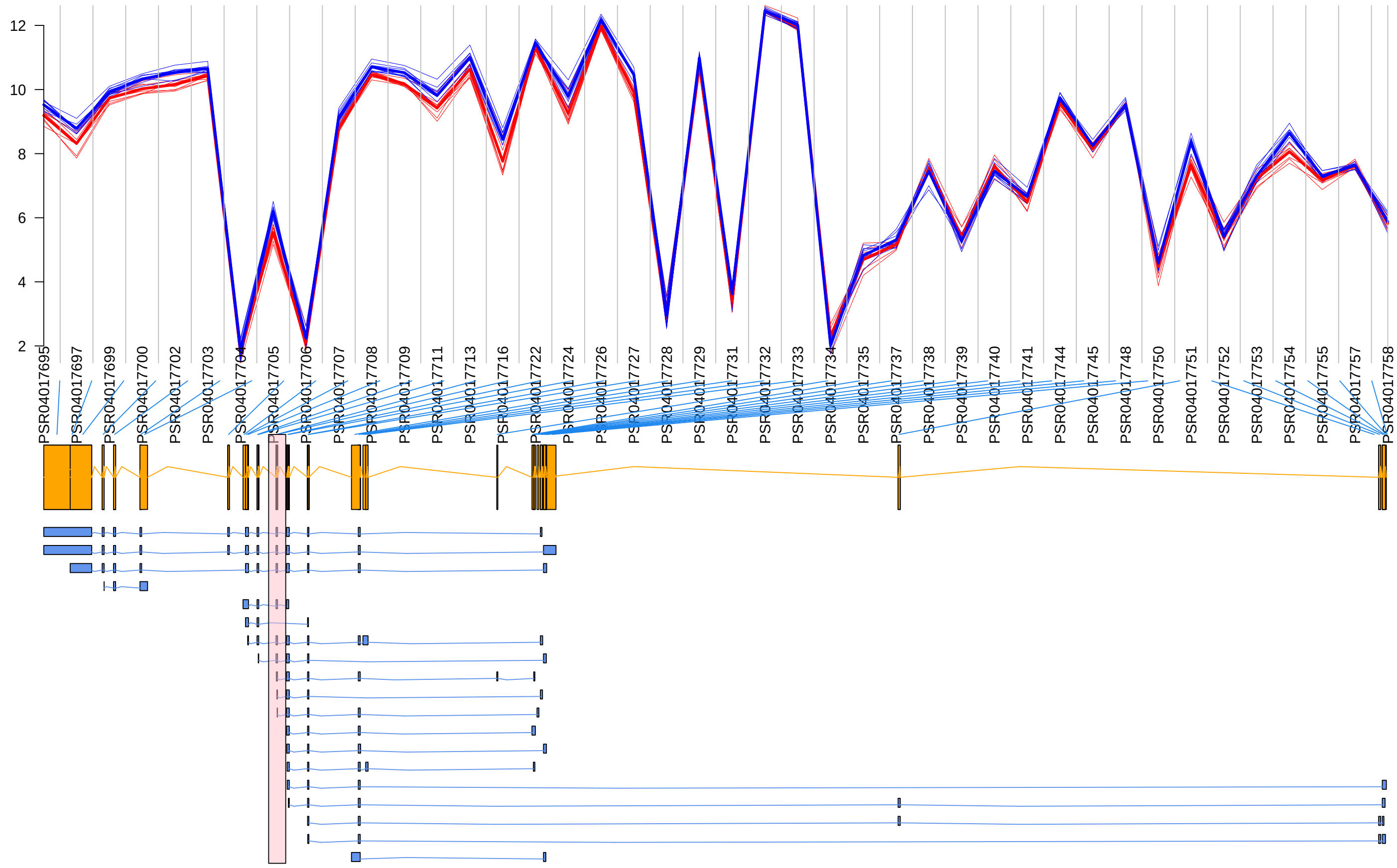

Figure S 10: The isoform composition of transcript cluster TC04001300 (G3BP2). Probe set PSR04017716 is highlighted as an alternative first event exon. The red lines represent the probe set expression levels of the SCR samples. The blue lines show the probe set expression levels for the siRNA samples.

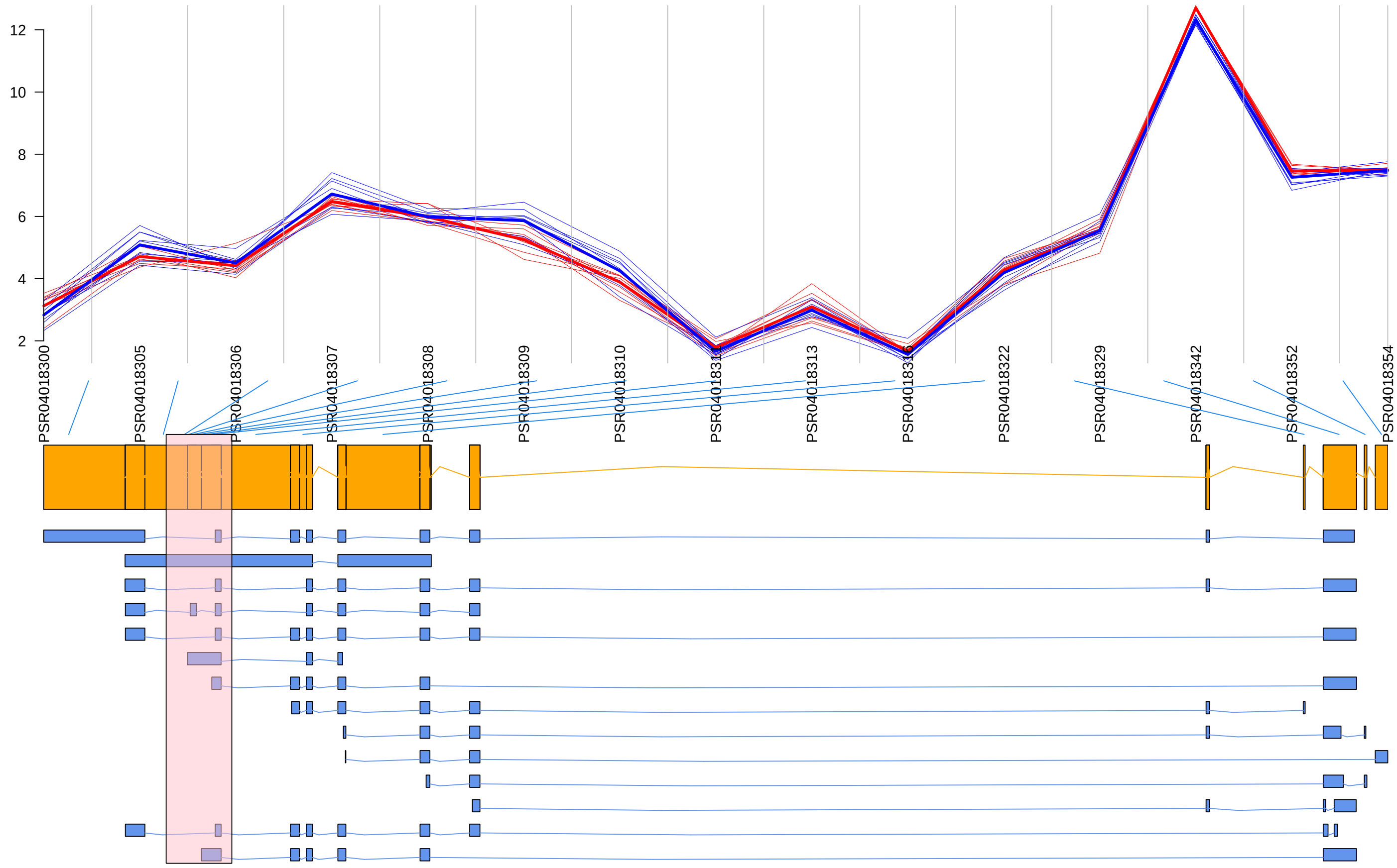

Figure S 11: The isoform composition of transcript cluster TC04001332 (HNRNPD). Probe set PSR04018315 is highlighted as an intron retention. The red lines represent the probe set expression levels of the SCR samples. The blue lines show the probe set expression levels for the siRNA samples.

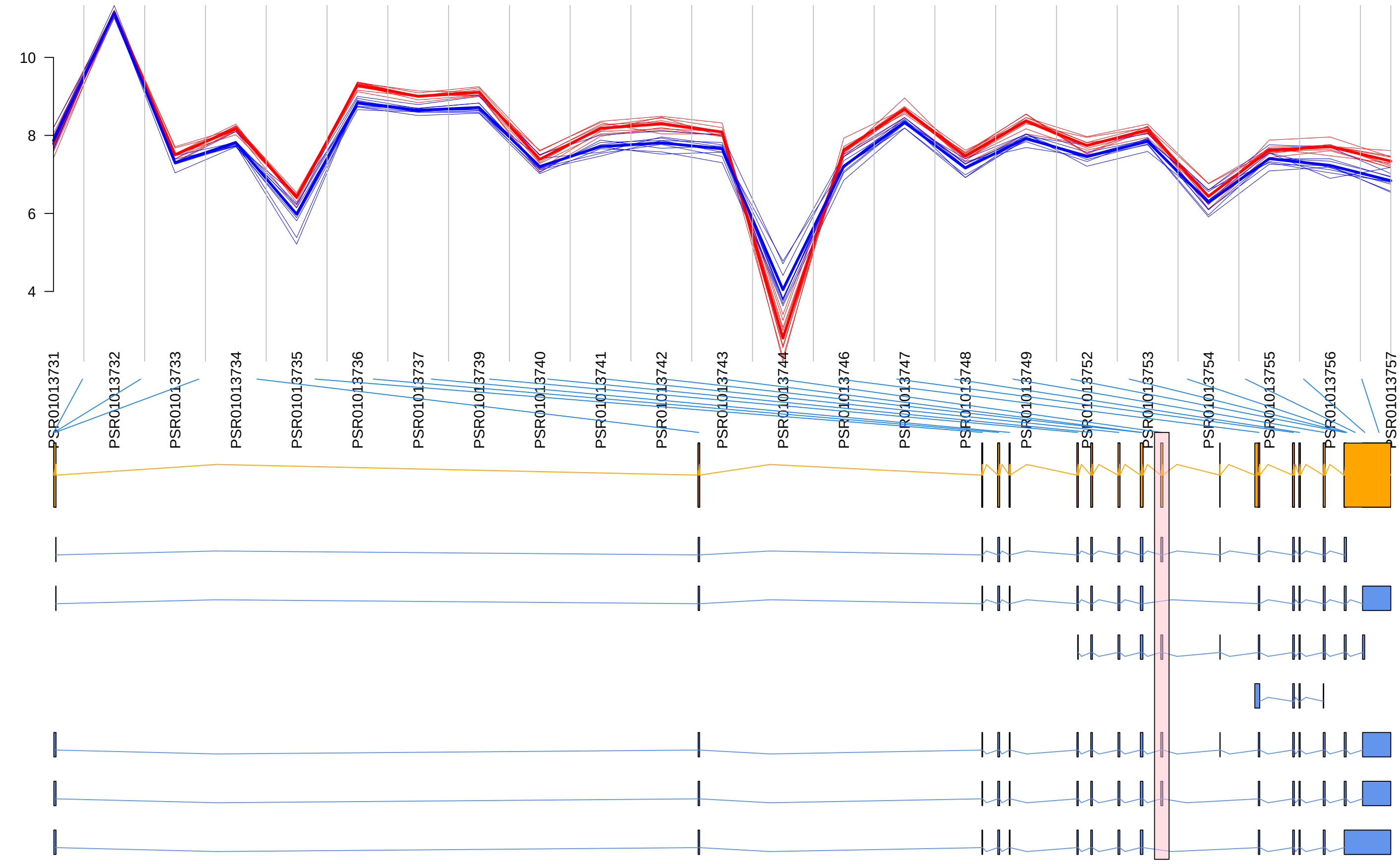

Figure S 12: The isoform composition of transcript cluster TC01000863 (FNBP1L). Probe set PSR01013744 is highlighted as a cassette exon. The red lines represent the probe set expression levels of the SCR samples. The blue lines show the probe set expression levels for the siRNA samples.

3 Results

3.1 Alternative Splicing with Supporting Junctions

Figure S13 illustrates the three exclusion junctions of probe set PSR010025633.

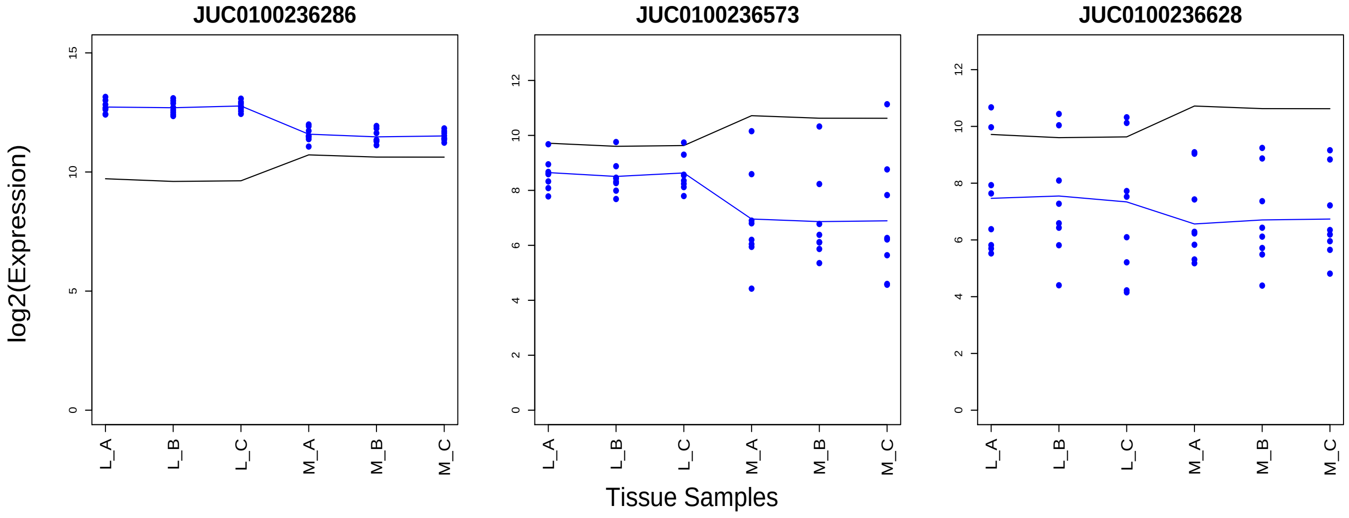

Figure S 13: The exclusion junctions annotated to PSR010025633 of transcript TC0102569 (CSDE1). The observed probe values of JUC0100236286, JUC0100236573 and JUC0100236628 relative the to the summarized gene level values of TC0102569. The black and blue lines indicate the mean profiles of the gene and exon level data respectively. The blue points show the probe level data.

Figure S14 illustrates the known transcript isoform composition of TC0102569. Probe set PSR010025633 is highlighted.

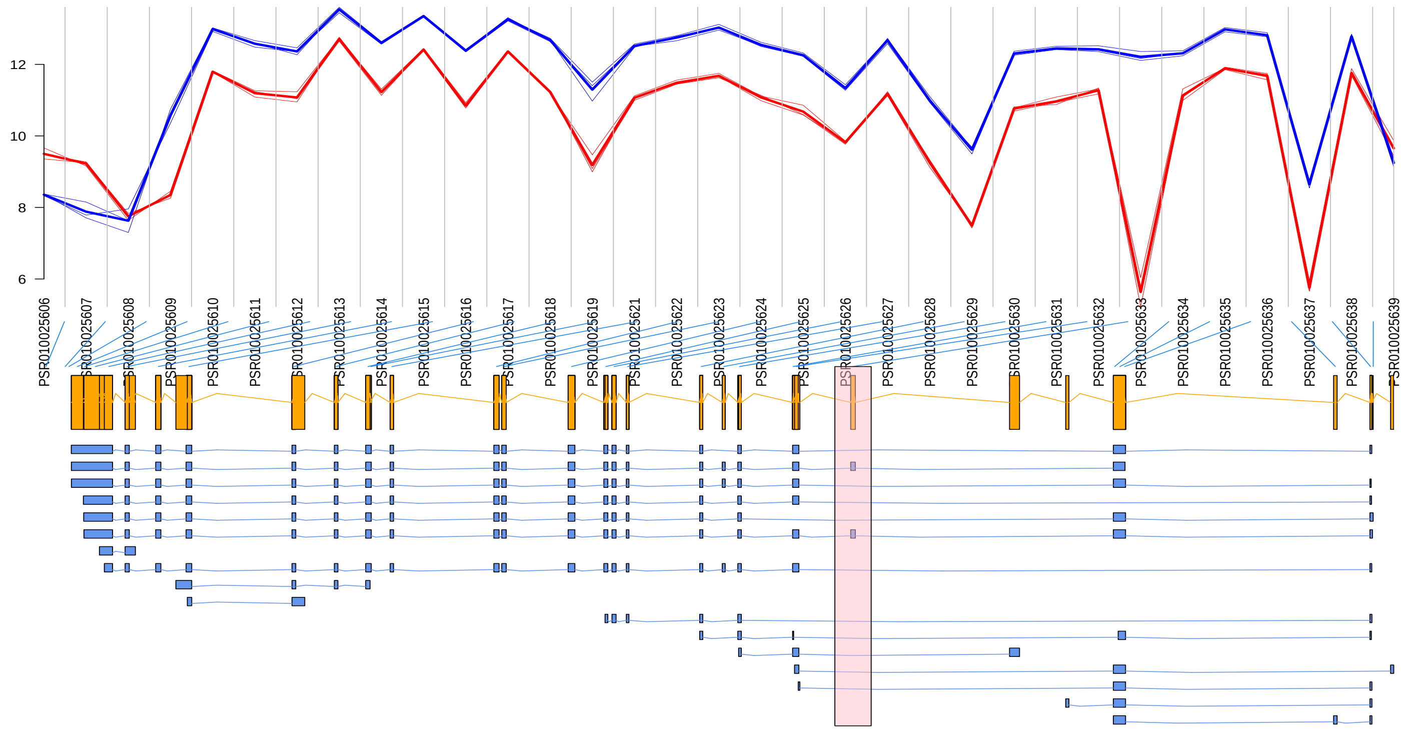

Figure S 14: The isoform composition of transcript cluster TC0102569 (CSDE1). Probe set PSR010025633 is highlighted as a cassette exon. The red lines represent the probe set expression levels of the liver samples. The blue lines show the probe set expression levels for the muscle samples.

3.2 Alternative Splicing with At Least One Supporting Junction

Figure S15 shows the junction design on the microarray for probe set PSR080007308 of transcript cluster TC0800969.

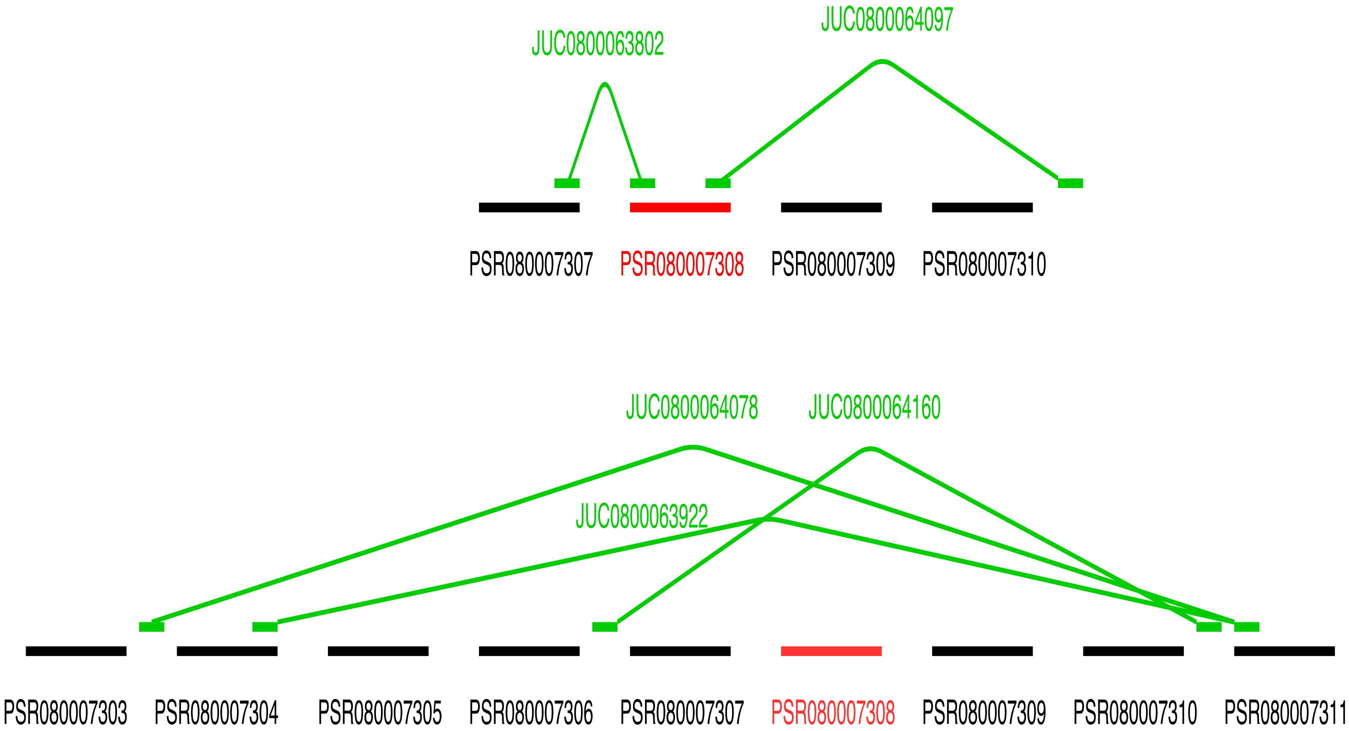

Figure S 15: The junction design of probe set PSR080007308 of transcript cluster TC0800969 (ASPH). Panel (a): The design of the 5′ and 3′ linking junctions of probe set PSR080007308. Panel (b): The design of the exclusion junctions of probe set PSR080007308.

Figure S16 shows the sequence of 5′ end junction JUC0800064097 and probe set PSR080007308.

```
T A C A A A A A A C A A T C A C C T G G G T C A C
  A C A A A A A A C A A T C A C C T G G G T C A C C
    C A A A A A A C A A T C A C C T G G G T C A C C C
      A A A A A A C A A T C A C C T G G G T C A C C C C
        A A A A A C A A T C A C C T G G G T C A C C C C A
          A A A A C A A T C A C C T G G G T C A C C C C A T
            A A A C A A T C A C C T G G G T C A C C C C A T C
              A A C A A T C A C C T G G G T C A C C C C A T C G
                                C T G G G T C A C C C C A T C G G T T C T C T T T
                                  T G G G T C A C C C C A T C G G T T C T C T T T T
                                      G G T C A C C C C A T C G G T T C T C T T T T T G
                                        G T C A C C C C A T C G G T T C T C T T T T T G A
                                          T C A C C C C A T C G G T T C T C T T T T T G A T
```

Figure S 16: The probe sequences of JUC0800064097 (blue) and PSR080007308 (green) of gene TC0800969 (ASPH) which are shown Figure S15. The overlap between the junction and the 5′ end of the probe set on the right is shown in yellow.

Figure S17 illustrates the known transcript isoform composition of TC0800969. Probe set PSR080007308 is highlighted.

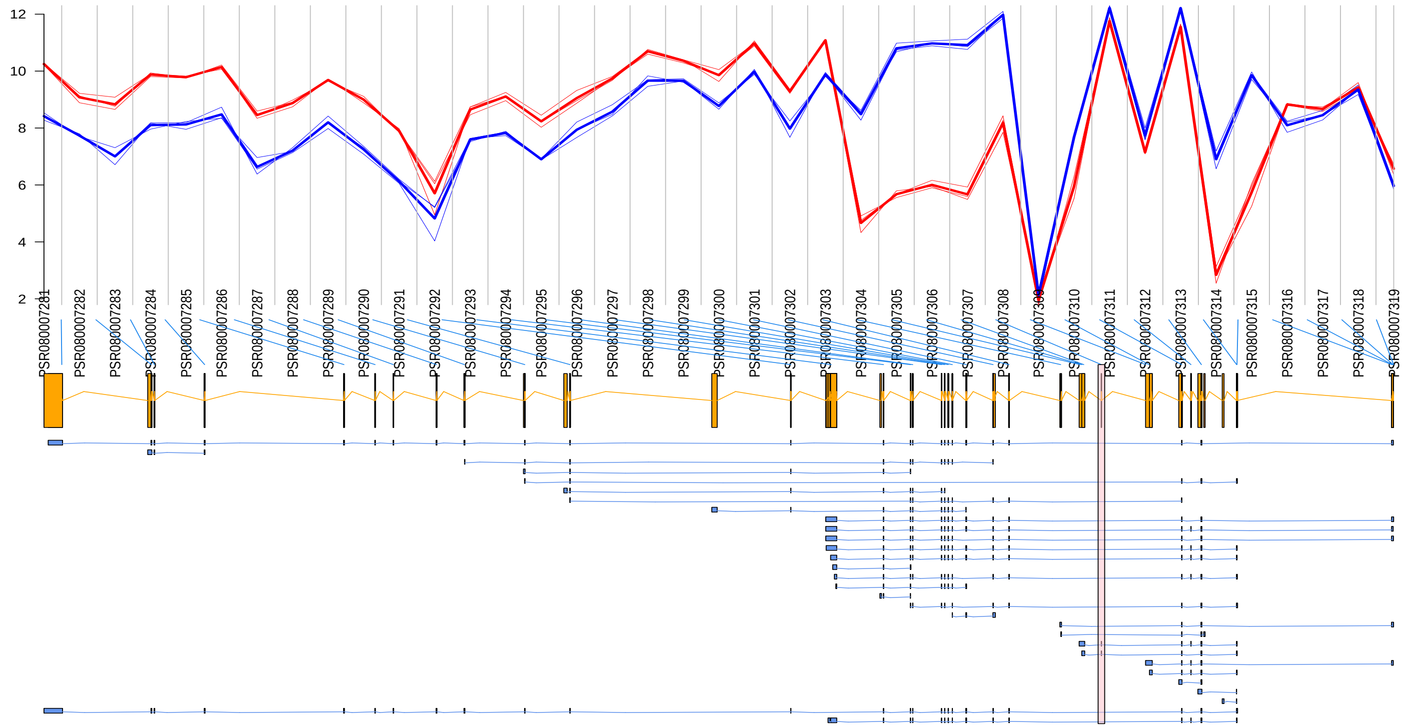

Figure S 17: The isoform composition of transcript cluster TC0800969 (ASPH). Probe set PSR080007308 is highlighted as a cassette exon. The red lines represent the probe set expression levels of the liver samples. The blue lines show the probe set expression levels for the muscle samples. The blue points show the probe level data.

Probe set PSR010004150 of transcript TC0100415 (MACF1 gene) is an example of a probe set for which not all junctions are supporting (category d in Figure 5 of the main manuscript). Figure S18 shows that the 3′ end junction JUC0100035871 does not reflect the pattern of their annotated probe sets. Junctions JUC0100031528, JUC0100032584 and JUC0100036148 are the end products of their annotated probe sets. The probe set is a reflection of the summarized gene expression, i. e. not alternatively spliced. However, note the alternative splicing of its neighbouring probe set PSR0100041549.

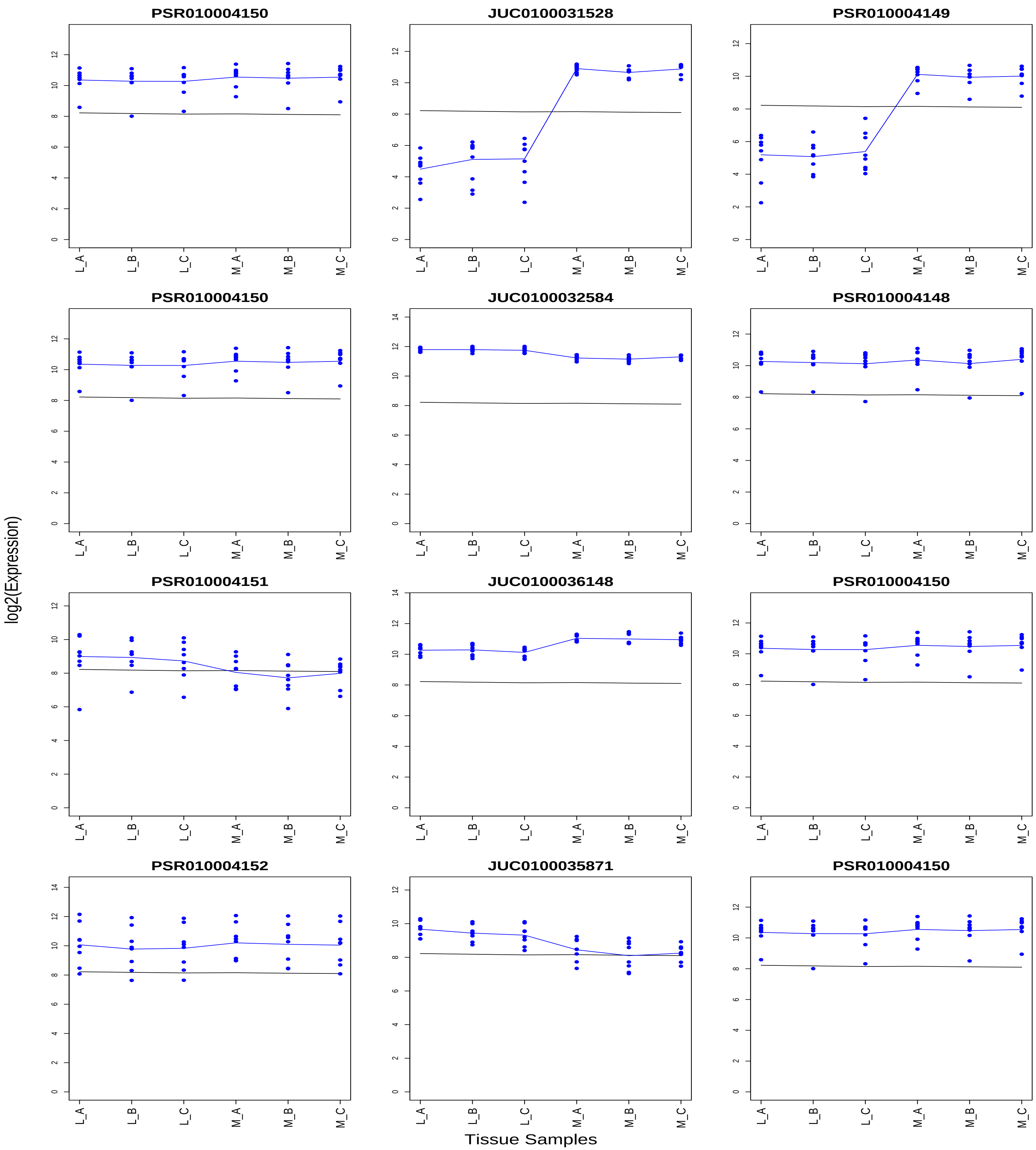

Figure S 18: An example of a probe set that was not supported by its annotated junctions to be alternatively spliced between the liver (L) and muscle samples (M): PSR010004150 of transcript TC0100415 (MACF1). The observed probe values of JUC0100035871, PSR010004150 and JUC0100031528 relative the to the summarized gene level values of TC0100415. The black and blue lines indicate the mean profiles of the gene and exon level data respectively. The blue points show the probe level data.

An additional example of a probe set with a single annotated junction is probe set PSR070008232 of transcript TC0700986 (ACTB gene) which only has a $3'$ end junction: JUC0700072879. The observed values of this probe set, the junction and the $5'$ end annotation of the junction, probe set PSR070008230, are shown in Figure S19. The junction supports probe set PSR070008232 in the alternative splicing identification and is therefore an example of category b of Figure 5 in the main manuscript.

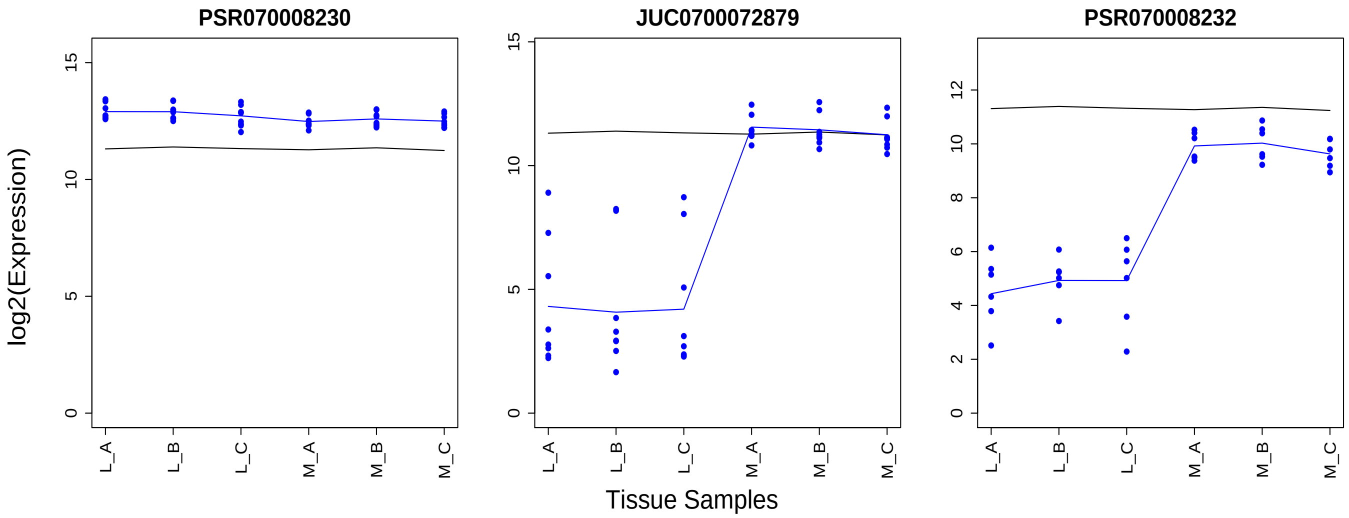

Figure S 19: An example of a probe set that was supported by at least one annotated junction to be alternatively spliced between the liver (L) and muscle samples (M): probe set PSR070008232 of transcript cluster TC0700986 (ACTB). The observed probe values of PSR070008230, JUC0700072879 and PSR070008232 relative the to the summarized gene level values of TC0700986. The black and blue lines indicate the mean profiles of the gene and exon level data respectively. The blue points show the probe level data.

3.3 Alternative Splicing Without Supporting Junctions

Figure S20 shows the known transcript isoform composition of TC0101665.

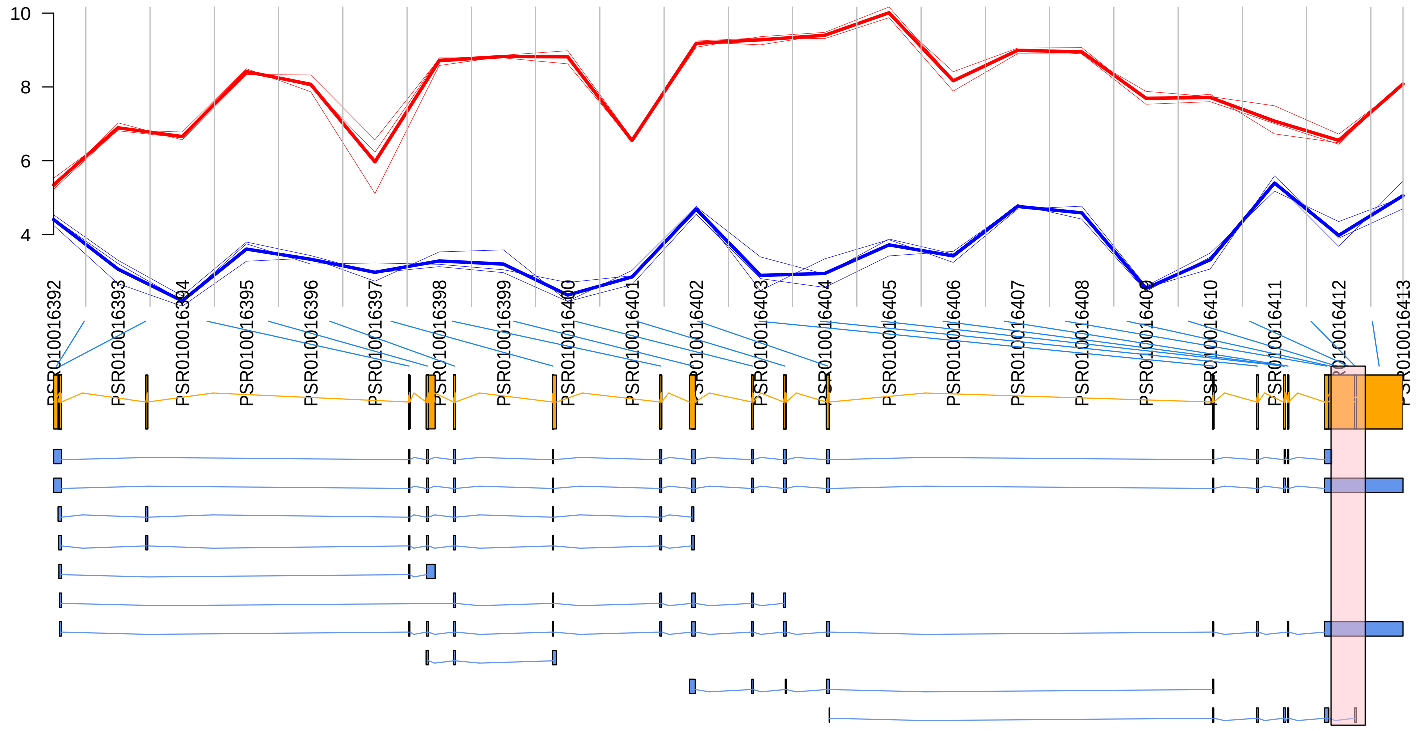

Figure S 20: The isoform composition of transcript cluster TC0101665 (KMO). Probe set PSR010016411 is highlighted. The red lines represent the probe set expression levels of the liver samples. The blue lines show the probe set expression levels for the muscle samples.

3.4 Junction Design

3.4.1 PSR190012156

In this section we present an example of exon probe set PSR190012156 of transcript cluster transcript TC1901243 (DNAJB1 gene) for which the junctions on the 5′ end do not match the pattern of the probe set.

Figure S21 presents the probe set, its two 5′ end junctions and probe set PSR190012157. We notice that the junctions do not confirm the observed alternative splicing of the probe set.

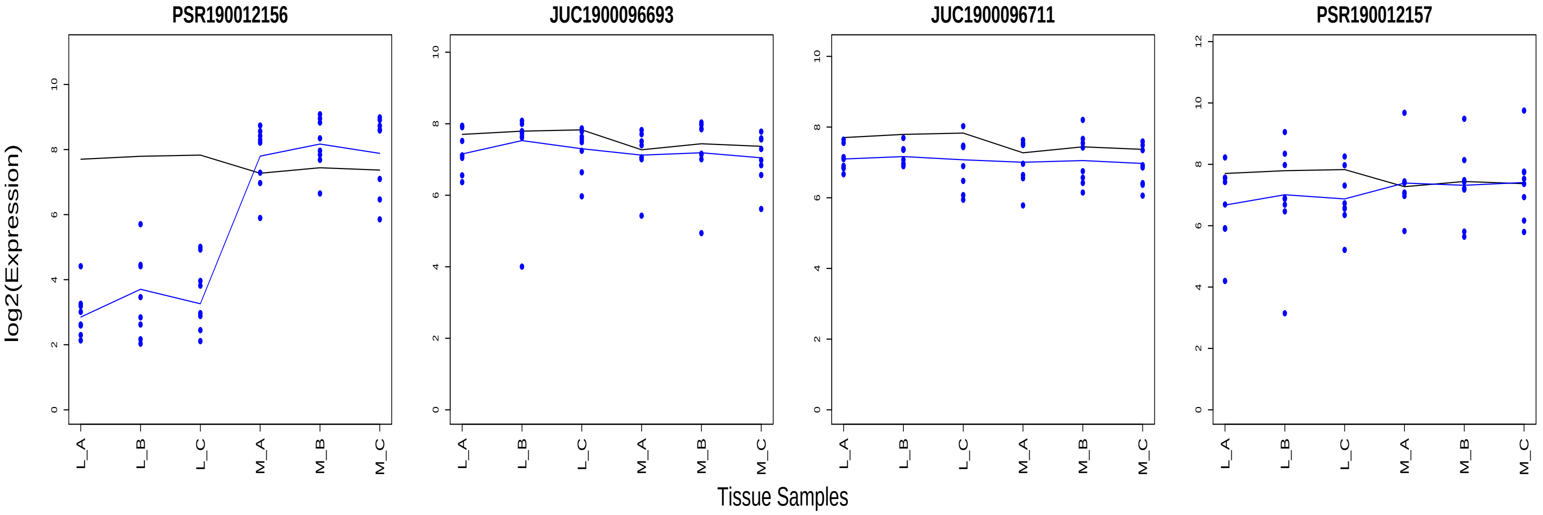

Figure S 21: An example of a probe set that was not supported by its annotated junctions to be alternatively spliced between the liver (L) and muscle samples (M): PSR190012156 of transcript cluster TC1901243 (DNAJB1). The observed probe values of PSR190012156, JUC1900096693, JUC1900096711 and PSR190012157 of transcript cluster of TC1901243 (DNAJB1) relative to the summarized gene level values. The black and blue lines indicate the mean profiles of the gene and exon level data respectively. The blue points show the probe level data.

Junction JUC1900096693 is a 5′ end junction of the probe set and has no other recorded annotation. However, its sequence does matches the sequence of probe set PSR190012157 which was found using the Ensembl genome browser. Junction JUC1900096711 is annotated to probe set PSR190012157 as well and this was recorded by the provided annotation file. Figure S22 shows that the start of the sequences of the junctions JUC1900096711 and JUC1900096693 on the 3′ end (which should resemble probe set PSR190012157) differ.

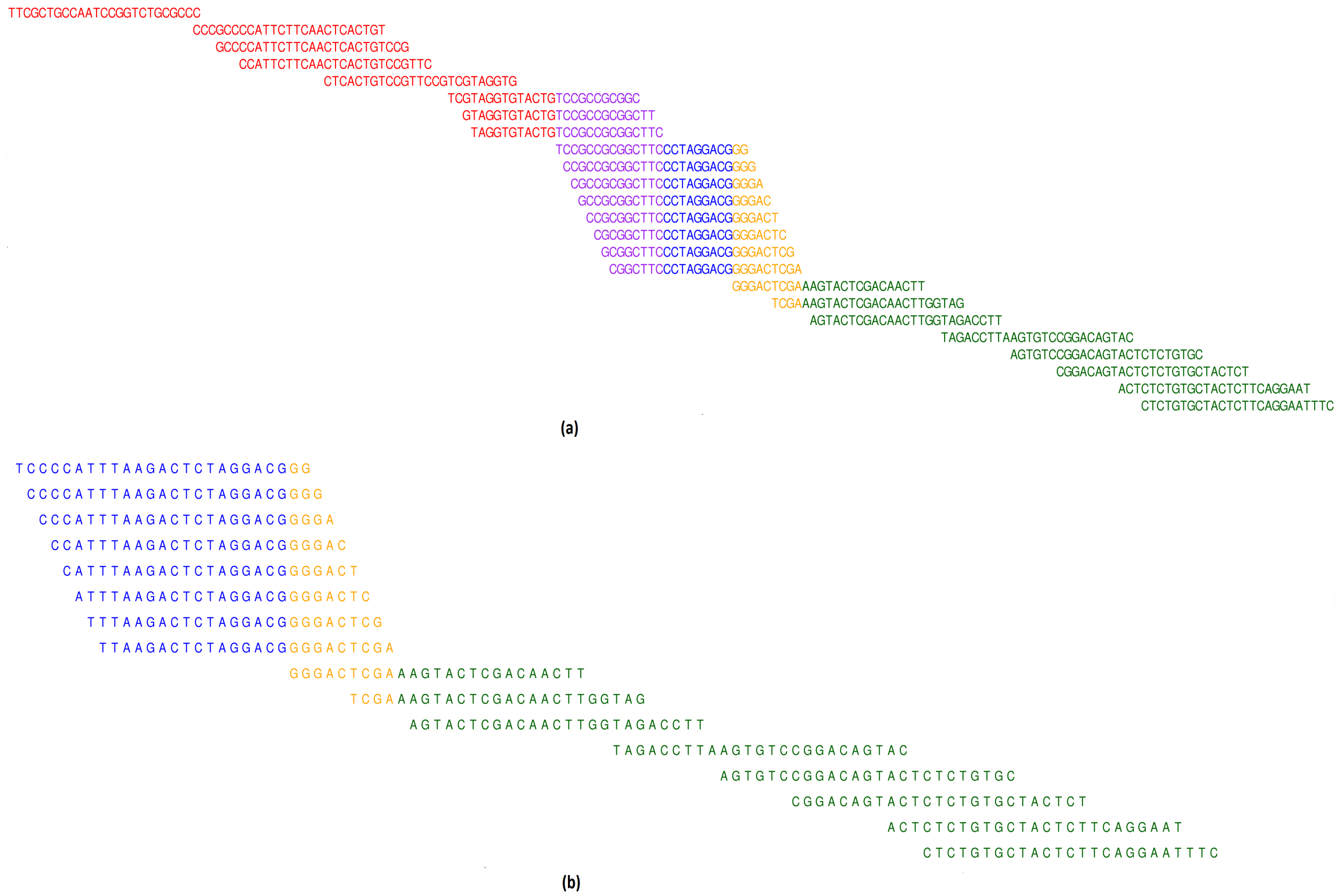

Figure S 22: Panel (a): The probe sequences of PSR190012157 (red), JUC1900096711 (blue) and PSR190012156 (green) of TC1901243 (DNAJB1). The overlap between the junction and the 3′ end of the probe set on the left is shown in purple and yellow reflects the overlap between the junction the 5′ end of the probe set on the right. Panel (b): The probe sequences of JUC1900096693 (blue) and PSR190012156 (green) of TC1901243 (DNAJB1) The overlap between the junction and the 5′ end of the probe set on the left is shown in yellow.

The whole sequence of probe set PSR190012157 is:

TTCGCTGCCAATCCGGTCTGCGCCCCGCCCCATTCTTCAACTCACTGT
CCGTTCCGTCGTAGGTGTACTGTCCGCCGCGGCTTCCCCATTTAAGACTC

We observe that the junctions refer to different parts of probe set PSR190012157. Both junctions indicate the presence of PSR190012156 and PSR190012157 in all samples. This could imply that in the transcript isoform collections two transcriptions of probe set PSR190012157 are present, i.e. with and without the last base pairs. A possible explanation for the presence of the junction but not of the probe set PSR190012156 could be just a partial transcription of the probe set e.g. if the transscription ends prematurely. Consequently, the first few base pairs of PSR190012156 are transcribed, and thus the junction as well, yet not the whole probe set can be found in the cDNA and therefore cannot be measured. The 3′ end junction JUC1900096716 and its annotations are shown in Figure S23.

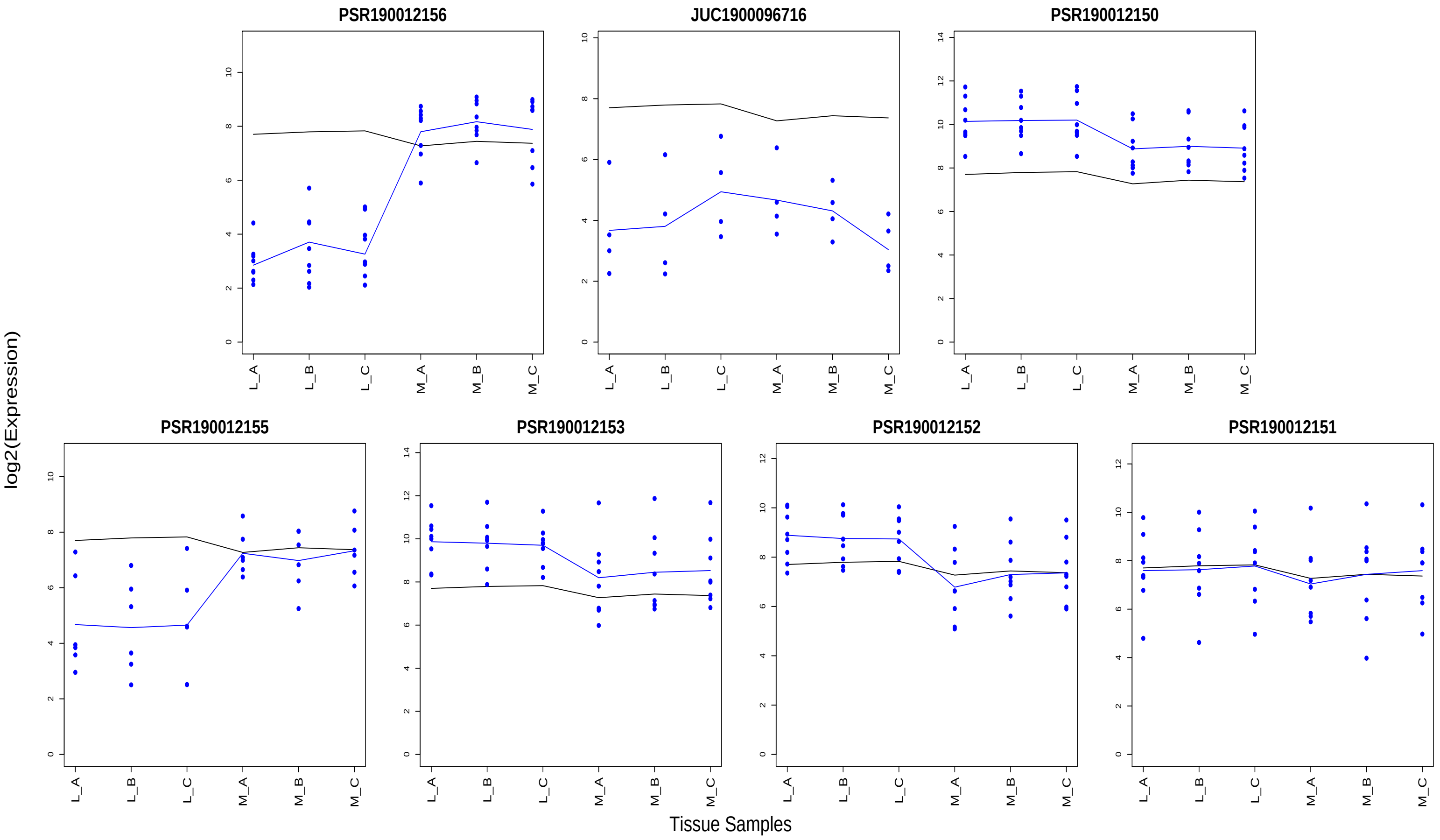

Figure S 23: The linking junctions of probe set PSR190012156 of transcript cluster TC1901243 (DNAJB1). The observed probe values of respectively PSR190012156, JUC1900096716,PSR190012150, PSR190012155, PSR190012153, PSR190012152 and PSR190012151 relative the to the summarized gene level values of TC1901243 (DNAJB1). The black and blue lines indicate the mean profiles of the gene and exon level data respectively. The blue points show the probe level data.

The probe set also has an annotation to a 3′ end junction JUC1900096716. The depletion of junction JUC1900096716 in the liver samples can be caused by the depletion of its anchor point PSR190012156 or by the inclusion of some of the probe sets for which junction JUC1900096716 is an exclusion junction. The latter is the cause for the lower values of the junction in the muscle samples for which both anchor points are present. Based on visual evidence, these junctions should be treated as unreliable. However, even unexpected behaviour of the junctions might help us to disentangle the presence of several transcript isoform in a cell. Since we assessed that both the 3′ end junctions JUC1900096711 and JUC1900096693 are present, a link between PSR190012156 and PSR190012157 exists for both versions of PSR190012157. This implies that a transcript isoforms can either contain probe set PSR190012157 as is as well as a prematurely ended (exon splitted) version of the exon.

3.4.2 PSR120012240

The linking and exclusion junctions of probe set PSR120012240 (shown in Figure S24) of TC1201288 is annotated to the SMARCC2 gene. This probe set has three junctions: a single 5′ end junction and two 3′ end junctions. The 5′ end junction JUC1200112452 is annotated to the adjacent probe set PSR120012241. The presence of both probe sets is confirmed by the junction.

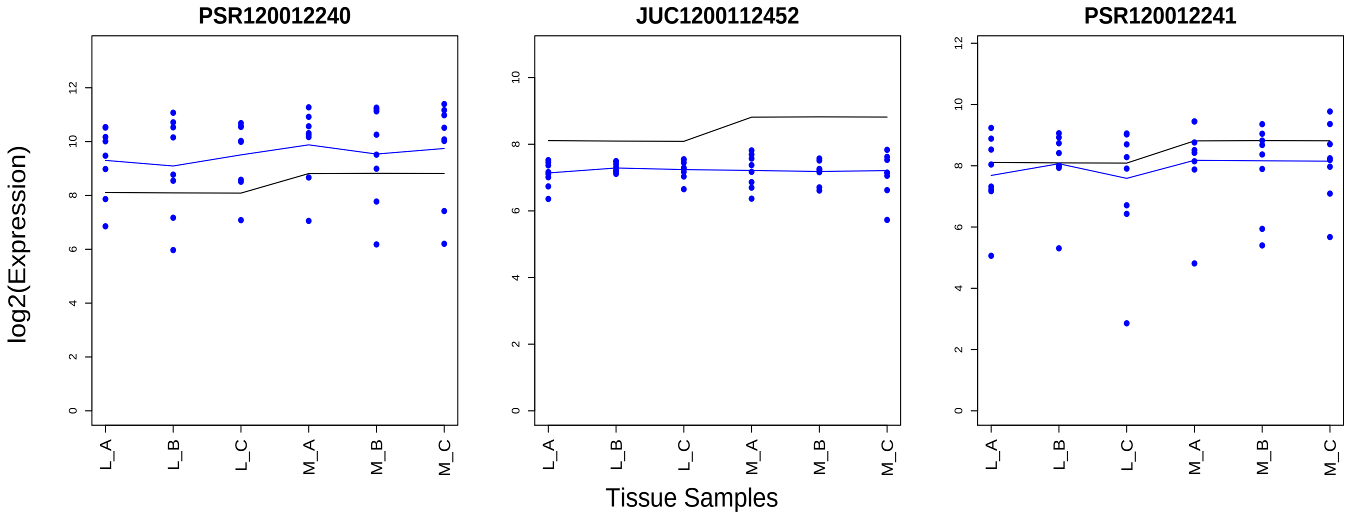

Figure S 24: The linking junctions of probe set PSR120012240 of transcript cluster TC1201288 (SMARCC2). The observed probe values of PSR120012240, JUC1200112452 and PSR120012241 relative the to the summarized gene level values of TC1201288. The black and blue lines indicate the mean profiles of the gene and exon level data respectively. The blue points show the probe level data.

Junction JUC1200112431 is a 3′ end junction of probe set PSR120012240 and a 5′ end junction of PSR120012237. It skips over probe sets PSR120012238 and PSR120012239. The values of these four probe sets and the junction are shown in Figure S25.

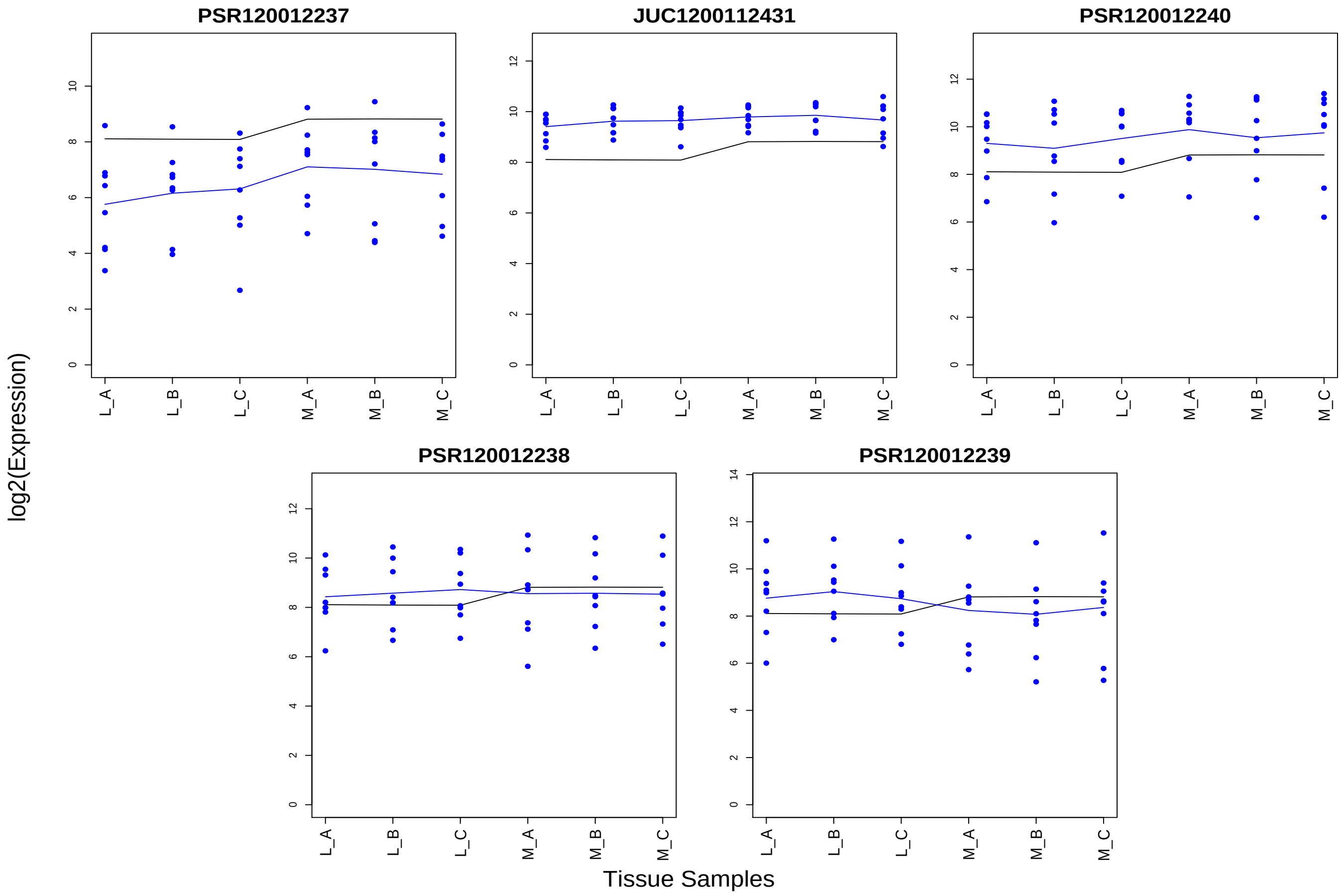

Figure S 25: The linking junctions of probe set PSR120012237 of transcript cluster TC1201288 (SMARCC2). The observed probe values of respectively PSR120012237, JUC1200112431, PSR120012240, PSR120012238 and PSR120012239 relative to the summarized gene level values of TC1201288. The black and blue lines indicate the mean profiles of the gene and exon level data respectively. The blue points show the probe level data.

Note that although the expression values of the junction JUC1200112431 are of the same magnitude and show a similar pattern as PSR120012240, it shows an opposite pattern compared to the expression pattern of PSR120012237. The presence of the probe sets PSR120012238 and PSR120012239 which should interrupt the sequence of the junction do not seem to influence the junction values. Since the junction is present, there must be a transcript that contains both PSR120012237 and PSR120012240 but not probe sets PSR120012238 and PSR120012239. Further, a transcript with PSR120012238 and PSR120012239 should be observed as well. The sequence of PSR120012240, JUC1200112431 and PSR120012237 is shown in Figure S26.

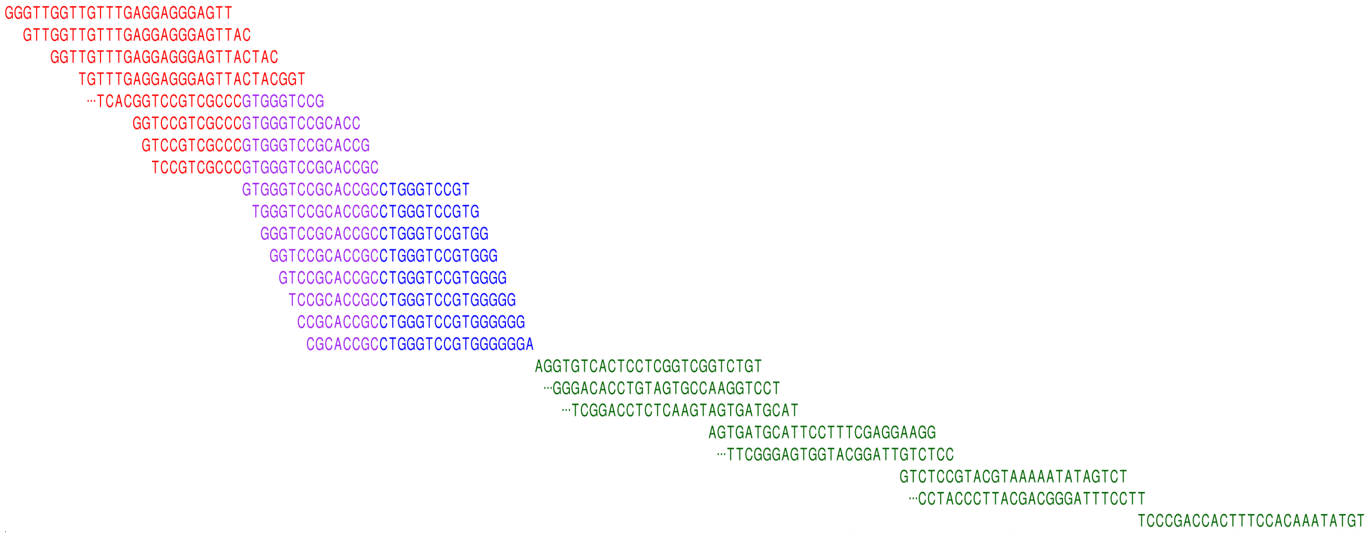

Figure S 26: The probe sequences of PSR120012240 (red), JUC1200112431 (blue) and PSR120012237 (green) of gene TC1201288 (SMARCC2). The overlap between the junction and the 3′ end of the probe set on the left is shown in purple and yellow reflects the overlap between the junction the 5′ end of the probe set on the right.

The junction shows no match with the designed probes for probe set PSR120012237. The junction does matches with the first base pairs of probe set PSR120012237 which whole sequence (found by the Ensemble browser) is:

TGGGTCCGTGGGGGGACGGAGGTCTGGGGTGTCGGGGCTCGGGTCCGTGCCAGTGGGGAC
ACGGTGGAGGTGTCACTCCTCGGTCGGTCTGTAGAGAGGGGGAGTGGGGGACACCTGTAG

It seems that the first base pairs of PSR120012237 are transcribed in all samples but the latter are not. This implies that the junction is present even when the probe set is not. This could explain why the expression values of the junction are higher than those of PSR120012237. Possible reasons are a premature ending of the transcription or different 3′ UTR regions between the tissues. Figure S27 shows the second 3′ end junction JUC1200112702 which has a 5′ end annotation to PSR120012238 and is an exclusion junction for probe set PSR120012239. Junction JUC1200112702 shows the presence of PSR120012238 adjacent to probe set PSR120012240. However, probe set PSR120012239 is present as well which disrupts the sequence of the junction. This again informs us about the composition of the transcript isoforms.

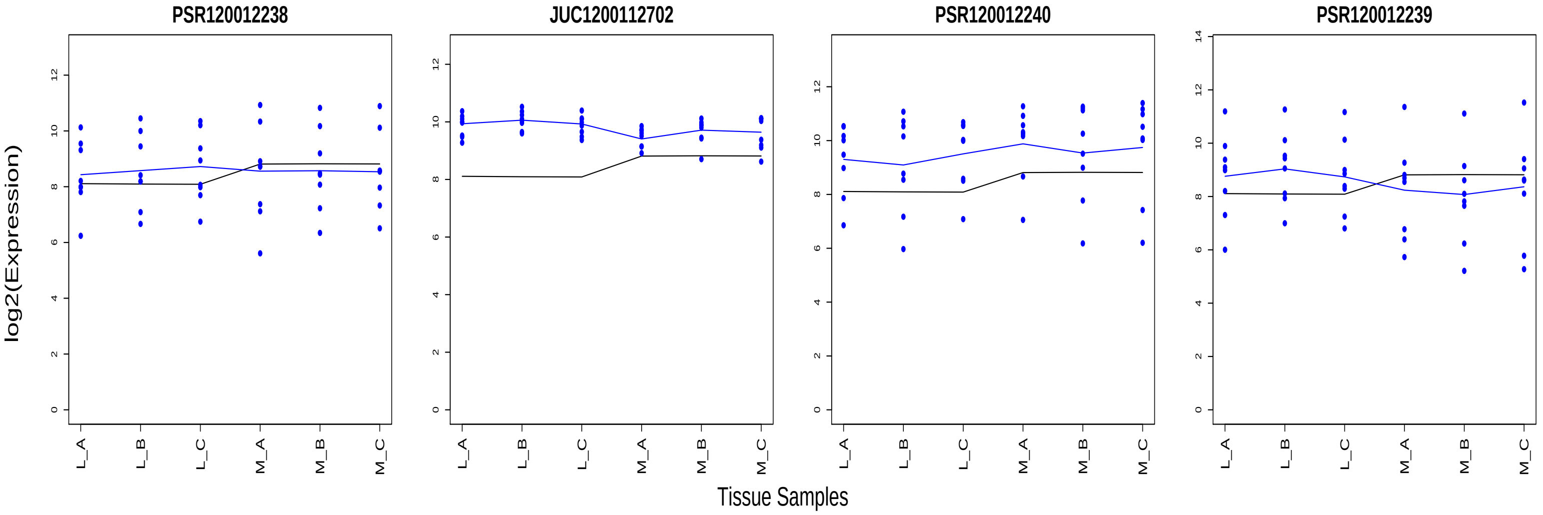

Figure S 27: The linking junctions of probe set PSR120012238 of transcript cluster TC1201288 (SMARCC2). The observed probe values of PSR120012238, JUC1200112702, PSR120012240 and PSR120012239 relative the to the summarized gene level values of TC1201288. The black and blue lines indicate the mean profiles of the gene and exon level data respectively. The blue points show the probe level data.

3.5 Examples of Probe Sets Supported by all Linking Junctions

3.5.1 Probe set PSR030011882

Figure S28 shows the observed values and array scores probe set PSR030011882 of transcript cluster TC0301262 (ITIH4 gene) and its annotated junctions JUC0300113792, JUC03001141211, JUC0300113838 and JUC0300113913.

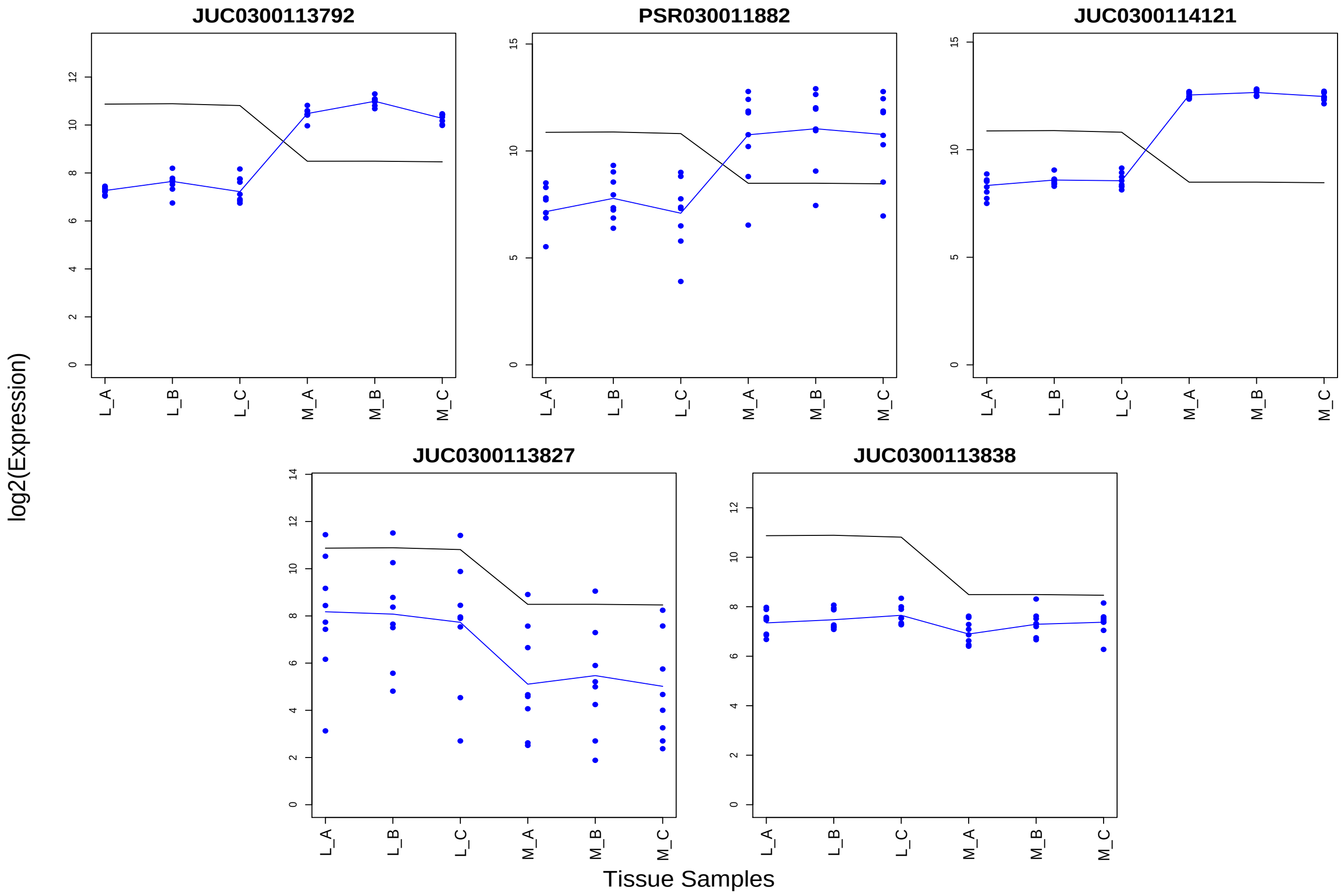

Figure S 28: The linking and exclusion junctions of probe set PSR030011882 of transcript cluster TC0301262 (ITIH4). The observed probe values of JUC0300113792, PSR030011882 JUC03001141211, JUC0300113838 and JUC0300113913. The black and blue lines indicate the mean profiles of the gene and exon level data respectively. The blue points show the probe level data.

3.6 Study of the Transcript Cluster TC1601187

The transcript cluster TC1601187 is associated with the genes LCAT and SLC12A4. Table S2 is a summary of the results of the REIDS model for the probe set regions and junctions annotated to this transcript that have been identified by RASA and verified by RNA sequencing.

Table S 2: Probe sets PSR160012165, PSR160012168, PSR160012166, PSR160012163, PSR160012170 and their adjacent junctions of transcript cluster TC1601187.

TC ID	PSR ID	ExonScore	t.stat	p-val	Type	Rank	JUC ID	Type	As Type
TC1601187	PSR160012165	0.93	38.10	< 0.01	alt	3333	JUC1600099622	alt	3
							JUC1600099807	alt	5
	PSR160012168	0.93	39.70	< 0.01	alt	4048	JUC1600099690	alt	3
							JUC1600100115	alt	5
	PSR160012166	0.91	47.11	< 0.01	alt	2967	JUC1600099807	alt	3
							JUC1600100115	alt	5

The selected probe sets are also identified by the REIDS model with supporting junctions. The plots in Figure S29 show that the transcript is not differentially expressed between the tissues and that these probe sets have observations indicating alternatively splicing.

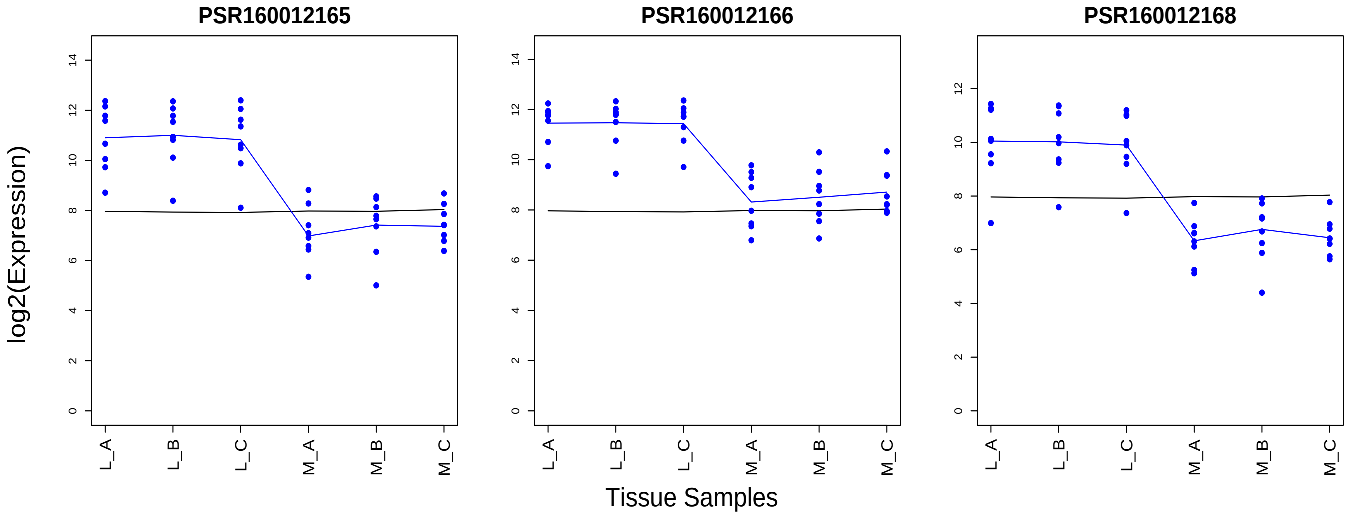

Figure S 29: Illustration of probe sets PSR160012165, PSR160012166 and PSR160012168 of transcript cluster TC1601187. The black and blue lines indicate the mean profiles of the gene and exon level data respectively. The blue points show the probe level data.

The REIDS model however identifies more probe sets of this transcript as alternatively spliced than have been verified. Some of these probe sets could be selected for further research to detect whether these are truly a AS event. The REIDS model however identifies more probe sets of this transcript as alternatively spliced than have been verified. Some of these probe sets could be selected for further research to detect whether these are truly a AS event. This implies that these probe sets have passed the exon score threshold and the array score of the sample groups differ significantly. The difference in array scores can be visualized with density plots as shown in Figure S29. A bimodal distribution shows the separation of the tissues for which the values of the array scores are indicated in red (liver) and blue (muscle).

An example is PSR160012167 illustrated in Figure S30. Others, however, do not seem to have observations that are an indication of alternative splicing although the REIDS model identifies them as such. With only three replicates for each tissue and little variance between these respective replicates, even a small difference is seen as significant while these are likely to be artefacts of the measuring. In Figure S31 PSR160012168 is presented which is likely not alternatively spliced.

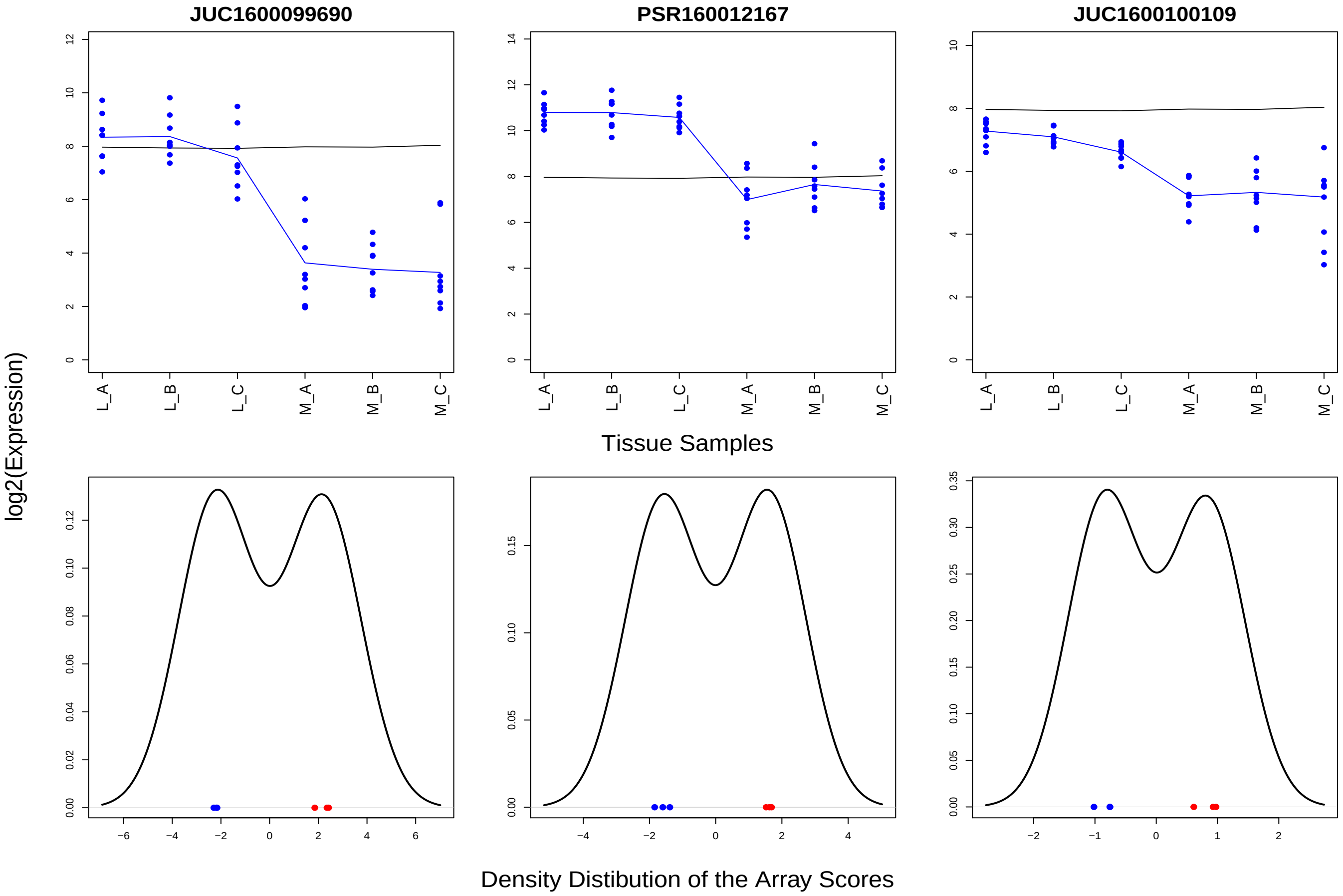

Figure S 30: The linking junctions of probe set PSR160012167 of transcript cluster TC1601187. Upper panel: the observed probe values of JUC1600099690, PSR160012167 and JUC1600100109. The black and blue lines indicate the mean profiles of the gene and exon level data respectively. The blue points show the probe level data. Lower Panel: density plots of the array scores of JUC1600099690, PSR160012167 and JUC1600100109 and values of the array scores are indicated in red (liver) and blue (muscle).

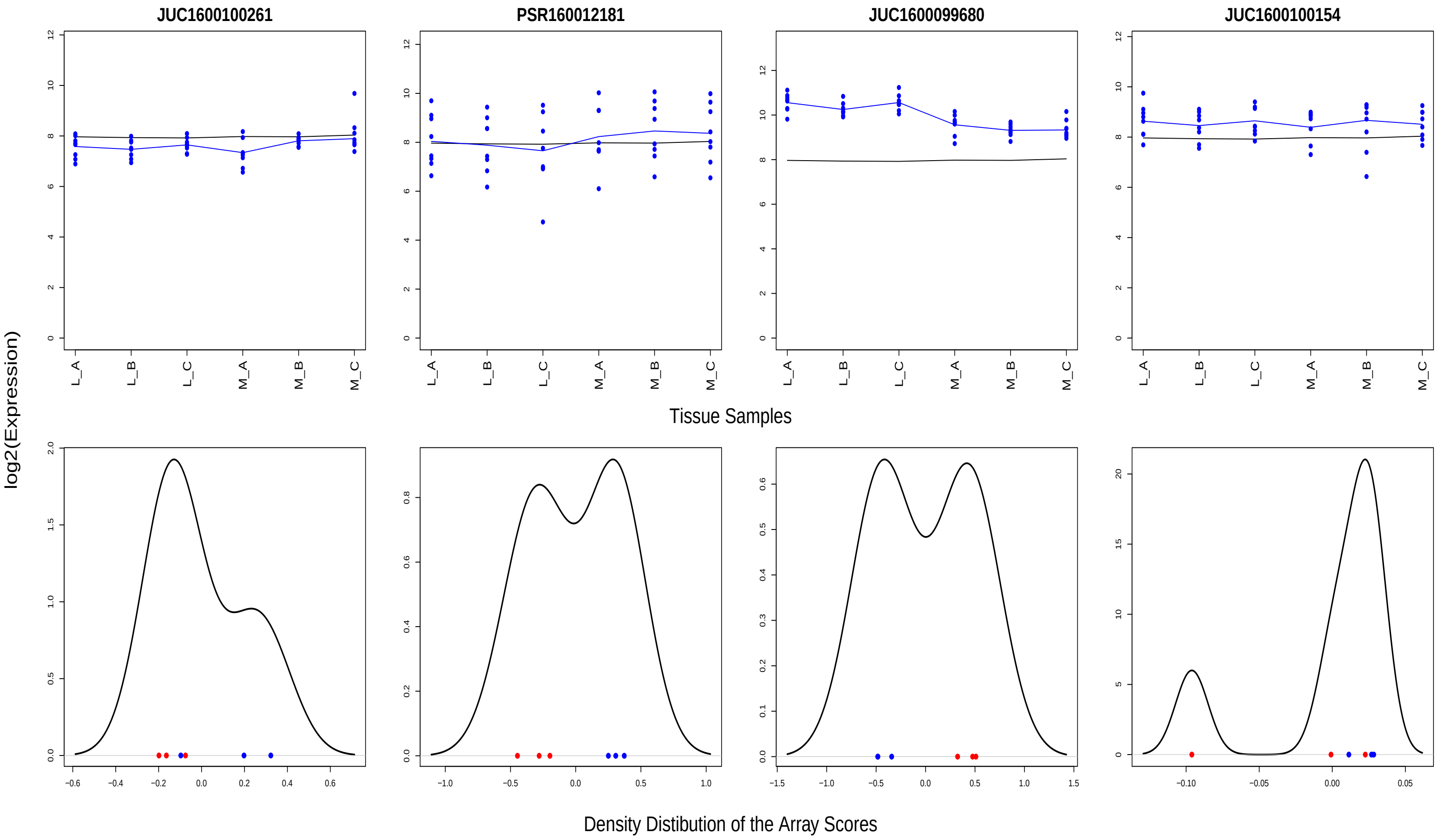

Figure S 31: The linking junctions of probe set PSR160012181 of transcript cluster TC1601187. Upper panel: the observed probe values of JUC1600100261, PSR160012181 JUC1600099680 and JUC1600100154. The black and blue lines indicate the mean profiles of the gene and exon level data respectively. The blue points show the probe level data. Lower Panel: density plots of the array scores of JUC1600100261, PSR160012181, JUC1600099680 and JUC1600100154 values of the array scores are indicated in red (liver) and blue (muscle).

3.7 Validation of the REIDS Model

Figure S32 shows an illustrative example of one exon, PSR120013728 of transcript cluster TC1201436 (POC1B), and its 5′end JUC0300131720 and 3′end JUC0300131708 junction. The probe set is identified by RASA as AS but not by REIDS. For this exon, with an exon score equal to 0.42, the REIDS model does not indicate an alternative splicing event. Note that, with exon score lower than 0.5, the PSR120013728 probe set was not selected as an AS candidate among the 17,700 exons selected for the analysis in stage two.

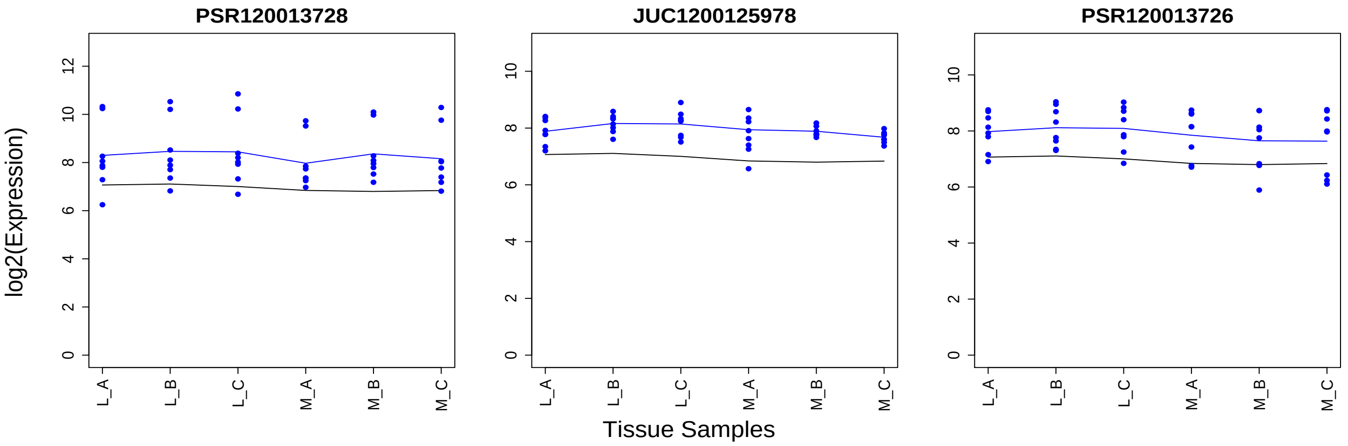

Figure S 32: An example of a probe set identified by RASA but not by REIDS in the HJAY data set: PSR120013728 of transcript cluster TC1201436 (POC1B). The observed probe intensities of PSR120013728, JUC1200125978 and PSR120013726 relative to the summarized gene level values of TC1201436. The black and blue lines indicate the mean profiles of the gene and exon level data respectively. The blue points show the probe level data.

PSR120013728 is classified as a cassette exon as shown in Figure S33.

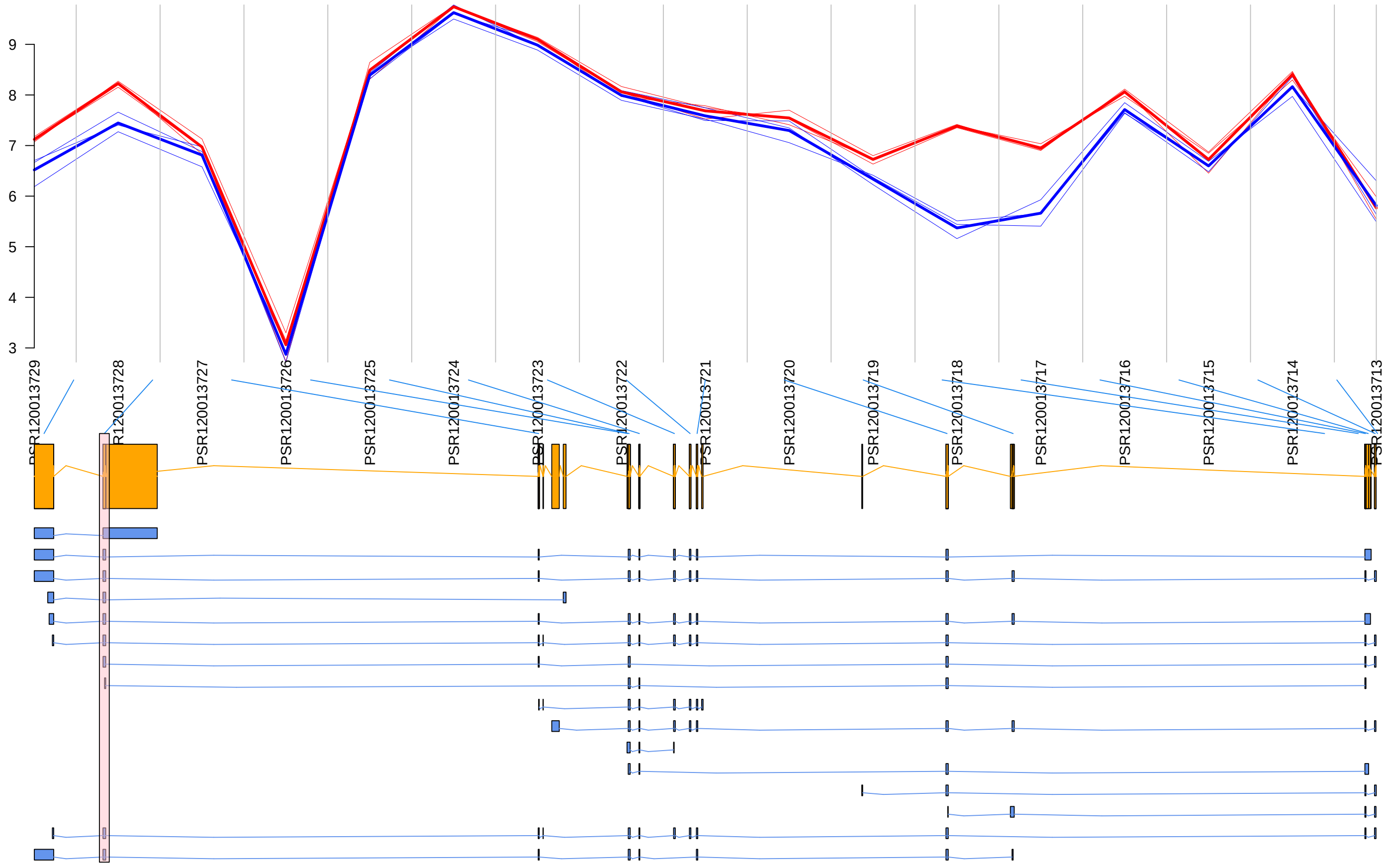

Figure S 33: The isoform composition of transcript cluster TC1201436 (POC1B). Probe set PSR120013728 is highlighted as a alternative first exon. The red lines represent the probe set expression levels of the liver samples. The blue lines show the probe set expression levels for the muscle samples.

Figure S34 illustrates the possible isoform transcriptions of TC1201436 as identified with the microarray. Black probe sets are identified as constituitive while coloured probe sets were identified as AS. A green colour indicates a enrichment of the probe sets and a red colour a depletion. Junction which are DABG are shown in blue while depleted junctions are again shown in red. Grey linkages are illustrative and have no meaning matching the measured data. The upper panel shows the probe sets for the liver samples and the lower panel for the muscle samples. PSR120013728 is not identified by the REIDS model.

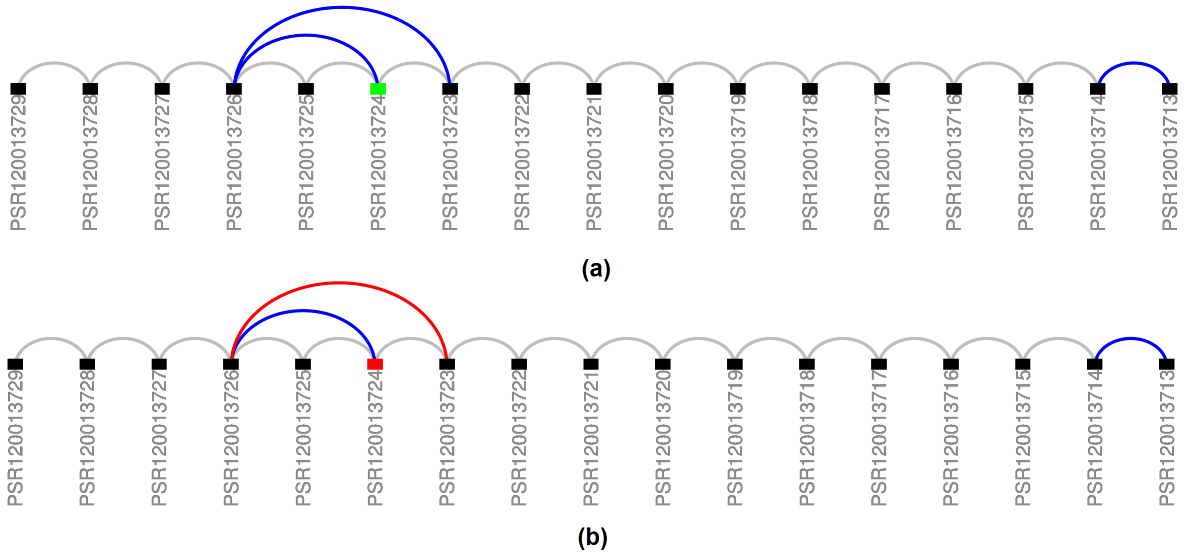

Figure S 34: The gene design for transcript cluster TC1201436 (POC1B). Black probe sets are identified as constituitive while coloured probe sets were identified as AS. A green colour indicates a enrichment of the probe sets and a red colour a depletion. Junction which are DABG are shown in blue while depleted junctions are again shown in red. Grey linkages are illustrative and have no meaning matching the measured data. Panel (a) shows the probe sets for the liver samples and panel (b) for the muscle samples.

Exon probe set PSR050011925 of transcript cluster TC0501406 (CAMK2A) is identified by REIDS (exon score=0.90 and p-value<0.01) as AS but not by RASA and is presented in Figure S35. We notice that the $5'$end junction (JUC0500105725) and the $3'$end junction (JUC0500105779) follow the splicing pattern of the exon.

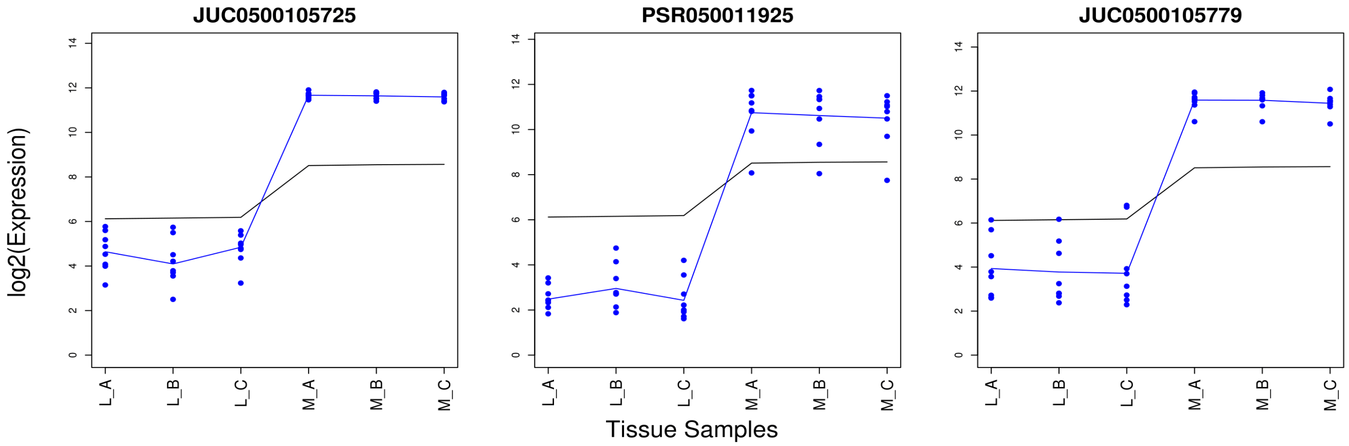

Figure S 35: An example of a probe set identified by REIDS but not by RASA in the HJAY data set: PSR050011925 of transcript cluster TC0501406 (CAMK2A). The observed probe intensities of JUC0500105725, PSR050011925, JUC0500105779 relative to the summarized gene level values of TC0501406. The black and blue lines indicate the mean profiles of the gene and exon level data respectively. The blue points show the probe level data.

PSR050011925 is classified as an alternative last even as shown in Figure S36.

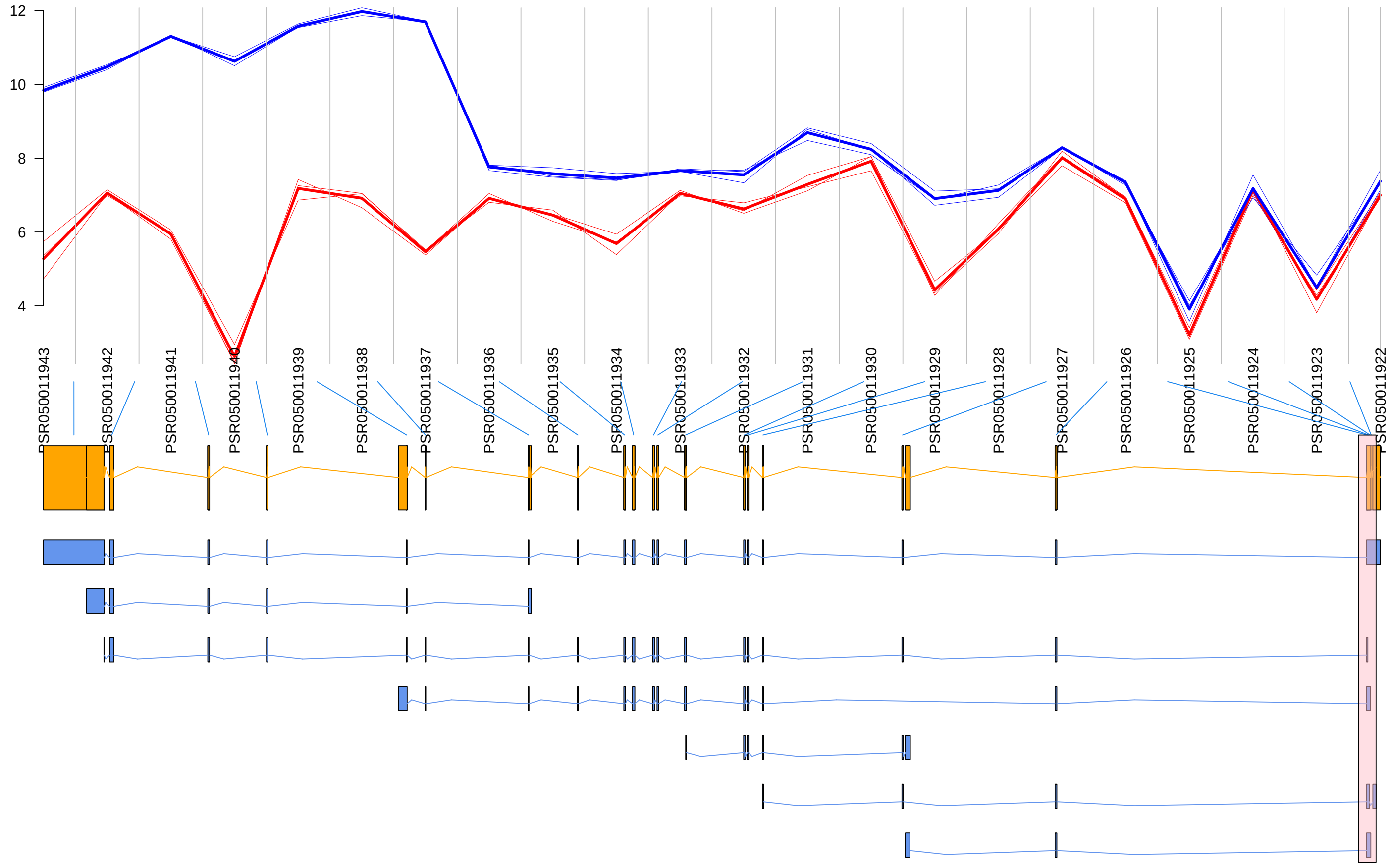

Figure S 36: The isoform composition of transcript cluster TC0501406 (CAMK2A). Probe set PSR050011925 is highlighted as an alternative last event exon. The red lines represent the probe set expression levels of the liver samples. The blue lines show the probe set expression levels for the muscle samples.

Figure S37 illustrates the possible isoform transcriptions of TC0501406 as identified with the microarray. In this transcript, many more probe sets are identified as AS by the REIDS model. This is in concordance with the expression levels illustrated in Figure S36

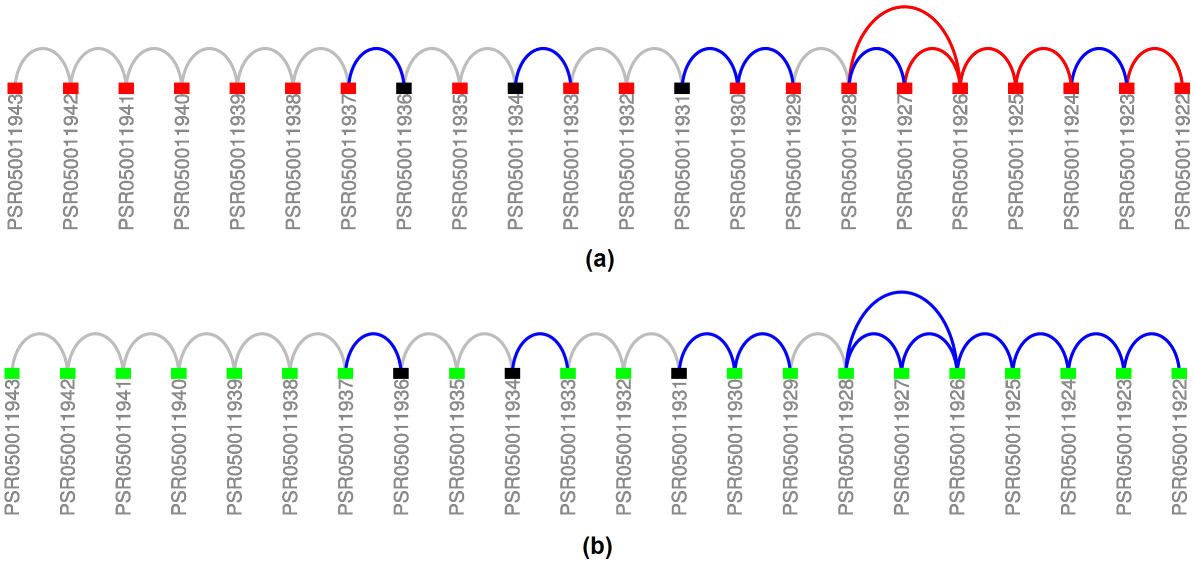

Figure S 37: The gene design for transcript cluster TC0501406 (CAMK2A). Black probe sets are identified as constituitive while coloured probe sets were identified as AS. A green colour indicates a enrichment of the probe sets and a red colour a depletion. Junction which are DABG are shown in blue while depleted junctions are again shown in red. Grey linkages are illustrative and have no meaning matching the measured data. Panel (a) shows the probe sets for the liver samples and panel (b) for the muscle samples.

3.8 Comparison with Other Software

Table S3 shows the top 10 identified probe sets of the AltAnalyze software and the corresponding ranks in REIDS and TAC.

Table S 3: Comparison of the ranks of the top 10 identified AS probe sets by the AltAnalyze in REIDS and TAC.

Rank in AltAnalyze	Gene	Probe Set	ASPIRE Score	Event Type	Rank in REIDS	Rank in TAC
1	TC08000250	PSR08003572	-0.72	altPromoter	348	26
2	TC12000810	PSR12010315	0.682	alt-5'	1192	75
3	TC08000225	PSR08003276	0.606	alt-C-term		385 94
4	TC12000795	PSR12010065	0.584	cassette-exon	113	174
5	TC22000195	PSR22003613	-0.52	cassette-exon	325	1612
6	TC22000195	PSR22003598	-0.51	cassette-exon	46	1690
7	TC21000265	PSR21003311	-0.5	cassette-exon	287	1783
8	TC13000453	PSR13005144	0.48	-	-	18998
9	TC13001341	PSR13011991	0.48	-	-	23624
10	TC08000225	PSR08003288	0.471	alt-C-term	-	51047

Table S4 shows the top 10 identified probe sets of the TAC software and the corresponding ranks in REIDS and AltAnalyze.

Table S 4: Comparison of the ranks of the top 10 identified AS probe sets by the TAC in REIDS and AltAnalyze.

Rank in TAC	Probe Set	Gene	SI Score	Event Type	Rank in REIDS	Rank in AltAnalyze
1	TC06001355	PSR06016183	-8.60		6	-
2	TC12000082	PSR12000896	5.78	Alternative 5' Donor Site	7	-
3	TC11002681	PSR11030587	-2.24		53	-
4	TC02001126	JUC02009171	-6.35		-	-
5	TC05000942	PSR05013777	3.41	Alternative Last Exon	-	-
6	TC12001377	PSR12017926	2.46		-	-
7	TC22001427	PSR22005794	-3.53	Cassette Exon	-	-
8	TC02001126	PSR02017101	-3.86	Complex Event	1314	-
9	TC09000463	PSR09004941	4.25	Cassette Exon	22	-
10	TC05002135	PSR05029781	-2.13		368	-

Figure S38 shows the junction architecture for probe set PSR12000150 of transcript cluster TC12000010 (WNK1). It has two annotated 5′end junctions (JUC12000126 and JUC12000107), one 3′end junction (JUC12000123) and two exclusion junction (JUC12000111 and JUC12000114). Figure S39 presents the expression levels of the probe sets and its annotated junctions.

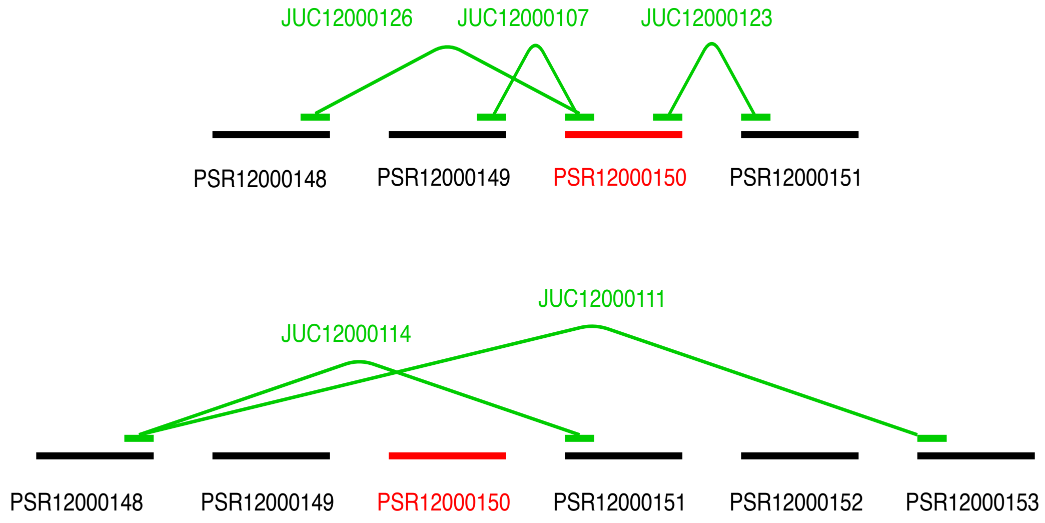

Figure S 38: The junction design of probe set PSR12000150 of transcript cluster TC12000010 (WNK1). Panel (a): The design of the 5′ and 3′ linking junctions of probe set PSR17017166 . Panel (b): The design of the exclusion junctions of probe set PSR12000150.

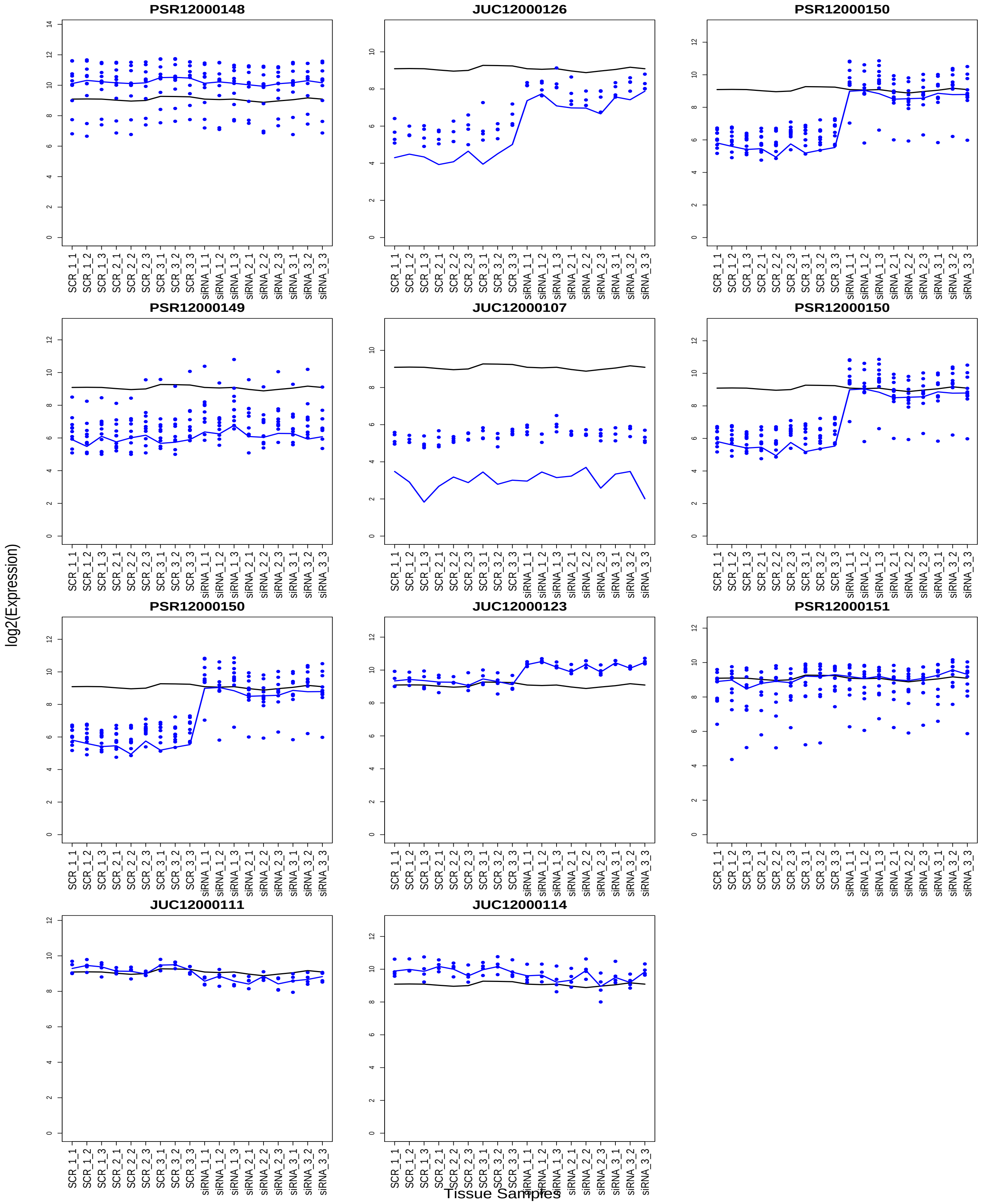

Figure S 39: An example of highly ranked AS probe set in REIDS, TAC and AltAnalyze identified between the SCR and siRNA samples: PSR12000150 of transcript cluster TC12000010 (WNK1). The junction architecture of PSR12000150 is shown in Figure S38. The observed probe intensities of PSR12000148, JUC12000126, PSR12000150, PSR12000149, JUC12000107, PSR12000150, JUC12000123, PSR12000151, JUC12000111 and JUC12000117 are presented relative to the summarized gene level values of TC12000010. The black and blue lines indicate the mean profiles of the gene and exon level data respectively. The blue points show the probe level data.

Figure S40 shows the junction architecture for probe set PSR01003418 of transcript cluster TC01000205 (SZRD1 gene). It has two annotated 3′ junctions (JUC01001841 and JUC01001832), five annotated 5′end junctions (JUC01001830, JUC01001833, JUC01001837, JUC01001838 and JUC01001845) and three exclusion junction (JUC01001835, JUC01001842 and JUC01001844). Figure S41 and S42 presents the expression levels of the probe sets and its annotated junctions.

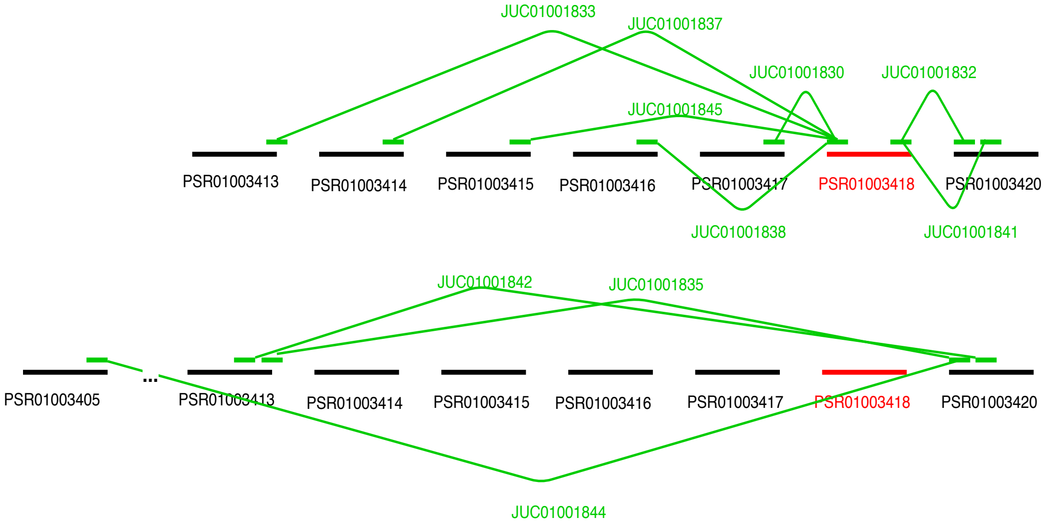

Figure S 40: The junction design of probe set PSR01003418 of transcript cluster TC01000205 (SZRD1). Panel (a): The design of the 3′ linking junctions of probe set PSR01003418. Panel (b): The design of the exclusion junctions of probe set PSR01003418.

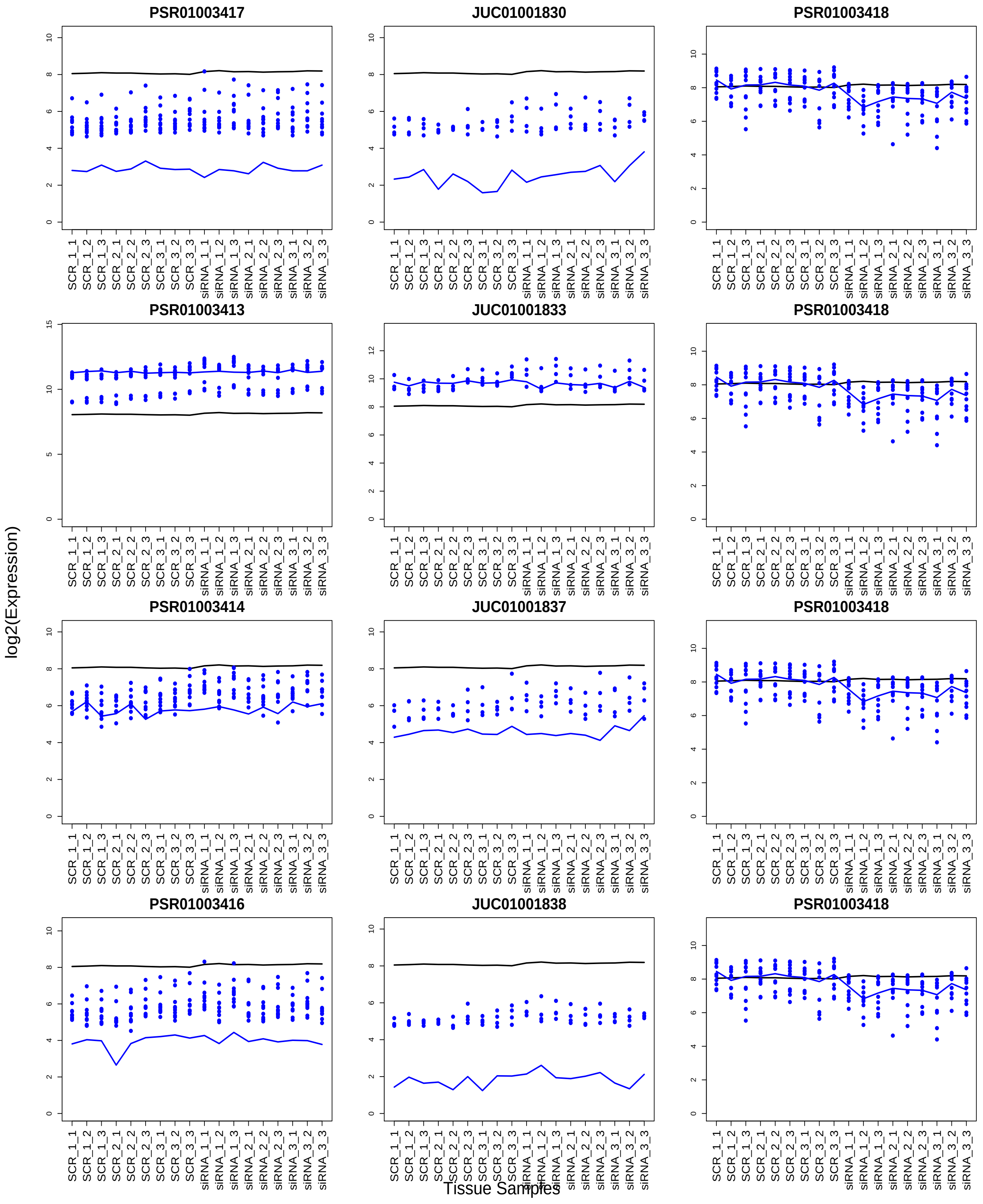

Figure S 41: An example of highly ranked AS probe set in REIDS but lower ranked in TAC and AltAnalyze identified between the SCR and siRNA samples: PSR01003418 of transcript cluster TC01000205 (SZRD1). The junction architecture of PSR01003418 is shown in Figure S40. The observed probe intensities of PSR01003417, JUC01001830 PSR01003418, PSR01003413, JUC01001833, PSR01003418, PSR01003414, JUC01001837, PSR01003418, PSR01003416, JUC01001838 and PSR01003418 are presented relative to the summarized gene level values of TC01000205. The black and blue lines indicate the mean profiles of the gene and exon level data respectively. The blue points show the probe level data.

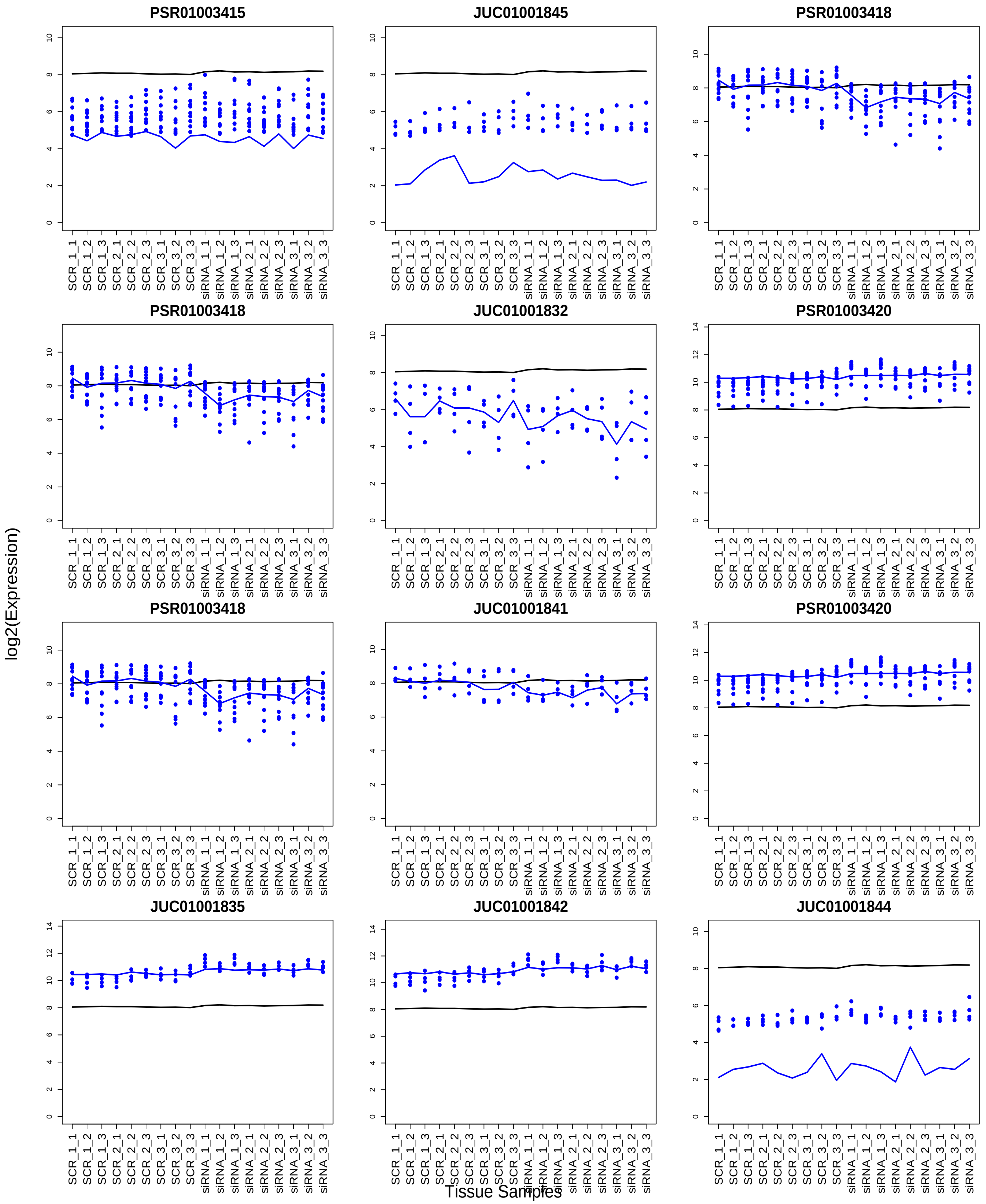

Figure S 42: An example of highly ranked AS probe set in REIDS but lower ranked in TAC and AltAnalyze identified between the SCR and siRNA samples: PSR01003418 of transcript cluster TC01000205 (SZRD1). The junction architecture of PSR01003418 is shown in Figure S40. The observed probe intensities of PSR01003415, JUC01001845, PSR01003418, PSR01003418, JUC01001832, PSR01003420, PSR01003418, JUC01001841, PSR01003420, JUC01001841, JUC01001842 and JUC01001844 are presented relative to the summarized gene level values of TC01000205. The black and blue lines indicate the mean profiles of the gene and exon level data respectively. The blue points show the probe level data.

Figure S43 shows the junction architecture for probe set PSR17017175 of transcript cluster TC17001298 (SPAG5). It has an annotated 3′end junction (JUC17009694), an annotated 5′end junction (JUC17009737) and one exclusion junction (JUC17009713). Figure S44 presents the expression levels of the probe sets and its annotated junctions.

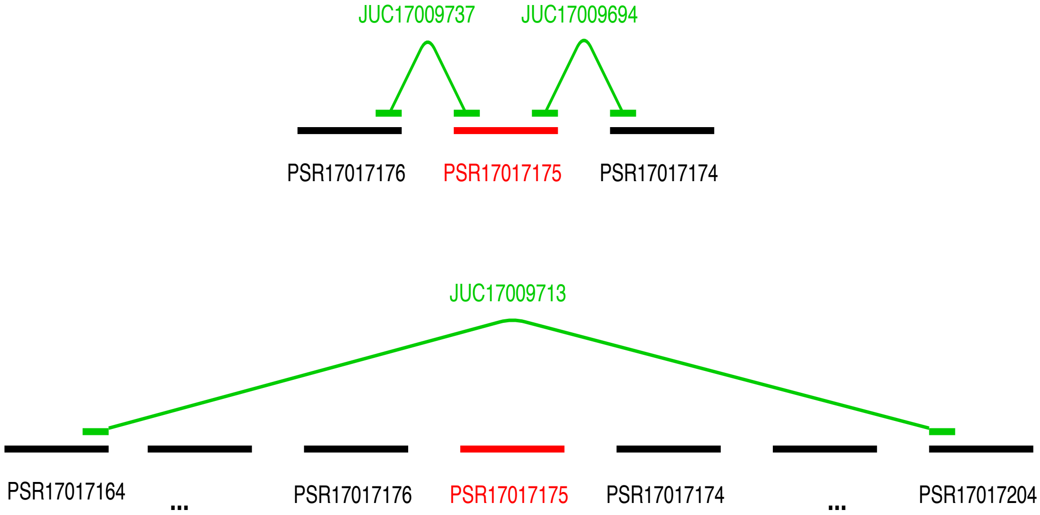

Figure S 43: The junction design of probe set PSR17017175 of transcript cluster TC17001298 (SPAG5). Panel (a): The design of the 3′ and 5′ linking junctions of probe set PPSR17017175. Panel (b): The design of the exclusion junctions of probe set PSR17017175.

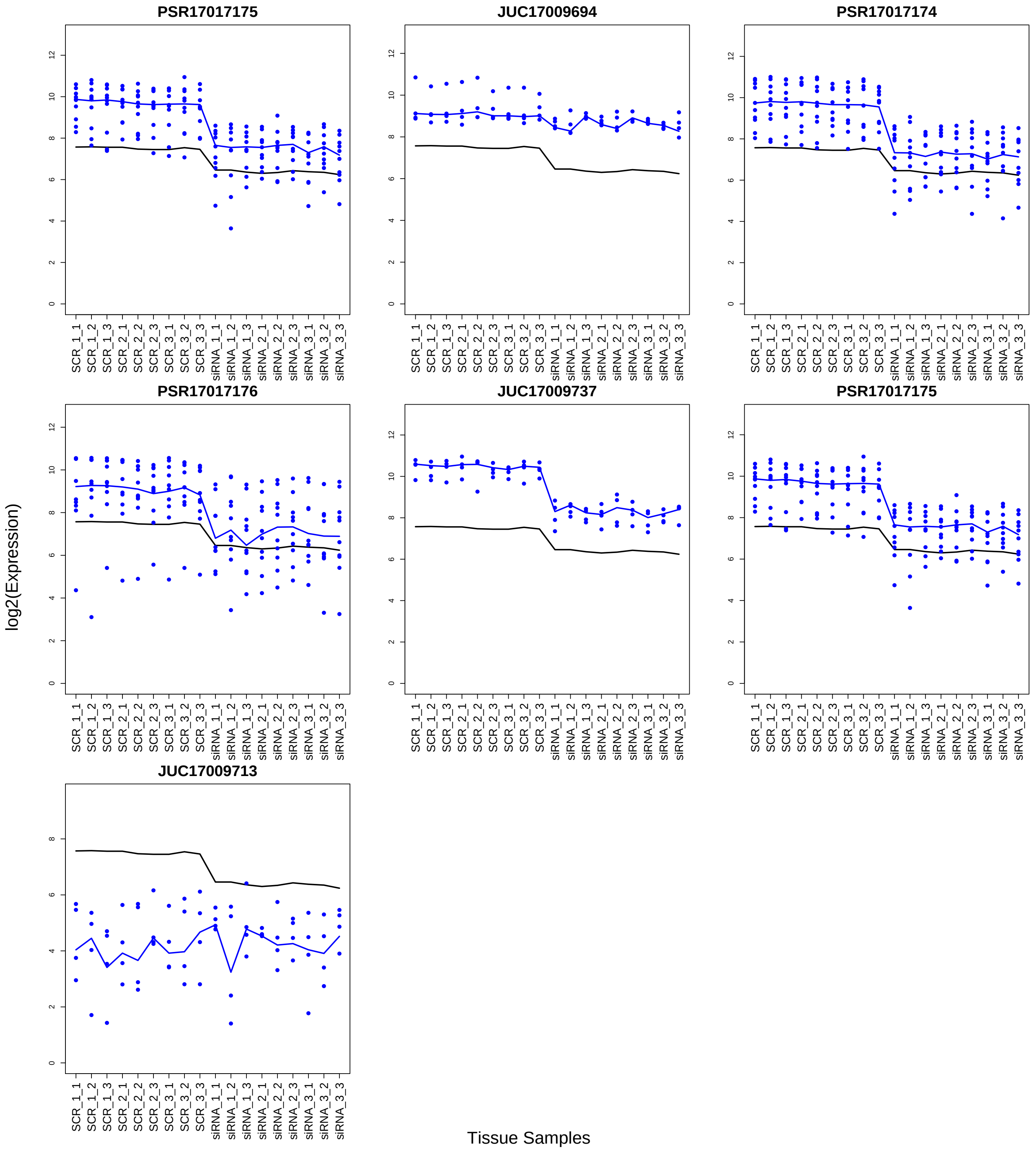

Figure S 44: An example of highly ranked AS probe set in REIDS but lower ranked in TAC and AltAnalyze identified between the SCR and siRNA samples: PSR17017175 of transcript cluster TC17001298 (SPAG5). The junction architecture of PSR17017175 is shown in Figure S43. The observed probe intensities of PSR17017175, JUC17009694, PSR17017174, PSR17017176, JUC17009737, PSR17017175 and JUC17009713 are presented relative to the summarized gene level values of TC17001298. The black and blue lines indicate the mean profiles of the gene and exon level data respectively. The blue points show the probe level data.

References

[1] Affymetrix (2007), “Identifying and validating alternative splicing events,” *Affymetrix Technical Notes*.

[2] Blencowe, B. J. (2006), “Alternative Splicing: New Insights from Global Analyses,” *Cell*, 126, 37–47.
